# Exploring divalent conjugates of 5-*N*-acetyl-neuraminic acid as inhibitors of coxsackievirus A24 variant (CVA24v) transduction†

**DOI:** 10.1039/d1ra08968d

**Published:** 2022-01-14

**Authors:** Emil Johansson, Rémi Caraballo, Georg Zocher, Nitesh Mistry, Niklas Arnberg, Thilo Stehle, Mikael Elofsson

**Affiliations:** Department of Chemistry, Umeå University SE90187 Umeå Sweden mikael.elofsson@umu.se; Interfaculty Institute of Biochemistry, University of Tübingen 72076 Tübingen Germany; Department of Clinical Microbiology, Umeå University SE90185 Umeå Sweden; Vanderbilt University School of Medicine Nashville Tennessee 37232 USA

## Abstract

Coxsackievirus A24 variant (CVA24v) is responsible for several outbreaks and two pandemics of the highly contagious eye infection acute hemorrhagic conjunctivitis (AHC). Currently, neither prevention (vaccines) nor treatments (antivirals) are available for combating this disease. CVA24v attaches to cells by binding Neu5Ac-containing glycans on the surface of cells which facilitates entry. Previously, we have demonstrated that pentavalent Neu5Ac conjugates attenuate CVA24v infection of human corneal epithelial (HCE) cells. In this study, we report on the structure-based design of three classes of divalent Neu5Ac conjugates, with varying spacer lengths, and their effect on CVA24v transduction in HCE cells. In relative terms, the most efficient class of divalent Neu5Ac conjugates are more efficient than the pentavalent Neu5Ac conjugates previously reported.

## Abstract 2

Coxsackievirus A24 variant (CVA24v) is responsible for several outbreaks and two pandemics of the highly contagious eye infection acute hemorrhagic conjunctivitis (AHC).

## Graphical abstract

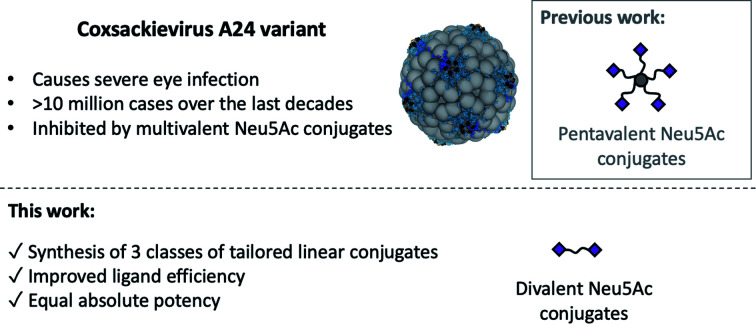

## Introduction

Acute haemorrhagic conjunctivitis (AHC) is a highly contagious eye infection.^1^ It is predominantly caused by two members of the *Picornaviridae* family, coxsackievirus A24 variant (CVA24v), an antigenic variant of the CVA24 strain, and enterovirus 70 (EV70).^2^ The disease may also be caused by human adenoviruses. However, since its emergence in 1970, CVA24v has been reported as the principal etiological agent.^3,4^ AHC is characterized by a sudden onset of ocular pain, foreign body sensation, excessive lacrimation, periorbital swelling and subconjunctival hemorrhages.^5^ The disease spreads rapidly within communities, affecting up to 50% of the population. Such outbreaks can exhaust local healthcare resources of affected regions and severely disrupt the economy. In otherwise healthy individuals, AHC is generally self-limiting, resolving in 1–2 weeks. However, the infections are also associated with visual impairments, symptoms in the respiratory tract, and in rare cases cause acute flaccid paralysis and fatalities.^1^ Over the last decades CVA24v has caused two pandemics, numerous recurring outbreaks, and caused >10 million cases of AHC worldwide.^1^ Neither antiviral agents nor vaccines are currently available for treating or preventing the disease as is the case for most viruses causing diseases in humans.^6^

Members of the *Picornaviridae* engage a range of different cellular receptors facilitating attachment and entry. These include coxsackievirus and adenovirus receptor (CAR), decay accelerating factor (DAF, CD55), low density lipoprotein receptor (LDL-R), human P-selectin glycoprotein ligand-1 (PSGL-1), heparan sulfate, integrins, intercellular adhesion molecule-1 (ICAM-1), and glycan-containing receptors terminating in 5-*N*-acetyl-neuraminic acid (Neu5Ac).^7-11^ CVA24v is reported to engage receptors with terminal α2,3- and α2,6-linked Neu5Ac, with some preference for the α2,6-linkage.^9,10,12^ In addition, binding to the ICAM-1 receptor is essential for productive replication of CVA24 and the AHC-causing CVA24v.^13^ However, the enhanced ability of CVA24v to bind Neu5Ac has been suggested to contribute to its notable virulence and pandemic potential. Thus, Neu5Ac-based derivatives have potential to prevent attachment of virions to cells, limiting the infection and subsequent spread.

Protein-carbohydrate interactions play essential roles in a vast array of biological processes. However, only a few carbohydrate and carbohydrate-based drugs have reached the market. This is perhaps due to the challenges associated with overcoming the poor pharmacological properties of carbohydrates imparted by their high polarity and metabolic vulnerability.^14^ In addition, protein–carbohydrate interactions are generally of low affinity.^15^ These downsides are not of direct consequence for the development of carbohydrate-based therapeutics in the case of CVA24v as its primary sites of replication in humans are linked to the eyes and airways.^10^ Thus, by design of multivalent ligands intended for topical administration many of these downsides can potentially be circumvented. Furthermore, the design of multivalent carbohydrate ligands is an excellent strategy to achieve high avidity binding to lectins *via* the “glycoside cluster effect” or proximity/statistical effects.^15^ Particularly, dramatic enhancements in avidity have been observed by tailoring the multivalent carbohydrate ligands to lectin valency and topology.^16^

Currently, multivalent Neu5Ac-based ligands have been reported to inhibit CVA24v attachment^10,17^ and infection^17,18^ of human corneal epithelial cells. The most efficient, in terms of potency and ease of preparation, are pentavalent Neu5Ac conjugates based on a glucose scaffold.^17^ Herein, we report on the synthesis of divalent Neu5Ac compounds that are of comparable potency to the previously reported pentavalent conjugates and thus more efficient in terms of relative inhibitory potency per Neu5Ac unit.

## Results and discussion

### Design

**Fig. 1:**
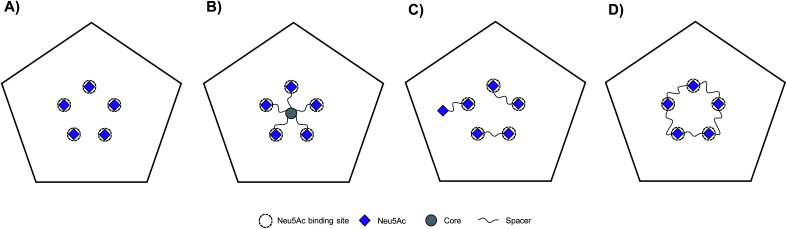
Schematic representation of Neu5Ac binding sites of CVA24v pentagon and topology of multivalent interactions. CVA24v pentagon in complex with Neu5Ac monosaccharides (A), an inhibitor with pseudoradial topology (B), dimeric inhibitors with linear topology (C), and a macrocyclic pentavalent inhibitor (D).

CVA24v attaches to cells *via* shallow, surface exposed, Neu5Ac pockets.^12^ The CVA24v capsid is decorated by a total of sixty individual Neu5Ac binding sites which are further arranged into twelve local five-fold symmetries (*i.e.* twelve regular pentagons). Thus, at its most basic form the template for the design of multivalent Neu5Ac-inhibitors can be based on a single pentagon with its five Neu5Ac binding sites (Fig. 1A). We have previously reported on the design and synthesis of pentavalent Neu5Ac conjugates of radial^18^ and pseudoradial^17^ topology (Fig. 1B), that inhibit CVA24v infection of HCE cells. X-ray crystallography confirmed inhibitor binding, with observed electron density for the Neu5Ac units while spacers and the respective scaffold were not detected. The major mode of inhibition of these inhibitors likely resulted from a high local concentration of ligands, and inhibitor caused aggregation of viral particles as indicated by negative staining electron microscopy (EM)^17^ and cryogenic electron microscopy (cryo-EM),^18^ rather than intended fully chelated binding sites. Building on these observations we hypothesized that multivalent Neu5Ac inhibitors based on tailored linear topology, could engage in additional contacts with the virion through spacer fragments thus generating more potent inhibitors.

As a starting point to probe this strategy, divalent Neu5Ac compounds posed as attractive tools as they have potential to bind to the virion by simultaneously occupying two Neu5Ac binding sites. The spacers connecting both Neu5Ac entities can engage in additional binding events with the protein backbone of the virion (Fig. 1C). Despite the inherent synthetic challenges, a linear strategy has potential to be expanded into linear trivalent, tetravalent, or eventually macrocyclic pentavalent structures (Fig. 1D).

**Fig. 2:**
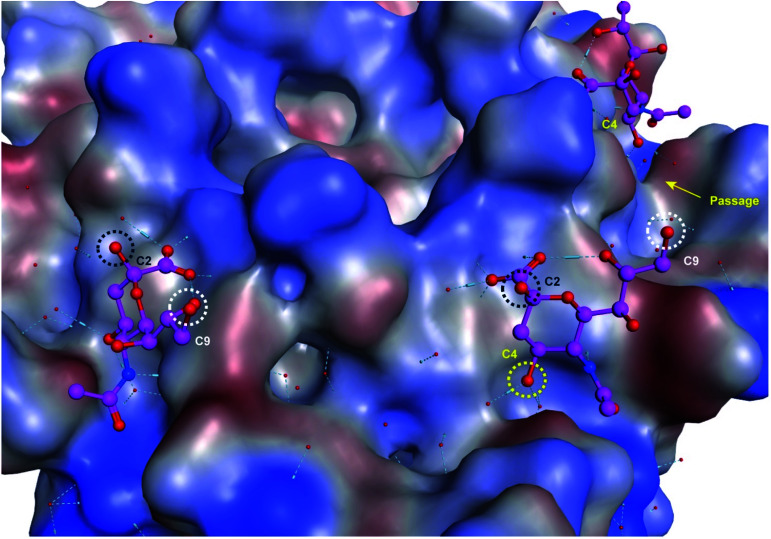
Neu5Ac bound to CVA24v pentagon with highlighted anchoring positions. The hydroxyl groups attached to C2 (black), C4 (yellow), and C9 (white) are highlighted and serve as excellent connection points for constructing divalent Neu5Ac compounds.

Upon examination of the Neu5Ac binding sites of CVA24v of Neu5Ac bound (PDB code 4Q4Z) and inhibitor bound (PDB code 6TSD) structures, it was evident that the closest points of contacts between two neighbouring Neu5Ac residues were from the C9 position of one Neu5Ac residue to the C2 or C4 position of a second Neu5Ac residue (Fig. 2). The hydroxyls attached to C2 and C9 serve as promising connection points as they are pointing towards the solvent, thereby minimizing possible steric clashes with the virion, and are not key contributors to Neu5Ac binding.

Both these hydroxyl groups are also directed towards a shallow canyon that interconnects the five Neu5Ac binding sites of each pentagon, thus opening up the possibility of the spacer binding to amino acid residues of the canyon. Further, both C2 and C9 Neu5Ac alkynyl or azido building blocks are readily accessible and can be conjugated under mild conditions (*e.g.* copper azide alkyne cycloaddition) to provide access to C2–C9 linked divalent Neu5Ac compounds. Contrarily, the C4 hydroxyl is engaged in two water mediated hydrogen bonds to the protein backbone of the virion and projects towards the protein. However, the canyon that interconnects the Neu5Ac binding sites is directly accessible from the C4 hydroxyl *via* a narrow passage making it highly interesting as a point of connection as interactions to, or steric clashes with, the protein backbone of the virion are forced. Further, 4-*O*-alkynyl Neu5Ac building blocks are accessible *via* etherification of appropriately protected building blocks^19^ and after deprotection conjugated to a C9 modified azido Neu5Ac compound to yield C4–C9 linked divalent Neu5Ac compounds. Another, more straightforward method, of assembling divalent Neu5Ac compounds is preparation of a common Neu5Ac building block, *e.g.* alpha-azido Neu5Ac,^20^ and subsequently conjugate this to a di-alkynyl spacer fragment to yield C2–C2 triazole linked divalent Neu5Ac compounds. The spacer fragments of such divalent C2–C2 linked Neu5Ac compounds are however less likely to bind to the canyon as alpha anomeric Neu5Ac substituents are pointing towards the solvent. Nevertheless, we decided to probe all three strategies.

### Synthesis of divalent Neu5Ac compounds 16–21, 32–34, 40 and 41

**Scheme 1:**
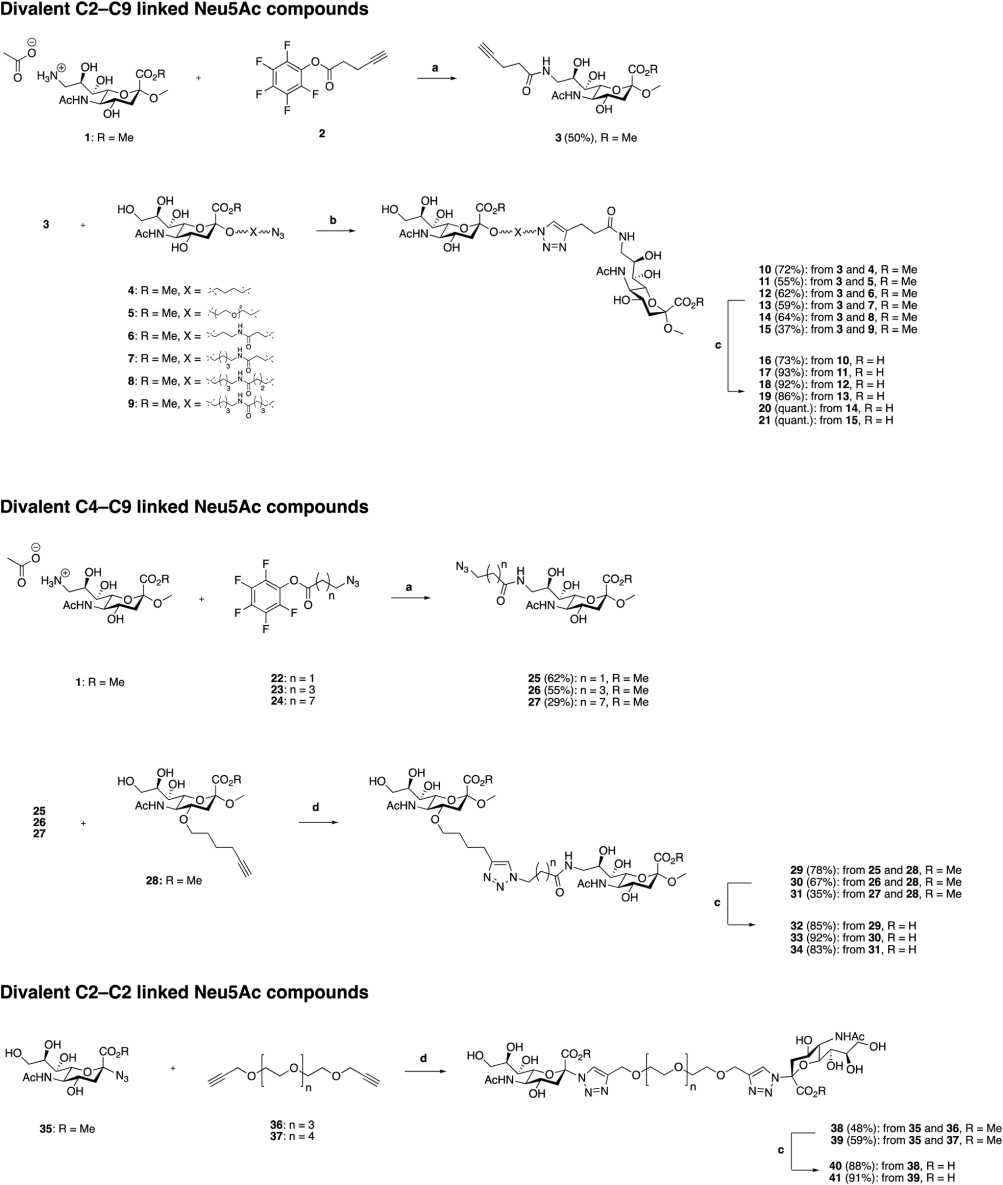
Synthesis of divalent Neu5Ac compounds 16–21, 32–34, 40 and 41. Reagents and conditions: (a) (i) pyridine, *N*,*N*′-dimethylaminopyridine (DMAP), DCM, molecular sieves (3 Å), rt ≈ 1 h (until all of 1 is dissolved), then Pfp ester 22, 23, or 24, respectively, (ii) sodium methoxide, methanol, rt, 16 h, (iii) H^+^ ion exchange resin. (b) CuSO_4_·5H_2_O, sodium ascorbate, tetrahydrofuran/H_2_O (1 : 1), microwave irradiation 100 °C, 2 h, or 50 °C, 5 h → rt, 18 h. (c) (i) LiOH, methanol, rt, 24 h, (ii) H^+^ ion exchange resin.

The three classes of divalent Neu5Ac compounds were synthesized in two to four steps from known building blocks (Scheme 1). Synthesis of the C2–C9 linked class of divalent Neu5Ac compounds 16–21 started from the amino acetate salt 1,^21^ that was reacted with pentafluorophenyl (Pfp) ester 2^22^ in dichloromethane (DCM) and pyridine, using 4-dimethylaminopyridine (DMAP) as a catalyst, to afford a crude mixture of product 3 in addition to differently *O*-acetylated species. Subsequent *O*-deacetylation afforded pure 3 in 50% yield over the two steps. Compound 3 was then “clicked” to the azido building blocks 4–9,^17^ respectively, affording the corresponding C2–C9 linked divalent sialic acid methyl esters 10–15 in 24–72% yields. Subsequent saponification provided the corresponding C2–C9 linked divalent sialic acid compounds 16–21 in 73% to quantitative yields after neutralization. The synthetic strategy to synthesis the C4–C9 linked class of divalent compounds 32–34 was analogous with minor exceptions. That is, Pfp esters 22,^22^23,^23^ and 24 were used, respectively, in the amide coupling to compound 1 to afford azido derivates 25–27 in 29–62% yield over the two steps, including *O*-deacetylation. The azido containing derivatives 25–27 were then, respectively, “clicked” to the alkyne building block 28^19^ affording the corresponding C4–C9 linked divalent sialic acid methyl esters 29–31 in 35–78% yields. Subsequent saponification provided the corresponding C4–C9 linked divalent Neu5Ac compounds 32–34 in 83–92% yields after neutralization. Synthesis of the C2–C2 linked class of divalent Neu5Ac compounds started from the alpha azido sialoside 35,^20^ that was clicked onto the alkynyl-polyethyleneglycol (PEG) spacers 36^24^ and 37 ^24^ affording the corresponding divalent sialic acid methyl esters 38 and 39 in 48% and 59% yields, respectively. Subsequent saponification and neutralization provided the C2–C2 linked divalent Neu5Ac compounds 40 and 41 in 88% and 92% yields, respectively.

### C2–C9 linked divalent Neu5Ac compounds reduce CVA24v transduction

**Fig. 3:**
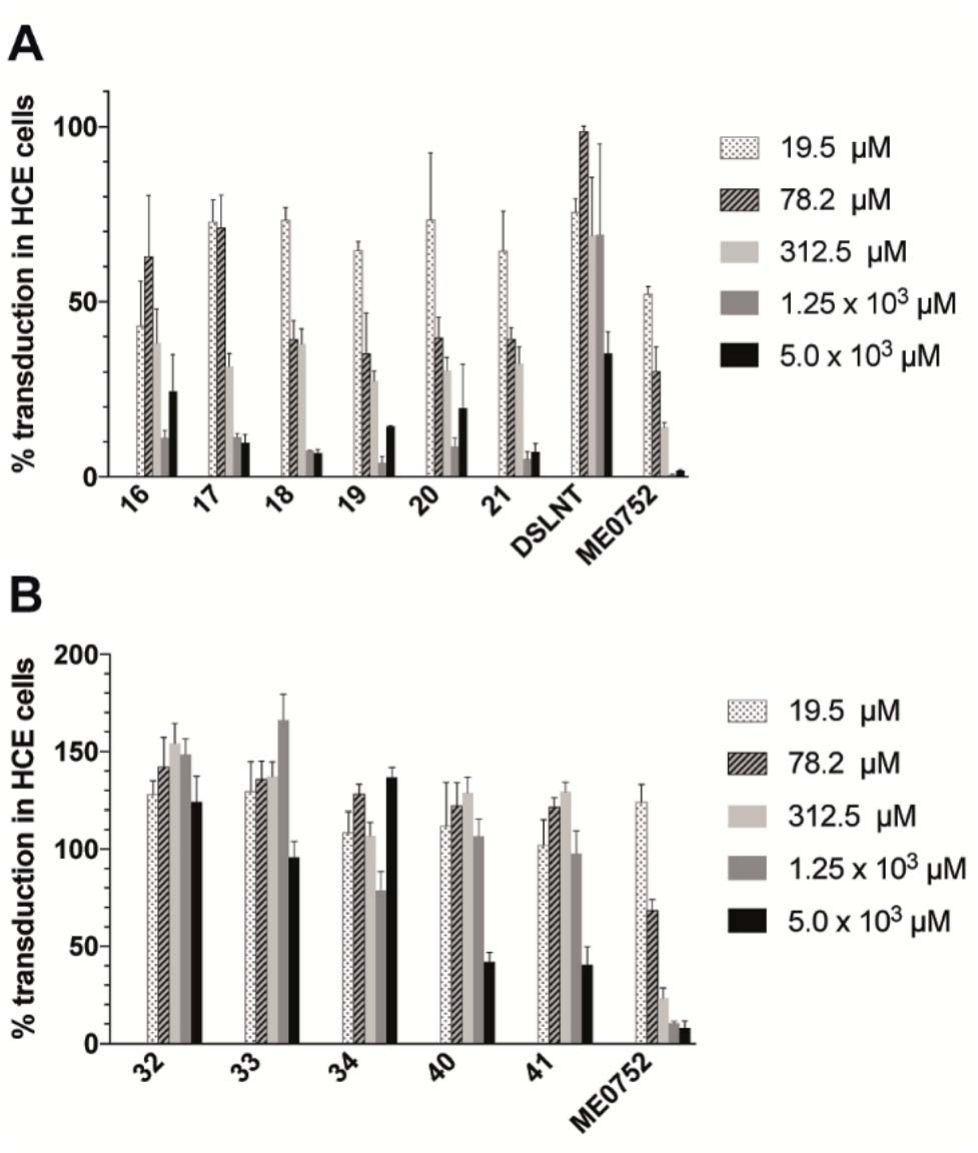
Effect of C2–C9, and C4–C9 and C2–C2 divalent Neu5Ac compounds on CVA24v transduction. Transduction of CVA24v at 37 °C with different concentrations of divalent Neu5Ac inhibitors. 100% corresponds to transduction in non-treated infected cells. (A) C2–C9 linked Neu5Ac inhibitors 16–21, DSLNT and ME0752. (B) C4–C9 linked Neu5Ac inhibitors 32–34 and C2–C2, linked Neu5Ac inhibitors 40 and 41. Error bars are shown as the standard error of the mean (SEM). Data are presented as the % of control that is the value obtained in the absence of an inhibitor. All experiments were performed two times in duplicates.

The antiviral effect of the three synthesized classes of divalent Neu5Ac compounds were evaluated by measuring CVA24v transduction using HCE cells.^17^ Ocular inspection of cells treated with divalent compounds did not reveal any cytotoxicity at any of the tested concentrations. Compared with untreated virions (control), the transduction was significantly attenuated when preincubated with compounds 16–21 at all of the measured compounds concentrations (Fig. 3A). This is in contrast to Neu5Ac, that failed to reduce CVA24v transduction at 10 mM (not shown)^12,17^ and the natural occurring divalent Neu5Ac glycan disialyllacto-*N*-tetraose (DSLNT) that reduced CVA24v transduction by 65% at 5 mM (Fig. 3A). Compounds 18 and 21 seemed most efficient at reducing transduction >90% at 5 and 1.25 mM, while compounds 16, 17, 19 and 20 were slightly less efficient and reduced transduction by 75–95%. For comparison the pentavalent Neu5Ac derivative ME0752 (compound 28 in ref. 17) essentially abolished (>98%) transduction at both 5 and 1.25 mM in agreement with previously published data.^17^ At 312.5 and 19.5 μM compounds 17–21 were equally effective and reduced transduction by 60–70% and 30–35%, respectively, while ME0752 reduced transduction by 85% and 50%, respectively. At 78.2 μM compounds 18–21 were ≥1.5 times more efficient than 16 and 17 and reduced transduction by 60%, while ME0752 reduced transduction by 75%. Taken together, a relationship between spacer length and potency could not be demonstrated due to small differences in efficacy between the compounds. This is in agreement with previous reports that have demonstrated that the length of flexible spacers in divalent ligands have minimal influence on avidity.^25,26^ Nevertheless, compounds 18 and 21 seemed to be among the top performers at each of the tested concentrations. Further, in terms of absolute values ME0752 was the most potent inhibitor. However, in terms of relative inhibitor potency, the ratio of reduced transduction produced by an inhibitor divided by the number of Neu5Ac units present in the inhibitor or the number of heavy atoms in the molecule, 16–21 were more efficient at reducing CVA24v transduction.

Contrarily, the C2–C2 linked compounds 40 and 41 were only effective at reducing CVA24v transduction by 60% at 5 mM (Fig. 3B). The C4–C9 linked compounds (compounds 32–34) did not attenuate CVA24v transduction at any of the tested concentrations (Fig. 3B), indicating the C4 hydroxyl as a key contributor in the binding and recognition of Neu5Ac by CVA24v or alternatively that steric factors imposed by the spacer prohibit binding. Thus, only the C2–C9 linked Neu5Ac compounds were considered for further evaluation.

### X-ray crystallography support binding of the C2–C9 divalent Neu5Ac compounds to CVA24v

**Fig. 4:**
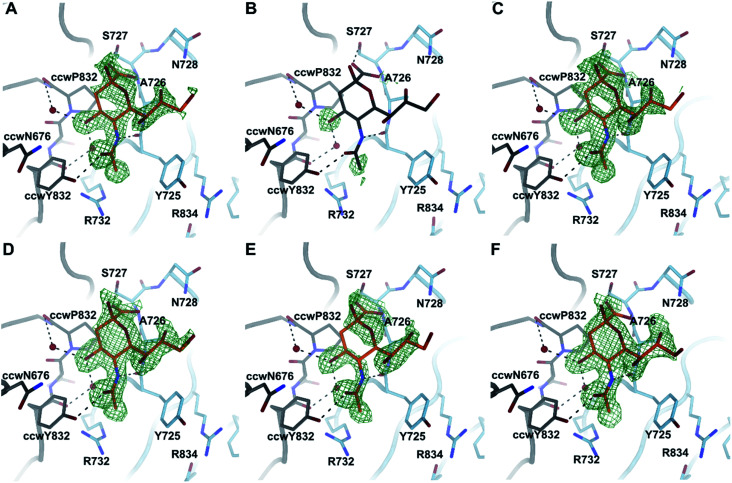
Binding of compounds 16–21 to CVA24v. Beside 17 (B) all compounds accommodate the Neu5Ac binding site located at the interface between the viral capsid protein VP1 and its counterclockwise (ccw) rotated oligomer at the five-fold symmetry of the icosahedral capsid. The conformations of all binders 16 (A), 18 (C), 19 (D), 20 (E) and 21 (F) are virtual identical to Neu5Ac attachment. Binding is illustrated by an unbiased difference electron density (green) shown at a contour level of 2.8 *σ* and the Neu5Ac entity shown in orange. For 17 (B) Neu5Ac (grey) is placed into the binding site to enhance the readability of the figure.

To investigate the binding of compounds 16–21, we derivatized CVA24v by soaking CVA24v crystals in crystallization solution containing the corresponding compound (16 mM) as described previously.^12^ The following structure determination resulted in data sets with resolutions below or around 2 Å and showed that all compounds, beside 17, bind by their Neu5Ac entity to the characterized binding site.^12^ The linker regions of all compounds seem not to interact with the capsid protein and are therefore not visible in the resolved X-ray crystal structures (Fig. 4). A plausible reason is the C2–C9 linkage of these dimeric compounds. The C2 and C9 hydroxyls of the parent Neu5Ac molecule are pointing towards the solvent. Thus, we find it likely that the spacer unit that links the C2 position of one Neu5Ac entity to the C9 position of a second Neu5Ac entity is floating in the solvent in a non-ordered fashion rather than folding back towards the viral surface to interact with the canyon on top of the VP1 capsid protein.

### C2–C9 divalent Neu5Ac compounds do not affect particle stability of CVA24v

**Fig. 5:**
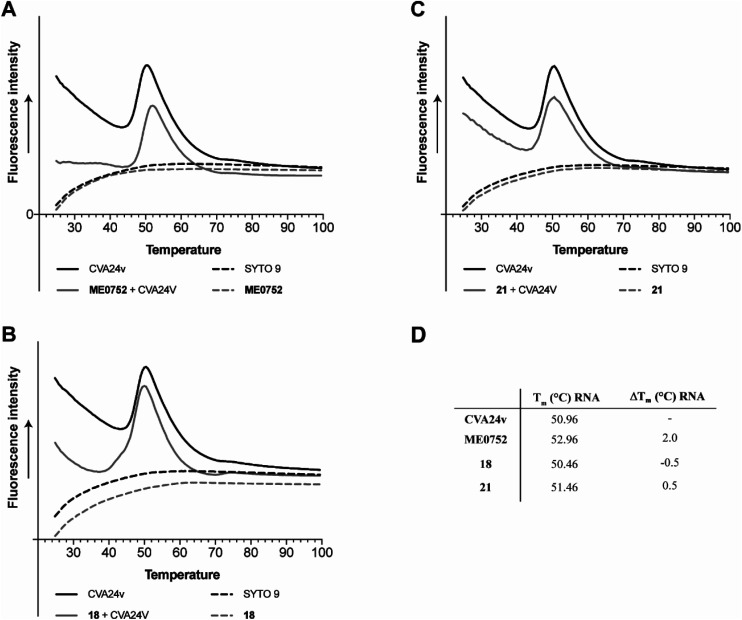
Effect of C2–C9 linked divalent Neu5Ac conjugates on thermal stability of CVA24v as measured by release of viral RNA (*T*_m_ RNA). (A) Effect of ME0752,^17^ a pentavalent Neu5Ac conjugates, (B) effect of 18, (C) effect of 21, (D) summary of CVA24v *T*_m_ RNA and *T*_m_ shifts RNA (Δ*T*_m_ RNA) as measured for the release of viral RNA (measured by SYTO 9) with and without treatment of 100 μM ME0752, 18, or 21. Fluorescence curves of CVA24v with (solid grey line) and without (solid black line) inhibitor. The dashed lines correspond to fluorescence observed with inhibitors (ME0752, 18 or 21) in the absence of CVA24v (black), and fluorescence of the SYTO 9 stain (grey line) also in absence of CVA24v.

Capsid binding antivirals have been suggested to act by stabilization of the virus particle,^27^ preventing or delaying receptor-mediated conformational changes required for uncoating.^28^ In analogy, capsid-binding molecules could also mimic the receptor-mediated interaction thus triggering conformational changes resulting in early uncoating and inactivation of the virus. To study the effect of the inhibitors on the stability of CVA24v, we performed the Particle Stability Thermal Release Assay (PaSTRy).^29^ The C2–C9 divalent inhibitors 18 and 21 were selected for testing together with the pentavalent Neu5Ac conjugate ME0752 (28 in ref. 17). The melting temperature of the CVA24v capsid, as measured by release of viral RNA (*T*_m_ RNA), was calculated to be 50.96 °C, in good agreement with previous observations^17^ and in the same range of other known *Enteroviruses*.^28^ In line with previous observations, ME0752 had a mild stabilizing effect and shifted the release of viral RNA to 52.96 °C (Fig. 5A and D), which is 1.0 °C more than previously reported.^17^ Treatment with 18 and 21 shifted the release of viral RNA to 50.46 °C and 51.46 °C (Fig. 5B–D), respectively. Thus, the divalent Neu5Ac compounds does not seem to affect the thermal stability of the CVA24v particle indicating the mechanism of inhibition is not related to CVA24v uncoating.

## Conclusion

In the present study we employed a structure-based approach to design three classes of novel divalent Neu5Ac conjugates with the aim to achieve chelation binding of the Neu5Ac binding sites of CVA24v. We presented efficient synthesis of each class of compounds, using “click” chemistry in the key chemical transformation connecting the two Neu5Ac entities with flexible spacers. Compounds with a C2–C9 linkage between the two Neu5Ac entities proved more efficacious blocking CVA24v transduction than C4–C9 and C2–C2 linked compounds. In terms of relative efficiency, the C2–C9 linked compounds were better than the previously reported pentavalent Neu5Ac conjugate ME0752. X-ray crystallography confirmed the binding of the C2–C9 linked divalent Neu5Ac compounds, however electron densities were only detected for the Neu5Ac entities. This suggests that the spacers of the compounds do not interact with the protein backbone of CVA24v or that the spacers are of sufficient flexibility to generate largely non-ordered binding events. The effect of designing and synthesizing precise and rigid spacers were previously demonstrated for divalent galactoside ligands targeting LecA.^30^ A similar strategy could be applied to optimize binding of these divalent Neu5Ac ligands to CVA24v. However, based on the results presented here and previous results with pentavalent Neu5Ac attachment inhibitors^17,18^ we conclude that further optimization of divalent Neu5Ac compounds will be challenging.

## Experimental

### Particle stability thermal release assay (PaSTRy)

CVA24v (1 μg) and compounds (1 mM and 100 μM) were incubated for 30 min at room temperature in a total volume of 20 μl sample buffer (10 mM HEPES pH 8.0, 200 mM NaCl) before adding dyes. PaSTRy assay was performed as previously described.^29^ SYPRO red (stock 5000×, Invitrogen) and SYT09 (stock 50 mM, Thermo Fisher Scientific) were diluted 100× in Milli-Q water freshly before each experiment. The dyes were added to the CVA24v + compound samples to total volume of 50 μl sample buffer and the final concentrations of the dyes were 3× of SYPRO red and 5 μM of SYT09. Samples and dyes were added in a microamp optical 96-well reaction plate (Applied Biosystems, California, USA) and ran a real-time PCR system (StepOnePlus, Applied Biosystems). The melting curve was set to increase 1 °C every 15 s (log fluorescence every 1 °C increased), ranging from 25 °C to 99 °C.

### CVA24v transduction assay

One day prior infection, HCE cells (2 × 10^4^ per well) were seeded in a black 96-well plate with transparent bottom. Next day, Neu5Ac conjugates were added to give four-fold dilution series with 5 mM as the highest concentration and plates were incubated at 37 °C with 10 CVA24v per cell (approximately 4 × 10^4^ cells per well) for 1 hour. HCE cells in the black 96-well plate were washed twice with BB2 then incubated with 50 μl of CVA24v (10 CVA24v per cell) + compound mixture per well. After 1 hour incubation at 37 °C, cells were washed to remove non-bound virions and incubated in HCE growth medium for 16–18 hours. After fixation with 99.5% ice-cold methanol, mouse monoclonal antibodies against enterovirus VP1 (DakoCytomation, Glostrup, Denmark) were diluted 1 : 200 in PBS and 50 μl was added per well. After incubation for 1 hour at room temperature, the cells were washed again and incubated with 50 μl Alexa fluor 488-labeled donkey anti-mouse immunoglobulin G (Thermo fisher scientific) (diluted 1 : 400 in PBS) per well at room temperature. One hour later, the cells were washed again, and the numbers of infected cells were quantified using a Trophos system.

### CVA24v crystallography

CVA24v was produced and described previously.^12^ To incorporate 16–21, the compounds where diluted in the crystallization solution and pipetted to crystals. The final concentration of each compound was 16 mM. The crystals were incubated for 1 hour at 4 °C before harvesting and flash-freezing in liquid nitrogen. Data was recorded at beamline I03 at Diamond Light Source Ltd (Didcot, UK). All data sets were reduced by XDS^31^ and scaled to the native data set that was recorded previously. Phasing was performed by applying the phases of the native structure (PDB code 4Q4W) followed by a simulated annealing approach as implemented in PHENIX.^32^ Refinement of the model was done in a cyclic procedure by reciprocal space refinement as implemented in REFMAC5 ^33^ including strict NCS refinement and real space refinement using COOT^34^ The final models were validated using MOLPROBITY.^35^ Figures were generated with PYMOL.^36^ Data statistics and refinement statistics are summarized in Table 1. As all C2–C9 linked compounds essentially show the same complex formation, we picked compound 18 as a reference and deposited the coordinates together with the structure factor amplitudes to the protein data bank (PDB code 7QB5).

**Table 1:** X-ray data collection and refinement statistics^a^

CVA24v:18	
**Data collection statistics**	
Resolution [Å]	50–1.73 (1.83–1.73)
Space group	*I*222
Unit cell [Å]	*a* = 305.80, *b* = 365.76, *c* = 366.95
No. of unique reflections	1918804 (316033)
*R*_meas_ [%]	15.8 (79.0)
CC(1/2) [%]	99.2 (60.4)
Completeness [%]	91.4 (93.3)
Multiplicity	6.6 (3.1)
*I*/*σ* (I)	6.5 (1.4)
Wilson B-factor [Å^2^]	22

**Refinement statistics**	
*R*_fwork_ [%]	16.6
rmsd bond length [Å]	0.005
rmsd bond angle [°]	1.350

**Ramachandran angles**	
Favoured [%]	96.3
Outliers [%]	0.6

a Values for the highest resolution shell are given in parentheses.

### General chemical procedures

^1^H nuclear magnetic resonance (NMR) and ^13^C NMR spectra were recorded with a Bruker DRX-400 spectrometer at 400 MHz and 100 MHz respectively, or with a Bruker DRX-600 spectrometer at 600 MHz and 150 MHz respectively. NMR experiments were conducted at 298 K in D_2_O (residual solvent peak = 4.79 ppm (*δ*_H_)), CD_3_OD (residual solvent peak = 3.31 ppm (*δ*_H_) and 49.00 ppm (*δ*_C_)), or CDCl_3_ (residual solvent peak = 7.26 ppm (*δ*_H_) and 77.16 ppm (*δ*_C_)). Liquid chromatography mass spectrometry (LC-MS) was performed by detecting positive/negative ion (electrospray ionization, ESI) on Agilent 1290 infinity II-6130 Quadrupole using H_2_O/CH_3_CN (0.1% formic acid) as the eluent system or on Agilent 1290 infinity-6150 Quadrupole using YMC Triart C18 (1.9 μm, 20 × 50 mm column) and H_2_O/CH_3_CN (0.1% formic acid) as the eluent system. High resolution mass spectrometry (HRMS) was performed using a Bruker MicroTOF II time of flight (TOF) mass spectrometer with ESI^+^; Tune Mix ESI solution was used for the calibration. Semi-preparative high performance liquid chromatography (HPLC) was performed on a Gilson system HPLC, using a YMC-Actus Triart C18, 12 nm, S-5 μm, 250 × 20.0 mm with a flow rate 20 mL min^−1^, detection at 214 nm and eluent system A: aqueous 0.005% formic acid, and B: CH_3_CN 0.005% formic acid. Column chromatography was performed on silica gel (Merck, 60 Å, 70–230 mesh ASTM). Thin layer chromatography (TLC) were performed on Silica gel 60 F_254_ (Merck) with detection under ultraviolet (UV) light or development with 5% H_2_SO_4_ in ethanol (EtOH) and heat. Automated flash column chromatography was performed using a Biotage® Isolera One system and purchased pre-packed silica gel cartridges (Biotage® SNAP Cartridge, KP-Sil). Freeze drying was performed by freezing the diluted CH_3_CN/water solutions in dry ice-acetone bath and then employing a Scanvac CoolSafe freeze dryer connected to an Edwards 28 rotary vane oil pump. Organic solvents were dried using a SG Water Glass Contour Solvent Systems except CH_3_CN (freshly distilled from CaH_2_) and methanol (MeOH) that were dried over molecular sieves 3 Å. All commercial reagents were used as received. All target compounds were ≥95% pure according to HPLC UV-traces. Statistics were calculated using GraphPad Prism 7 (GraphPad Software, Inc, La Jolla, CA). Microwave irradiation reactions were performed using a Biotage® Initiator microwave synthesizer; temperatures were monitored by an internal infrared (IR) probe; stirring was mediated magnetically and the reaction were carried out in sealed vessels. Automated flash column chromatography was performed using a Biotage® Isolera One system and purchased pre-packed silica gel cartridges (Biotage® SNAP Cartridge, KP-Sil).

### General procedure 1 (GP1): synthesis of Pfp esters 2 and 22–24

To a solution of carboxylic acid (0.5 M) in anhydrous DCM was added Pfp (1.0 equiv.), 1-ethyl-3-(3-dimethylaminopropyl)-carbodiimide (EDCI, 1.5 eq.), and DMAP (0.1 eq.). The mixture was stirred at room temperature for 16 h under nitrogen atmosphere. Following, the reaction mixture was poured into water and extracted with DCM (3 times). The organic layer was washed with brine, dried over anhydrous Na_2_SO_4_, filtered and concentrated under reduced pressure. Semi-purification by column chromatography (ethyl acetate petroleum ether gradients) afforded the Pfp esters 2, and 22–24.

### General procedure 2 (GP2): synthesis of amides 3, and 25–27

Compound 1 (1092 mg, 1.0 eq.) and 3 Å molecular sieves were added to an oven dried microwave vial and placed under a nitrogen atmosphere. To this was added anhydrous DCM (16.0 mL) and an amine base (pyridine or TEA 3.0–5.0 eq.). The mixture was stirred at rt for 1 hour until all of the sugar species were dissolved. To the stirring mixture was then added DMAP (0.1 eq.) and Pfp–ester (2, or 22–24, 5.0 eq.). The mixture was stirred until complete consumption of starting material (≥16 hours). The mixture was concentrated and redissolved in anhydrous MeOH, and NaOMe (10 eq., 0.03 M) added. The mixture was stirred overnight, and then neutralized (pH 7–8) with pre-washed Dowex 50 × 8 H^+^-Form, filtered and concentrated. Column chromatography, using DCM/MeOH gradients, afforded the 3, and 25–27, respectively.

### General procedure 4 (GP3): copper-catalyzed azide–alkyne cycloaddition (CuAAC)

An oven-dried round bottom flask equipped with magnetic stirring bar was charged with azido-sialoside (0.37 mmol, 11.5 eq.). To this was added a solution of pentapropargylated glucoside (1.0 eq., 0.032 mmol) in tetrahydrofuran (THF, 7 mL). To the stirring solution was added CuSO_4_·5H_2_O (1.59 eq.) and sodium ascorbate (1.55 eq.) in H_2_O (7 mL). The flask was equipped with rubber septum and the mixture heated to 50 °C for 5 h and then the reaction was left to perform at room temp for 36 h. The THF was removed under reduced pressure and the resulting mixture was purified by HPLC (MeCN/H_2_O 10% → 25% gradient in 25 minutes) affording the pentavalent methyl ester derivative after freeze-drying. See the ESI† for specific yields and analytical data.

### General procedure 3 (GP4): microwave CuAAC

The general procedure exemplified with synthesis of compound 13. An microwave vial equipped with magnetic stirring bar was charged with azido-sialoside 7 (0.08 mmol, 1.0 eq.), the C9 alkynyl derivative 3 (0.08 mmol, 1.0 eq.), CuSO_4_·5H_2_O (0.3 eq.) and sodium ascorbate (0.3 eq.). This was diluted with a 1 : 1 mixture of THF and water (0.7 mL). The microwave vial was capped and the mixture irradiated at 100 °C for 2 h. The THF was removed under reduced pressure and the resulting mixture was purified by HPLC (MeCN/H_2_O 10% → 25% gradient in 25 min) affording the pentavalent methyl ester derivative after freeze-drying.

### General procedure 5 (GP5): ester hydrolysis

The pentavalent methyl ester derivative (0.01 mmol, 1.0 eq.) was dissolved in MeOH (1.35 mL) and to this stirring solution was added a 1 M solution of LiOH (0.156 mL, 15.0 eq.). The mixture was stirred for 48 h at room temperature in the dark. The mixture was neutralized (pH 7–8) with Dowex 50 × 8 H-form, filtered, and concentrated under reduced pressure. The resulting residue was diluted in water and freeze-dried to afford the pentavalent target compound.

### Methyl (methyl 5-acetamido-9-(pent-4-ynamido)-3,5,9-trideoxy-d-*glycero*-α-d-*galacto*-2-nonulopyranosid)onate (3)

Synthesized according to GP2. ^1^H-NMR (600 MHz, CD_3_OD): *δ* 1.71 (dd, *J* = 12.8 Hz, 11.9 Hz, 1H), 2.00 (s, 3H), 2.26 (t, *J* = 2.6 Hz, 1H), 2.41–2.45 (m, 2H), 2.45–2.50 (m, 2H), 2.64 (dd, *J* = 12.8 Hz, 4.7 Hz, 1H), 3.27–3,32 (overlapped with solvent, 1H), 3.33 (s, 3H), 3.38 (dd, *J* = 8.8 Hz, 1.4 Hz, 1H), 3.59–3.66 (overlapped signals, 3H), 3.76 (appear as t, *J* = 10.2 Hz, 1H), 3.83 (s, 3H), 3.9 (ddd, *J* = 9.2 Hz, 7.3 Hz, 3.0 Hz, 1H). ^13^C-NMR (150 MHz, CD_3_OD): *δ* 15.76, 22.75, 36.05, 41.42, 44.15, 52.02, 53.27, 53.84, 68.57, 70.29, 70.88, 71.72, 74.62, 83.60, 100.35, 170.78, 174.50, 175.03. Column chromatography with DCM/MeOH gradient. *R*_f_ = 0.11 (5% MeOH in DCM, 0.1% formic acid). The compound was isolated as a slightly yellowish foam in 50% yield. Molecular formula: C_18_H_28_N_2_O_9_. HRMS *m*/*z*: calculated for [M + Na]^+^ 439.1687; found 439.1706.

#### Compound 10

Synthesized according to GP4. ^1^H-NMR (600 MHz, CD_3_OD): *δ* 1.48–1.59 (m, 2H), 1.71 (t, *J* = 12.3 Hz, 1H), 1.73 (t, *J* = 12.3 Hz, 1H), 1.73 (t, *J* = 12.3 Hz, 1H), 1.89–1.98 (m, 2H), 1.99 (s, 3H), 2.0 (s, 3H), 2.59 (t, *J* = 7.2 Hz, 2H), 2.64 (dd, *J* = 10.7 Hz, 4.4 Hz, 1H), 2.67 (dd, *J* = 10.7 Hz, 4.7 Hz, 1H), 3.0 (t, *J* = 7.5 Hz, 2H), 3.27 (dd, *J* = 13.9 Hz, 7.8 Hz, 1H), 3.33 (s, 3H), 3.37 (dd, *J* = 9.0 Hz, 1.2 Hz, 1H), 3.41 (dt, *J* = 9.4 Hz, 6.1 Hz, 1H), 3.51 (dd, *J* = 8.6 Hz, 1.5 Hz, 1H), 3.58 (dd, *J* = 10.5 Hz, 1.5 Hz, 1H), 3.60–3.70 (overlapped signals, 5H), 3.74–3.80 (overlapped signals, 2H), 3.80–3.93 (overlapped signals, 4H), 3.83 (s, 3H), 3.84 (s, 3H), 4.39 (t, *J* = 7.0 Hz, 2H), 7.78 (s, 1H). ^13^C-NMR (150 MHz, CD_3_OD): *δ* 22.60, 22.71, 22.79, 36.38, 41.37, 41.66, 44.15, 50.88, 52.07, 53.39, 53.46, 53.79, 64.36, 64.73, 68.47, 68.54, 70.20, 70.82, 71.81, 72.48, 74.59, 74.90, 100.12, 100.32, 123.54, 147.68, 170.78, 171.05, 175.01, 175.19. 72% yield. Molecular formula: C_34_H_56_N_6_O_18_. HRMS *m*/*z*: calculated for [M + H]^+^ 837.3724; found 837.3714.

#### Compound 11

Synthesized according to GP4. ^1^H-NMR (600 MHz, CD_3_OD): *δ* 1.71 (t, *J* = 12.3 Hz, 1H), 1.74 (t, *J* = 12.0 HZ, 1H), 1.99 (d, 3H), 2.0 (s, 3H), 2.6 (t, *J* = 7.4 HZ, 2H), 2.64 (dd, *J* = 12.7 HZ, 4.4 HZ, 1H), 2.68 (dd, *J* = 12.9 Hz, 4.8 Hz, 1H), 3.01 (t, *J* = 7.2 Hz, 2H), 3.27 (dd, *J* = 13.5 Hz, 7.6 Hz, 1H), 3.33 (s, 3H), 3.35–3.39 (overlapped with solvent, 2H), 3.5 (d, *J* = 8.8 Hz, 1H), 3.53–3.70 (m, 13H), 3.73–3.80 (m, 2H), 3.81–3.91 (overlapped, 5H), 3.82 (s, 3H), 3.84 (s, 3H), 4.54 (t, *J* = 4.8 Hz, 2H), 7.82 (s, 1H). ^13^C-NMR (150 MHz, CD_3_OD): *δ* 22.63, 22.73, 22.80, 36.42, 41.37, 41.60, 44.18, 49.85, 51.38, 52.08, 53.37, 53.47, 53.76, 53.80, 64.67, 64.77, 68.52, 68.55, 70.23, 70.44, 70.82, 71.16, 71.44, 71.52, 71.82, 72.47, 74.60, 74.90, 100.17, 100.34, 124.34, 147.62, 170.78, 170.85, 175.01, 175.03, 175.12, 175.20. 55% yield. Molecular formula: C_36_H_60_N_6_O_20_. HRMS *m*/*z*: calculated for [M + Na]^+^ 919.3755; found 919.3768.

#### Compound 12

Synthesized according to GP4. ^1^H-NMR (600 MHz, CD_3_OD): *δ* 1.61–1.68 (m, 2H), 1.73 (appear as q, *J* = 12.9 Hz, 2H), 1.99 (s, 3H), 2.01 (s, 3H), 2.58 (t, *J* = 7.3 Hz, 2H), 2.65 (appear as ddd, *J* = 13.2 Hz, 7.4 Hz, 4.7 Hz, 2H), 2.76–2.86 (m, 2H), 2.99 (appear as t, *J* = 7.4 Hz, 2H), 3.21 (t, *J* = 6.4 Hz, 2H), 3.27 (dd, 13.8 Hz, 7.5 Hz, 1H), 3.35–3.41 (overlapped signal, 2H), 3.33 (s, 3H), 3.46–3.54 (overlapped with solvent, 1H), 3.55–3.71 (overlapped signals, 6H), 3.73–3.79 (overlapped signals, 3H), 3.81–3.91 (overlapped signals, 3H), 3.82 (s, 3H), 3.84 (s, 3H), 4.63 (t, *J* = 6.5 Hz, 2H), 7.72 (s, 1H). ^13^C-NMR (150 MHz, CD_3_OD): *δ* 22.59, 22.73, 22.79, 30.29, 36.34, 37.13, 37.33, 41.37, 41.43, 44.15, 47.52, 49.85, 52.08, 53.35, 53.43, 53.81, 62.43, 64.80, 68.55, 70.17, 70.83, 71.82, 72.40, 74.61, 74.77, 100.10, 100.35, 123.90, 147.60, 170.78, 170.98, 172.01, 175.01, 175.16. 62% yield. Molecular formula: C_36_H_59_N_7_O_19_. HRMS ESI *m*/*z*: calculated for [M + H]^+^ 894.3939; found 894.3933.

#### Compound 13

Synthesized according to GP4. ^1^H-NMR (600 MHz, CD_3_OD): *δ* 1.25–1.36 (m, 2H), 1.41–1.49 (m, 2H), 1.52 (m, *J* = 7.0 Hz, 2H), 1.71 (dd, *J* = 12.4 Hz, 6.9 Hz, 1H), 1.73 (dd, *J* = 12.2 Hz, 7.1 Hz, 1H), 1.99 (s, 3H), 2.0 (s, 3H), 2.57 (t, *J* = 7.4 Hz, 2H), 2.64 (dd, *J* = 12.6 Hz, 4.2 Hz, 1H), 2.67 (dd, *J* = 16.9 Hz, 4.5 Hz, 1H), 2.79 (t, *J* = 6.8 Hz, 2H), 2.99 (t, *J* = 7.4 Hz, 2H), 3.13 (t, *J* = 7.0 Hz, 2H), 3.27 (dd, *J* = 13.7 Hz, 7.4 Hz, 1.0 Hz), 3.35–3.38 (overlapped, 2H), 3.32 (s, 3H), 3.51 (appear as d, *J* = 9.4 Hz, 1H), 3.56 (dd, *J* = 10.4 Hz, 2.1 Hz, 1H), 3.60–3.68 (m, 5H), 3.73–3.80 (overlapped signal, 3H), 3.82–3.91 (overlapped, 3H), 3.83 (s, 3H), 3.84 (s, 3H), 4.63 (t, *J* = 6.7 Hz, 2H), 7.71 (s, 3H). ^13^C-NMR (150 MHz, CD_3_OD): *δ* 22.58, 22.70, 22.78, 24.26, 29.84, 30.23, 36.35, 37.11, 40.30, 41.38, 41.74, 44.15, 47.50, 49.85, 52.07, 53.35, 53.41, 53.81, 53.84, 64.74, 64.91, 68.53, 68.56, 70.25, 70.83, 71.81, 72.58, 74.61, 74.89, 100.16, 100.35, 123.89, 147.57, 170.79, 171.18, 171.83, 174.99, 175.01, 175.21. 59% yield. Molecular formula: C_38_H_63_N_7_O_19_. HRMS *m*/*z*: calculated for [M + H]^+^ 922.4252; found 922.4264.

#### Compound 14

Synthesized according to GP4. ^1^H-NMR (600 MHz, CD_3_OD): *δ* 1.30–1.42 (m, 2H), 1.45–1.52 (m, 2H), 1.52–1.59 (m, 2H), 1.73 (t, *J* = 12.4 Hz, 1H), 1.74 (t, *J* = 12.4 Hz, 1H), 1.99 (s, 3H), 2.0 (S, 3H), 2.13–2.24 (m, 4H), 2.59 (t, *J* = 7.4 Hz, 2H), 2.64 (dd, *J* = 13.2 Hz, 4.4 Hz, 1H), 2.67 (dd, *J* = 13.8 Hz, 4.7 Hz, 1H), 3.0 (t, *J* = 7.4 Hz, 2H), 3.12–3.30 (m, 2H), 3.27 (dd, *J* = 13.9 Hz, 7.6 Hz, 1H), 3.33 (s, 3H), 3.36–3.39 (overlapped with solvent 2H), 3.51 (appear as d, *J* = 9.0 Hz, 1H), 3.56 (d, *J* = 10.5 Hz, 1H), 3.60–3.68 (overlapped signals, 5H), 3.73–3.81 (overlapped signals, 3H), 3.81–3.91 (overlapped signals, 3H), 3.83 (s, 3H), 3.84 (s, 3H), 4.4 (t, *J* = 6.3 Hz, 2H), 7.78 (s, 1H). ^13^C-NMR (150 MHz, CD_3_OD): *δ* 22.62, 22.71, 22.78, 24.33, 27.44, 29.90, 30.25, 33.51, 36.35, 40.28, 41.37, 41.75, 44.14, 49.85, 50.61, 52.06, 53.36, 53.40, 53.80, 53.84, 64.71, 64.90, 68.50, 68.54, 70.23, 70.82, 71.81, 74.61, 74.88, 100.15, 100.34, 123.63, 147.76, 170.78, 171.18, 174.31, 175.02, 175.21. 64% yield. Molecular formula: C_39_H_65_N_7_O_19_. HRMS *m*/*z*: calculated for [M + H]^+^ 936.4408; found 936.4417.

#### Compound 15

Synthesized according to GP4. ^1^H-NMR (600 MHz, CD_3_OD): *δ* 1.30–1.42 (m, 2H), 1.46–1.52 (m, 2H), 1.52–1.57 (m, 2H), 1.57–1.63 (m, 2H), 1.71 (t, *J* = 12.3 Hz, 1H), 1.72 (t, *J* = 12.4 Hz, 1H), 1.86–1.94 (m, 2H), 1.99 (s, 3H), 2.0 (s, 3H), 2.22 (t, *J* = 7.4 Hz, 2H), 2.58 (t, *J* = 7.5 Hz, 2H), 2.64 (dd, *J* = 12.7 Hz, 4.6 Hz, 1H), 2.67 (dd, *J* = 12.9 Hz, 4.6 Hz, 1H), 3.0 (t, *J* = 7.3 Hz, 2H), 3.13–3.18 (m, 2H), 3.27 (dd, *J* = 14.0 Hz, 7.2 Hz, 1H), 3.33 (s, 3H), 3.36–3.39 (overlapped with solvent, 2H), 3.51 (appear as d, *J* = 9.0 Hz, 1H), 3.56 (appear as d, *J* = 10.5 Hz, 1H), 3.60–3.68 (overlapped signals, 5H), 3.73–3.80 (overlapped signals, 3H), 3.81–3.01)overlapped signals, 3H), 3.83 (s, 3H), 3.84 (s, 3H), 4.38 (t, *J* = 7.0 Hz, 2H), 7.77 (s, 1H). ^13^C-NMR (150 MHz, CD_3_OD): *δ* 22.62, 22.71, 22.78, 23.86, 24.34, 29.94, 30.26, 30.74, 36.23, 36.39, 40.24, 41.39, 41.77, 44.17, 49.85, 50.90, 52.06, 53.36, 53.41, 53.81, 53.85, 64.72, 64.90, 68.50, 68.54, 70.24, 70.82, 71.84, 72.58, 74.62, 74.89, 100.15, 100.34, 123.53, 147.71, 170.78, 171.19, 175.02, 175.22, 175.29. Molecular formula: C_39_H_65_N_7_O_19_. HRMS *m*/*z*: calculated for [M + H]^+^ 950.4565; found 950.4571.

#### Compound 16

Synthesized according to GP5. ^1^H-NMR (600 MHz, D_2_O): *δ* 1.46–1.57 (m, 2H), 1.63 (t, *J* = 12.2 Hz, 1H), 1.64 (t, *J* = 12.1 Hz, 1H), 1.92–1.98 (m, 2H), 2.03 (s, 3H), 2.04 (s, 3H), 2.64 (t, *J* = 7.5 Hz, 2H), 2.71 (dd, *J* = 7.5 Hz, 4.7 Hz, 1H), 2.73 (dd, *J* = 11.9 Hz, 4.6 Hz, 1H), 3.01 (t, *J* = 7.4 Hz, 2H), 3.26 (dd, *J* = 14.1 Hz, 7.7 Hz, 1H), 3.83 (s, 3H), 3.42–3.49 (overlapped signals, 2H), 3.54–3.61 (overlapped signals, 2H), 3.61–3.71 (overlapped signals, 4H), 3.70–3.76 (overlapped signals, 2H), 3.81 (t, *J* = 10.2 Hz, 2H), 3.82–3.92 (overlapped signals, 3H), 4.41 (t, *J* = 7.0 Hz, 2H), 7.80 (s, 1H). ^13^C-NMR (150 MHz, D_2_O): *δ* 21.17, 22.0, 22.02, 25.88, 26.23, 35.14, 40.12, 40.36, 42.00, 49.94, 51.56, 51.88, 51.90, 62.56, 63.92, 68.17, 68.23, 68.28, 69.66, 69.89, 71.74, 72.41, 72.57, 100.61, 100.70, 123.20, 146.39, 173.39, 173.60, 175.05, 175.32. 73% yield. Molecular formula: C_32_H_52_N_6_O_18_. HRMS *m*/*z*: calculated for [M + H]^+^ 809.3411; found 809.3433.

#### Compound 17

Synthesized according to GP5. ^1^H-NMR (600 MHz, D_2_O): *δ* 1.63 (t, *J* = 12.1 Hz, 1H), 1.67 (t, *J* = 12.1 Hz, 1H), 2.03 (s, 3H), 2.04 (s, 1H), 2.66 (t, *J* = 7.4 Hz, 2H), 2.71 (dd, *J* = 12.2 Hz, 4.5 Hz, 1H), 2.74 (dd, *J* = 12.3 Hz, 4.5 Hz, 1H), 3.03 (t, *J* = 7.5 Hz, 2H), 3.25 (dd, *J* = 14.1 Hz, 7.8 Hz, 1H), 3.33 (s, 3H), 3.46 (dd, *J* = 9.0 Hz, 1.7 Hz, 1H), 3.54–3.74 (overlapped signals, 14H), 3.81 (td, *J* = 10.1 Hz, 8.4 Hz, 2H), 3.85–3.89 (m, 4H), 3.96 (t, *J* = 5.1 Hz, 2H), 4.59 (t, *J* = 5.0 Hz, 2H), 7.85 (s, 1H). ^13^C-NMR (150 MHz, D_2_O): *δ* 21.14, 22.01, 35.11, 40.11, 40.23, 42.02, 49.90, 51.56, 51.87, 51.90, 62.61, 63.21, 68.21, 68.24, 68.84, 69.38, 69.56, 69.60, 69.69, 71.72, 72.41, 72.56, 100.49, 100.69, 123.65, 164.42, 173.40, 174.98, 175.05, 175.28. Molecular formula: C_34_H_56_N_6_O_20_. HRMS *m*/*z*: calculated for [M + H]^+^ 869.3622; found 869.3468. 93% yield.

#### Compound 18

Synthesized according to GP5. ^1^H-NMR (600 MHz, D_2_O): *δ* 1.58–1.70 (m, 4H), 2.03 (2, 3H), 2.04 (s, 3H), 2.64 (t, *J* = 7.4 Hz, 2H), 2.7 (dd, *J* = 4.5 Hz, 2.9 Hz, 1H), 2.72 (dd, *J* = 4.5 Hz, 3.0 Hz, 1H), 2.83 (t, *J* = 6.5 Hz, 2H), 3.01 (t, *J* = 7.4 Hz, 2H), 3.11–3.23 (overlapped signals, 2H), 3.26 (dd, 13.9 Hz, 7.4 Hz, 1H), 3.4 (dt, *J* = 10.0 Hz, 6.4 Hz, 1H), 3.45 (dd, *J* = 8.9 Hz, 1.6 Hz, 1H), 3.57 (dd, *J* = 13.8 Hz, 2.8 Hz, 1H), 3.58 (dd, *J* = 8.6 Hz, 1.4 Hz, 1H), 3.61–3.70 (overlapped signals, 5H), 3.72 (dd, *J* = 10.5 Hz, 1.9 Hz, 1H), 3.81 (t, *J* = 1.03 Hz, 2H), 3.83–3.92 (overlapped signals, 3H), 4.66 (t, *J* = 6.6 Hz, 2H), 7.75 (s, 1H). ^13^C-NMR (150 MHz, D_2_O): *δ* 21.08, 22.01, 22.03, 23.25, 28.50, 35.06, 36.31, 36.36, 40.11, 40.28, 42.01, 46.62, 51.57, 51.90, 61.97, 62.60, 68.20, 68.24, 68.27, 69.69, 69.88, 71.76, 72.42, 72.55, 100.70, 123.32, 146.38, 146.38, 172.10, 173.40, 174.98, 175.05, 175.25. 92% yield. Molecular formula: C_34_H_55_N_7_O_19_. HRMS *m*/*z*: calculated for [M + H]^+^ 866.3626; found 866.3645.

#### Compound 19

Synthesized according to GP5. ^1^H-NMR (600 MHz, D_2_O): *δ* 1.15–1.24 (m, 2H), 1.37 (dq, *J* = 8.7 Hz, 7.2 Hz, 2H), 1.52 (m, *J* = 7.4 Hz, 2H), 1.63 (t, *J* = 12.2 Hz, 1H), 1.63 (t, *J* = 12.1 Hz, 1H), 2.03 (s, 3H), 2.04 (s, 3H), 2.64 (t, *J* = 7.3 Hz, 2H), 2.71 (dd, *J* = 12.1 Hz, 43. Hz, 1H), 2.74 (dd, *J* = 12.1 Hz, 4.3 Hz, 1H), 2.82 (t, *J* = 6.4 Hz, 2H), 3.0 (t, *J* = 7.3 Hz, 2H), 3.09 (t, *J* = 6.8 Hz, 2H), 3.23 (dd, *J* = 14.1 Hz, 7.9 Hz, 1H), 3.33 (s, 3H), 3.40–3.44 (m, 1H), 3.45 (dd, *J* = 8.9 Hz, 1.5 Hz, 1H), 3.54–3.61 (overlapped signals, 2H), 3.62–3.65 (overlapped signals, 6H), 3.81 (td, *J* = 10.1 Hz, 3.5 Hz, 2H), 3.84–3.91 (overlapped signals, 3H), 4.66 (t, *J* = 6.3 Hz, 2H), 7.75 (s, 1H). ^13^C-NMR (150 MHz, D_2_O): *δ* 21.07, 22.01, 22.03, 22.32, 23.25, 27.78, 28.49, 35.04, 36.34, 39.13, 40.11, 40.48, 42.03, 46.69, 51.57, 51.91, 62.58, 64.61, 68.23, 68.30, 69.71, 69.88, 71.79, 72.42, 72.56, 100.64, 100.70, 123.32, 146.36, 171.99, 173.40, 173.62, 174.99, 175.06, 175.21. 86% yield. Molecular formula: C_36_H_59_N_7_O_19_. HRMS *m*/*z*: calculated for [M + H]^+^ 894.3939; found 894.3953.

#### Compound 20

Synthesized according to GP5. ^1^H-NMR (600 MHz, D_2_O): *δ* 1.27–1.37 (m, 2H), 1.41–1.50 (m, 2H), 1.51–1.58 (m, 2H), 1.61 (t, *J* = 12.1 Hz, 2H), 2.01 (s, 3H), 2.02 (s, 3H), 2.12–2.25 (m, 4H), 2.63 (t, *J* = 6.7 Hz, 2H), 2.7 (appear as dddd, *J* = 14.7 Hz, 12.5 Hz, 5.0 Hz, 2.3 Hz, 2H), 3.0 (t, *J* = 7.0 Hz, 2H), 3.09 (t, *J* = 6.3 Hz, 2H), 3.22 (dd, *J* = 13.9 Hz, 7.7 Hz, 1H), 3.31 (s, 3H), 3.39–3.47 (m, 2H), 3.51–3.60 (m, 2H), 3.60–3.74 (m, 6H), 3.74–3.80 (m, 2H), 3.81–3.91 (m, 3H), 4.39 (t, *J* = 5.8 Hz, 2H), 7.77 (s, 1H). ^13^C-NMR (150 MHz, D_2_O): *δ* 21.14, 22.02, 22.52, 23.25, 25.61, 27.84, 28.55, 32.49, 35.09, 39.25, 40.25, 40.59, 42.03, 49.56, 51.54, 52.03, 62.57, 64.65, 68.20, 68.27, 69.74, 69.89, 71.78, 72.46, 72.59, 100.71, 100.65, 123.25, 146.43, 164.43, 173.41, 173.67, 174.71, 175.02, 176.08, 175.25. Quantitative yield. Molecular formula: C_37_H_61_N_7_O_19_. HRMS *m*/*z*: calculated for [M + H]^+^ 908.4095; found 908.4106.

#### Compound 21

Synthesized according to GP5. ^1^H-NMR (600 MHz, D_2_O): *δ* 1.31–1.37 (m, 2H), 1.46–1.60 (m, 6H), 1.63 (t, *J* = 12.2 Hz, 1H), 1.64 (t, *J* = 12.2 Hz, 1H), 1.88 (m, *J* = 7.5 Hz, 2H), 2.03 (s, 3H), 2.04 (s, 3H), 2.25 (t, *J* = 7.3 Hz, 2H), 2.64 (t, *J* = 7.3 Hz, 2H), 2.71 (dd, *J* = 12.7 Hz, 4.8 Hz, 1H), 2.74 (dd, *J* = 12.5 Hz, 4.7 Hz, 1H), 3.02 (t, *J* = 7.3 Hz, 2H), 3.16 (t, *J* = 6.9 Hz, 2H), 3.24 (dd, *J* = 14.1 Hz, 7.7 Hz, 1H), 3.33 (s, 3H), 3.42–3.48 (m, 2H), 3.56 (dd, *J* = 14.1 Hz, 2.7 Hz, 1H), 3.59 (dd, *J* = 8.8 Hz, 1.8 Hz, 1H), 3.62–3.76 (overlapped signals, 6H), 3.81 (td, *J* = 10.1 Hz, 4.8 Hz, 2H), 3.84–3.91 (m, 3H), 4.4 (t, *J* = 6.9 Hz, 2H), 7.79 (s, 1H). ^13^C-NMR (150 MHz, D_2_O): *δ* 21.15, 22.00, 22.26, 22.51, 23.24, 27.89, 28.54, 28.76, 34.99, 35.14, 39.17, 40.11, 40.48, 42.02, 49.85, 51.56, 51.90, 62.56, 64.64, 68.19, 68.22, 68.29, 69.71, 69.89, 71.70, 72.42, 75.56, 100.69, 100.64, 123.18, 146.41, 173.38, 173.63, 174.99, 175.06, 175.28, 175.97. Quantitative yield. Molecular formula: C_38_H_63_N_7_O_19_. HRMS *m*/*z*: calculated for [M + H]^+^ 922.4252; found 922.4272.

### Methyl (methyl 5-acetamido-9-(3-azidopropanamido)-3,5,9-trideoxy-d-*glycero*-α-d-*galacto*-2-nonulopyranosid)onate (25)

Synthesized according to GP1. ^1^H-NMR (400 MHz, CD_3_OD): *δ* 1.74 (dd, *J* = 12.8 Hz, 11.8 Hz, 1H), 2.02 (s, 3H), 2.52 (t, *J* = 6.4 Hz, 2H), 2.68 (dd, *J* = 12.7 Hz, 5.0 Hz, 1H), 3.28–3.37 (overlapped with solvent, 1H), 3.36 (s, 3H), 3.40 (dd, *J* = 9.0 Hz, 1.0 Hz, 1H), 3.52–3.61 (m, 2H), 3.62–3.71 (m, 3H), 3.79 (t, *J* = 10.0 Hz, 1H), 3.86 (s, 3H), 3.91–3.97 (m, 1H). ^13^C-NMR (100 MHz, CD_3_OD): *δ* 22.72, 36.22, 41.42, 44.18, 52.03, 53.30, 53.81, 68.55, 70.82, 71.74, 74.61, 100.33, 180.78, 173.38, 175.04. 62% yield. Molecular formula: C_16_H_27_N_5_O_9_. HRMS *m*/*z*: calculated for [M + Na]^+^ 456.1701; found 456.1708.

### Methyl (methyl 5-acetamido-9-(5-azidopetanamido)-3,5,9-trideoxy-d-*glycero*-α-d-*galacto*-2-nonulopyranosid)onate (26)

Synthesized according to GP1. ^1^H-NMR (400 MHz, CD_3_OD): *δ* 1.57–1.78 (m, 5H), 2.01 (s, 3H), 2.28 (t, *J* = 7.3 Hz, 2H), 2.66 (dd, *J* = 12.8 Hz, 4.6 Hz, 1H), 3.26–3.41 (overlapped with solvent, m, 4H), 3.35 (s, 3H), 3.61–3.70 (m, 3H), 3.78 (appear as t, *J* = 10.2 Hz, 1H), 3.85, (s, 3H), 3.91 (ddd, *J* = 8.7 Hz, 7.3 Hz, 3.1 Hz, 1H). ^13^C-NMR (100 MHz, CD_3_OD): *δ* 22.73, 24.18, 29.42, 36.37, 41.42, 44.14, 52.02, 52.14, 53.29, 53.82, 68.55, 70.89, 71.81, 74.61, 100.34, 170.78, 175.00, 176.20. 55% yield. Molecular formula: C_18_H_31_N_5_O_9_. HRMS *m*/*z*: calculated for [M + H]^+^ 462.2195; found 462.2200.

### Methyl (methyl 5-acetamido-9-(9-azidononanamido)-3,5,9-trideoxy-d-*glycero*-α-d-*galacto*-2-nonulopyranosid)onate (27)

Synthesized according to GP1. ^1^H-NMR (400 MHz, CD_3_OD): *δ* 1.29–1.45 (m, 8H), 1.55–1.68 (m, 4H), 1.73 (appear as t, *J* = 12.3 Hz, 1H), 2.0 (s, 3H), 2.24 (t, *J* = 7.6 Hz, 2H), 2.66 (dd, *J* = 12.8 Hz, 4.7 Hz, 1H), 3.25–3.34 (overlapped with solvent, 1H), 3.29 (t, *J* = 6.9 Hz, 2H), 3.35 (s, 3H), 3.34–3.41 (overlapped signal, m, 1H), 3.60–3.70 (m, 3H), 3.78 (t, *J* = 10.3 Hz, 1H), 3.85 (s, 3H), 3.91 (td, *J* = 8.2 Hz, 3.0 Hz, 1H). ^13^C-NMR (100 MHz, CD_3_OD): *δ* 22.73, 27.04, 27.78, 29.90, 30.12, 30.21, 30.31, 37.07, 41.42, 44.16, 52.02, 52.45, 53.29, 53.84, 68.58, 70.94, 71.82, 74.61, 100.36, 170.80, 175.00, 176.87. 29% yield. Molecular formula: C_22_H_39_N_5_O_9_. HRMS *m*/*z*: calculated for [M + Na]^+^ 540.2640; found 540.2654.

#### Compound 29

Synthesized according to GP3. ^1^H-NMR (600 MHz, CD_3_OD): *δ* 1.53–1.64 (m, 3H), 1.66–1.78 (m, 3H), 1.94 (s, 3H), 2.00 (s, 3H), 2.64 (dd, *J* = 12.7 Hz, 4.3 Hz, 1H), 2.69 (t, *J* = 7.5 Hz, 2H), 2.77 (dd, *J* = 12.9 Hz, 4.4 Hz, 1H), 2.85 (dd, *J* = 7.1 Hz, 6.5 Hz, 2H), 3.27 (dd, *J* = 13.9 Hz, 7.3 Hz, 1H), 3.33 (s, 3H), 3.33–3.37 (overlapped with solvent, 1H), 3.35 (s, 3H), 3.39–3.47 (m, 2H), 3.51 (dd, *J* = 8.8 Hz, 1.8 Hz, 1H), 3.56–3.68 (m, 4H), 3.61 (t, *J* = 10.9 Hz, 2H), 3.76 (t, *J* = 9.9 Hz, 1H), 3.80–3.91 (m, 4H), 3.84 (s, 3H), 3.85 (s, 3H), 4.65 (t, *J* = 6.7 Hz, 2H), 7.71 (s, 1H). ^13^C-NMR (150 MHz, CD_3_OD): *δ* 22.76, 22.79, 25.90, 27.13, 30.30, 37.08, 38.48, 41.41, 44.12, 47.43, 52.04, 52.07, 53.37, 53.80, 64.73, 68.51, 69.87, 70.22, 70.77, 71.80, 72.30, 74.60, 74.73, 76.39, 100.33, 100.39, 123.66, 148.95, 170.72, 170.76, 172.52, 174.68, 175.09. 78% yield. Molecular formula: C_35_H_58_N_6_O_18_. HRMS *m*/*z*: calculated for [M + H]^+^ 851.3880; found 851.3901.

#### Compound 30

Synthesized according to GP3. ^1^H-NMR (600 MHz, CD_3_OD): *δ* 1.45–1.53 (m, 5H), 1.68–1.77 (m, 3H), 1.87–1.96 (m, 2H), 1.93 (s, 3H), 1.99, (s, 3H), 2.27 (t, *J* = 7.4 Hz, 2H), 2.64 (dd, *J* = 12.3 Hz, 4.7 Hz, 1H), 2.71 (dd, *J* = 8.1 Hz, 6.8 Hz, 2H), 2.77 (dd, *J* = 12.5 Hz, 4.7 Hz, 1H), 3.28 (dd, *J* = 14.2 Hz, 7.4 Hz, 1H), 3.32 (s, 3H), 3.33–3.38 (overlapped with solvent, 1H), 3.35 (s, 3H), 3.39–3.47 (m, 2H), 3.51 (dd, *J* = 9.1 Hz, 1.5 Hz, 1H), 3.59–3.68 (m, 6H), 3.76 (t, *J* = 10.4 Hz, 1H), 3.81–3.87 (m, 3H), 3.83 (s, 3H), 3.85 (s, 3H), 3.89 (td, *J* = 8.2 Hz, 3.3 Hz, 1H), 4.38 (t, *J* = 7.0 Hz, 2H), 7.75 (s, 1H). ^13^C-NMR (150 MHz, CD_3_OD): *δ* 22.73, 22.76, 23.78, 25.94, 21.17, 30.35, 30.73, 36.10, 38.47, 41.41, 44.16, 50.87, 52.02, 52.03, 52.06, 53.32, 53.37, 53.81, 64.75, 68.54, 69.85, 70.17, 70.85, 71.83, 72.36, 74.62, 74.75, 76.42, 100.33, 100.37, 123.29, 149.07, 170.75, 170.77, 174.65, 174.99, 175.90. 67% yield. Molecular formula: C_37_H_62_N_6_O_18_. HRMS *m*/*z*: calculated for [M + H]^+^ 879.4193; found 879.5211.

#### Compound 31

Synthesized according to GP3. ^1^H-NMR (600 MHz, CD_3_OD): *δ* 1.24–1.40 (m, 8H), 1.53–1.65 (m, 5H), 1.68–1.76 (m, 3H), 1.84–1.94 (m, 2H), 1.93 (s, 3H), 1.99 (s, 3H), 2.21 (dd, *J* = 8.1 Hz, 6.9 Hz, 2H), 2.64 (dd, *J* = 12.8 Hz, 4.1 Hz, 1H), 2.71 (d, *J* = 7.8 Hz, 2H), 2.77 (dd, *J* = 13.2 Hz, 5.0 Hz, 1H), 3.27 (dd, *J* = 14.1 Hz, 7.4 Hz, 1H), 3.32–3.37 (m, 1H), 3.33 (s, 3H), 3.35 (s, 3H), 3.38–3.48 (m, 2H), 3.51 (dd, *J* = 9.0 Hz, 1.9 Hz, 1H), 3.58–3.69 (m, 6H), 3.76 (t, *J* = 10.3 Hz, 1H), 3.81–3.87 (m, 3H), 3.83 (s, 3H), 3.85 (s, 3H), 3.89 (td, *J* = 8.0 Hz 3.0 Hz, 1H), 4.36 (t, *J* = 7.2 Hz, 2H), 7.73 (s, 1H). ^13^C NMR (150 MHz, CD_3_OD): *δ* 22.73, 22.76, 25.93, 26.98, 27.22, 27.41, 29.87, 30.12, 30.17, 30.36, 31.26, 37.03, 38.48, 41.42, 44.16, 51.23, 52.03, 52.07, 53.31, 53.36, 53.83, 64.76, 68.56, 69.86, 70.18, 70.93, 71.81, 70.93, 71.81, 72.36, 74.61, 74.77, 76.45, 100.34, 100.39, 123.18, 148.97, 170.76, 170.78, 174.63, 174.97, 176.80. 35% yield. Molecular formula: C_41_H_70_N_6_O_18_. HRMS *m*/*z*: calculated for [M + H]^+^ 935.4839; found 935.4839.

#### Compound 32

Synthesized according to GP5. ^1^H-NMR (600 MHz, D_2_O): *δ* 1.48–1.57 (m, 3H), 1.57–1.70 (m, 3H), 1.91 (s, 3H), 2.01 (s, 3H), 2.68 (t, *J* = 7.5 Hz, 2H), 2.69 (dd, *J* = 12.2 Hz, 5.0 Hz, 1H), 2.83 (dd, *J* = 12.4 Hz, 4.7 Hz, 1H), 2.87 (t, *J* = 6.5 Hz, 2H), 3.18 (dd, *J* = 14.0 Hz, 8.1 Hz, 1H), 3.31 (s, 3H), 3.33 (s, 3H), 3.40–3.49 (m, 3H), 3.54 (dd, *J* = 14.1 Hz, 2.6 Hz, 1H), 3.57 (dd, *J* = 99.3 Hz, 1.5 Hz, 1H), 3.60–3.68 (m, 1H), 3.62 (dd, *J* = 12.0 Hz, 6.2 Hz, 1H), 3.68–3.76 (m, 3H), 3.78 (t, *J* = 10.1 Hz, 1H), 3.80–3.89 (m, 4H), 4.66 (t, *J* = 6.5 Hz, 2H), 7.74 (s, 1H). ^13^C-NMR (150 MHz, D_2_O): *δ* 22.57, 22.68, 24.74, 25.89, 28.88, 36.67, 38.27, 40.76, 42.75, 47.06, 50.97, 52.23, 52.55, 63.26, 68.81, 68.87, 69.95, 70.38, 70.53, 72.27, 73.06, 73.27, 77.02, 101.34, 101.44, 123.82, 148.93, 173.17, 174.01, 175.19, 175.65. 85% yield. Molecular formula: C_33_H_54_N_6_O_18_. HR-MS (ESI-TOF) *m*/*z*: calculated for [M + H]^+^ 823.3567; found 823.3587.

#### Compound 33

Synthesized according to GP5. ^1^H-NMR (600 MHz, D_2_O): *δ* 1.48–1.58 (m, 5H), 1.58–1.71 (m 3H), 1.85–1.93 (m, 2H), 1.90 (s, 3H), 2.00 (s, 3H), 2.29 (t, *J* = 7.4 Hz, 2H), 2.66–2.73 (m, 3H), 2.84 (dd, *J* = 12.5 Hz, 4.6 Hz, 1H), 3.25–3.31 (m, 1H), 3.30 (s, 3H), 3.33 (s, 3H), 3.54–3.59 (m, 3H), 3.40–3.45 (m, 2H), 3.60–3.68 (m, 1H), 3.62 (dd, *J* = 12.5 Hz, 6.5 Hz, 1H), 3.69–3.77 (m, 1H), 3.71 (dd, *J* = 10.3 Hz, 1.4 Hz, 2H), 3.79 (t, *J* = 10.01 Hz, 1H), 3.82–3.92 (m, 3H), 3.90 (td, *J* = 8.4 Hz, 2.5 Hz, 1H), 4.25 (t, *J* = 6.9 Hz, 2H), 7.73 (s, 1H). ^13^C-NMR (150 MHz, D_2_O): *δ* 22.56, 22.66, 22.86, 24.79, 25.94, 28.92, 29.45, 35.54, 38.26, 40.78, 42.64, 50.43, 50.96, 52.19, 52.23, 52.56, 63.26, 68.81, 68.85, 69.95, 70.33, 70.52, 72.27, 73.08, 73.27, 77.05, 101.36, 101.44, 123.68, 149.01, 174.01, 174.06, 175.16, 175.60, 177.12. 92% yield. Molecular formula: C_35_H_58_N_6_O_18_. HRMS *m*/*z*: calculated for [M + H]^+^ 851.3880; found 851.3901.

#### Compound 34

Synthesized according to GP5. ^1^H-NMR (600 MHz, D_2_O): *δ* 1.22–1.31 (m, 8H), 1.48–1.59 (m, 5H), 1.60–1.72 (m, 3H), 1.83–1.89 (m, 2H), 1.90 (s, 3H), 2.02 (s, 3H), 2.25 (t, *J* = 7.5 Hz, 2H), 2.67–2.73 (m, 3H), 2.84 (dd, *J* = 12.5 Hz, 4.5 Hz, 1H), 3.27–3.32 (m, 1H), 3.30 (s, 3H), 3.33 (s, 3H), 3.42–3.48 (m, 2H), 3.63 (dd, *J* = 12.2 Hz, 6.7 Hz, 1H), 3.65–3.69 (m, 1H), 3.69–3.77 (m, 3H), 3.80 (t, *J* = 10.0 Hz, 1H), 3.82–3.93 (m, 4H), 4.37 (t, *J* = 6.9 Hz, 2H), 7.79 (s, 1H). ^13^C-NMR (150 MHz, D_2_O): *δ* 22.57, 22.68, 24.72, 25.95, 25.99, 26.01, 28.38, 28.59, 28.66, 28.89, 29.85, 36.34, 38.23, 40.77, 42.56, 50.95, 50.99, 52.21, 52.23, 52.57, 63.27, 68.82, 69.92, 70.24, 70.56, 72.24, 73.10, 73.29, 77.01, 101.31, 101.40, 123.81, 148.81, 173.95, 173.98, 175.15, 175.61, 178.23. 83% yield. Molecular formula: C_39_H_66_N_6_O_18_. HRMS *m*/*z*: calculated for [M + H]^+^ 907.4506; found 907.4531.

### 1,15-Bis((1-(methyl(5-*N*-acetamido-3,5-dideoxy-d-*glycero*-α-d-*galacto*-2-nonulopyranosyl)-onate)-2-1*H*-1,2,3-triazol-4-yl)-2,5,8,11,14-pentaoxapentadecane (38)

Synthesized according to GP3. ^1^H-NMR (600 MHz, CD_3_OD): *δ* 2.03 (s, 6H), 2.15 (dd, *J* = 12.9 Hz, 11.3 Hz, 2H), 3.27 (dd, *J* = 13.1 Hz, 4.7 Hz, 2H), 3.57 (dd, *J* = 8.7 Hz, 1.7 Hz, 2H), 3.60–3.71 (m, 18H), 3.78–3.86 (m, 4H), 3.83 (s, 6H), 3.88 (dd, *J* = 10.9 Hz, 1.9 Hz, 2H), 3.95 (t, *J* = 1.03 Hz, 2H), 4.04 (td, *J* = 10.4 Hz, 3.9 Hz, 2H), 4.64 (s, 4H), 8.34 (s, 2H). ^13^C-NMR (150 MHz, CD_3_OD): *δ* 22.75, 41.63, 49.85, 53.45, 54.32, 64.82, 64.91, 68.09, 69.91, 70.76, 71.17, 71.21, 71.58, 76.48, 91.04, 123.05, 145.88, 168.25, 170.29, 175.10. 48% yield. Molecular formula: C_38_H_62_N_8_O_21_. HRMS *m*/*z*: calculated for [M + Na]^+^ 989.3922; found 989.3931.

### 1,18-Bis(1-(methyl(5-*N*-acetamido-3,5-dideoxy-d-*glycero*-α-d-*galacto*-2-nonulopyranosyl)-onate)-2-1*H*-1,2,3-triazol-4-yl))-2,5,8,11,14,17-hexaoxaoctadecane (39)

Synthesized according to GP3. ^1^H-NMR (600 MHz, CD_3_OD): *δ* 2.03 (s, 6H), 2.15 (dd, *J* = 13.0 Hz, 11.1 Jz, 2H), 3.27 (dd, *J* = 12.9 Hz, 4.4 Hz, 2H), 3.57 (dd, *J* = 8.8 Hz, 1.3 Hz, 2H), 3.60–3.69 (m, 22H), 3.78–3.85 (m, 6H), 3.84 (s, 6H), 3.88 (dd, *J* = 10.5 Hz, 1.0 Hz, 2H), 3.95 (t, *J* = 10.0 Hz, 2H), 4.04 (ddd, *J* = 11.0 Hz, 9.6 Hz, 4.5 Hz, 2H), 4.65 (s, 4H), 8.35 (s, 2H). ^13^C-NMR (150 MHz, CD_3_OD): *δ* 22.75, 41.64, 53.46, 54.32, 64.82, 64.91, 68.08, 69.89, 70.68, 71.14, 71.18, 71.59, 76.48, 91.04, 123.10, 145.87, 168.25, 170.18, 175.10. 59% yield. Molecular formula: C_40_H_66_N_8_O_22_. HRMS *m*/*z*: calculated for [M + H]^+^ 1011.4364; found 1011.4369.

### 1,15-Bis(1-(5-*N*-acetamido-3,5-dideoxy-d-*glycero*-α-d-*galacto*-2-nonulopyranosylic acid)-2-1*H*-1,2,3-triazol-4-yl)-2,5,8,11,14-pentaoxapentadecane (40)

Synthesized according to GP5. ^1^H-NMR (600 MHz, D_2_O): *δ* 2.05 (s, 6H), 2.23 (dd, *J* = 12.5 Hz, 11.1 Hz, 2H), 3.26 (dd, *J* = 12.4 Hz, 4.3 Hz, 2H), 3.58–3.72 (m, 20H), 3.83–3.89 (m, 4H), 3.90–4.01 (m, 6H), 4.69 (s, 4H), 8.23 (s, 2H). ^13^C-NMR (150 MHz, D_2_O): *δ* 22.03, 39.64, 48.83, 51.51, 62.68, 62.95, 67.99, 68.04, 68.83, 69.45, 69.50, 71.25, 74.09, 90.83, 122.73, 143.62, 171.54, 171.01, 174.96. 88% yield. Molecular formula: C_36_H_58_N_8_O_21_. HRMS *m*/*z*: calculated for [M + H]^+^ 939.3789; found 939.3809.

### 1,18-Bis(1-(5-*N*-acetamido-3,5-dideoxy-d-*glycero*-α-d-*galacto*-2-nonulopyranosylic acid)-2-1*H*-1,2,3-triazol-4-yl)-2,5,8,11,14,17-hexaoxaoctadecane (41)

Synthesized according to GP5. ^1^H-NMR (600 MHz, D_2_O): *δ* 2.05 (s, 6H), 2.23 (dd, *J* = 12.5 Hz, 11.0 Hz, 2H), 3.26 (dd, *J* = 12.4 Hz, 4.5 Hz, 2H), 3.60–3.72 (m, 24H), 3.83–3.89 (m, 4H), 3.90–4.01 (m, 6H), 4.69 (s, 4H), 8.23 (s, 2H). ^13^C-NMR (150 MHz, D_2_O): *δ* 22.04, 39.65, 48.83, 51.51, 62.68, 62.95, 67.98, 68.04, 68.83, 69.41, 69.46, 69.50, 69.52, 71.25, 74.09, 90.83, 122.72, 143.62, 170.53, 171.02, 174.97. 91% yield. Molecular formula: C_38_H_62_N_8_O_22_. HRMS *m*/*z*: calculated for [M + H]^+^ 983.4051; found 983.4057.

## Author contributions

E. J. and R. C. synthesized all compounds. E. J. and R. C. designed all compounds with contributions from G. Z. N. M. evaluated the target compounds in cell-based assays, and performed the thermal stability experiments. G. Z. performed the crystallography experiments. E. J, R. C., M. E., N. A. and T. S., designed the experiments and analysed the data together with all other authors. E. J wrote most of the manuscript with contributions from all other authors. All authors have given approval to the final version of the manuscript.

## Conflicts of interest

There are no conflicts to declare.

## Supplementary Material

RA-012-D1RA08968D-s001
