# Supplementary material for: Exploring divalent conjugates of 5-N-acetyl-neuraminic acid as inhibitors of coxsackievirus A24 variant (CVA24v) transduction
Source: RSC Adv. 2022 Jan 14;12(4):2319–31. doi: 10.1039/d1ra08968d (PMC8979015; doi:10.1039/d1ra08968d)

Electronic Supplementary Material (ESI) for RSC Advances.
This journal is © The Royal Society of Chemistry 2022

Supplementary Information for:

Exploring divalent conjugates of 5-*N*-acetyl-neuraminic acid as inhibitors of coxsackievirus A24 variant (CVA24v) transduction

Emil Johansson, Rémi Caraballo, Georg Zocher, Nitesh Mistry, Niklas Arnberg, Thilo Stehle, and Mikael Elofsson*

*Professor Mikael Elofsson
Email: mikael.elofsson@umu.se

This PDF file includes:

^{1}H-NMR and ^{13}C-NMR spectra for compounds **3**, **10–21**, **25–34**, and **38–41**.

Compound 3

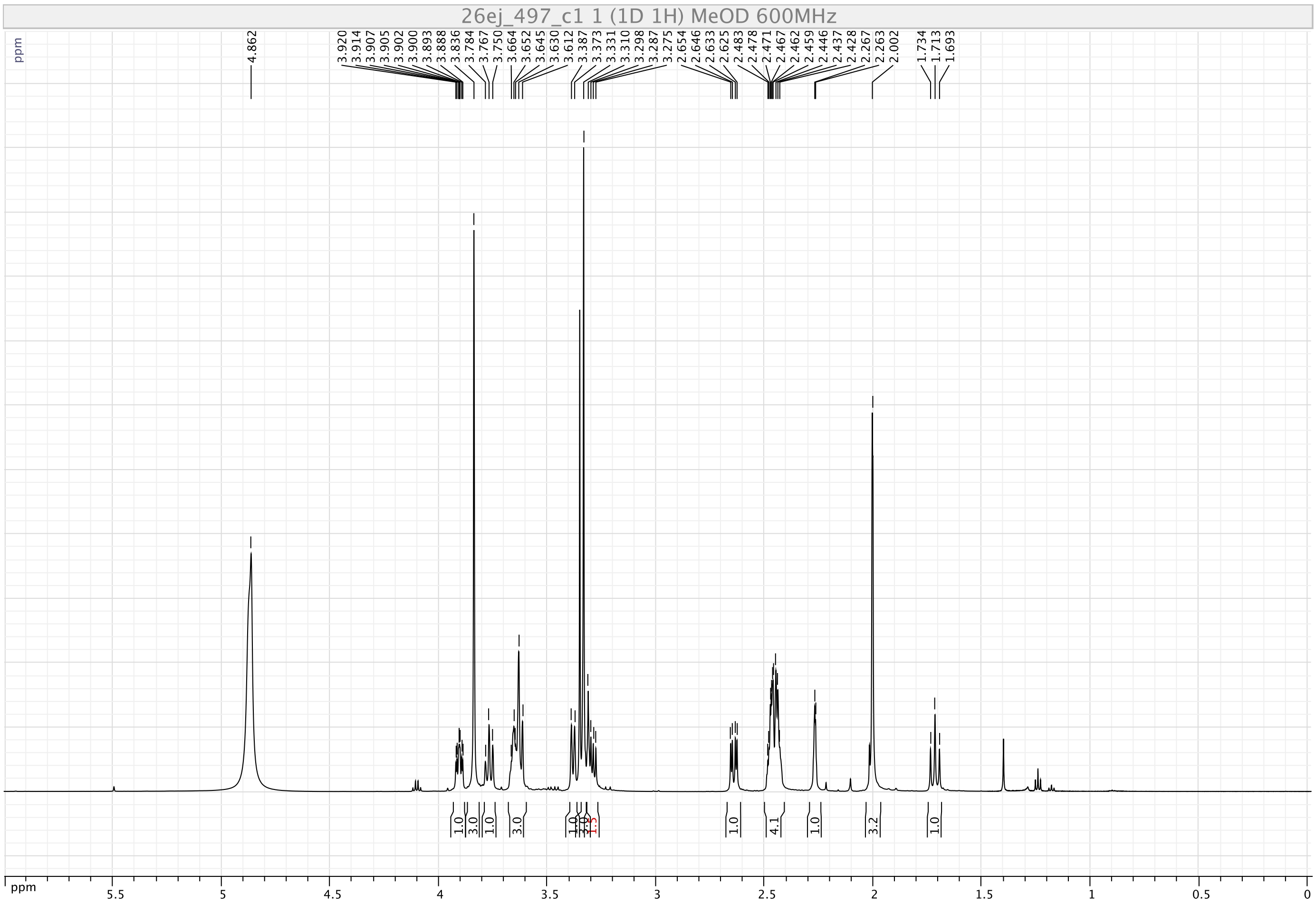

Compound 3

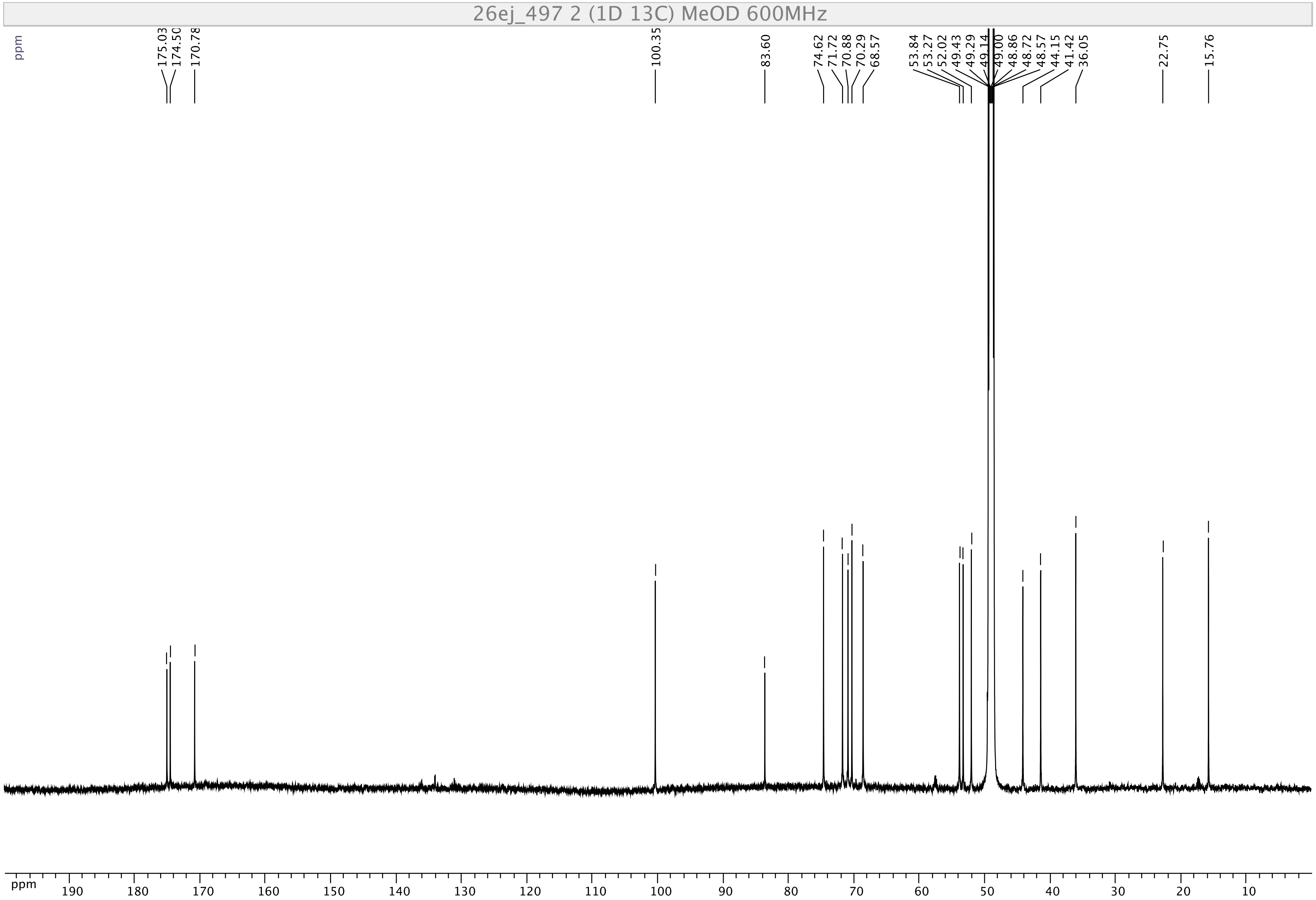

26ej_497 2 (1D 13C) MeOD 600MHz
ppm
175.03
174.50
170.78
100.35
83.60
74.62
71.72
70.88
70.29
68.57
53.84
53.27
52.02
49.43
49.29
49.14
49.00
48.86
48.72
48.57
44.15
41.42
36.05
22.75
15.76
ppm
190
180
170
160
150
140
130
120
110
100
90
80
70
60
50
40
30
20
10

Compound 10

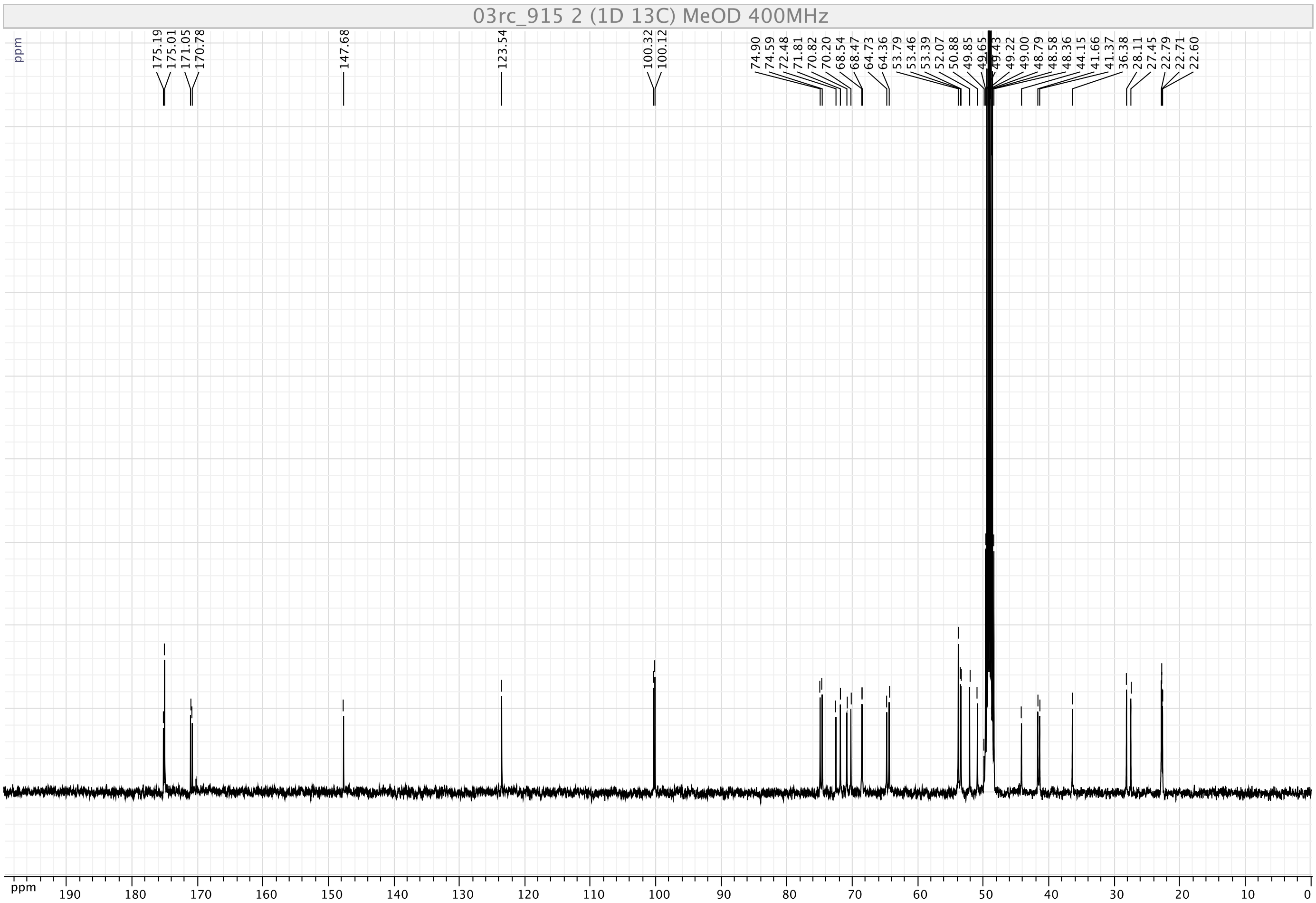
03rc_915 2 (1D 13C) MeOD 400MHz
175.19
175.01
171.05
170.78
147.68
123.54
100.32
100.12
74.90
74.59
72.48
71.81
70.82
70.20
68.54
68.47
64.73
64.36
53.79
53.46
53.39
52.07
50.88
49.85
49.65
49.22
49.00
48.79
48.58
48.36
44.15
41.66
41.37
36.38
28.11
27.45
22.79
22.71
22.60
ppm

Compound 10

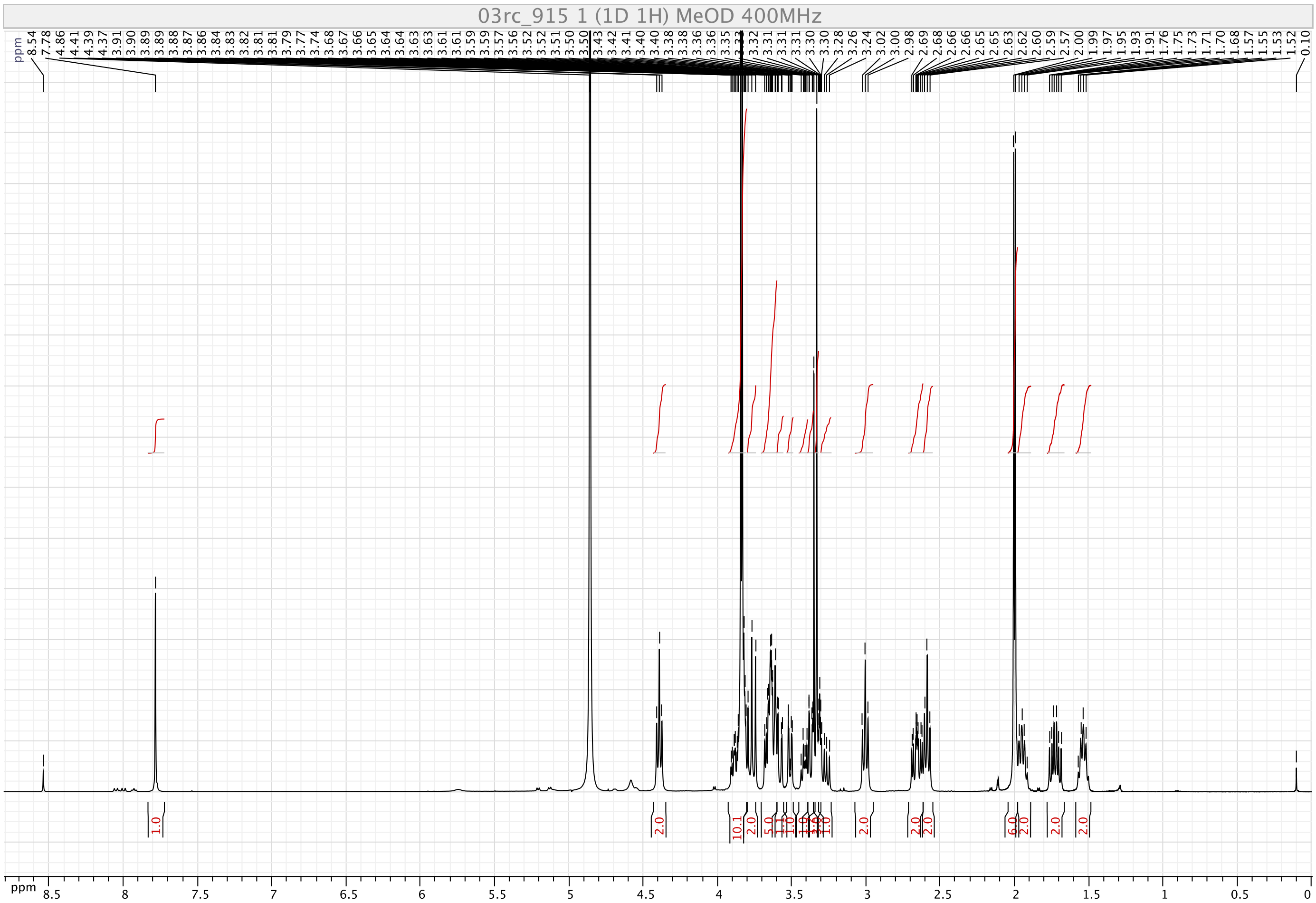

Compound 11

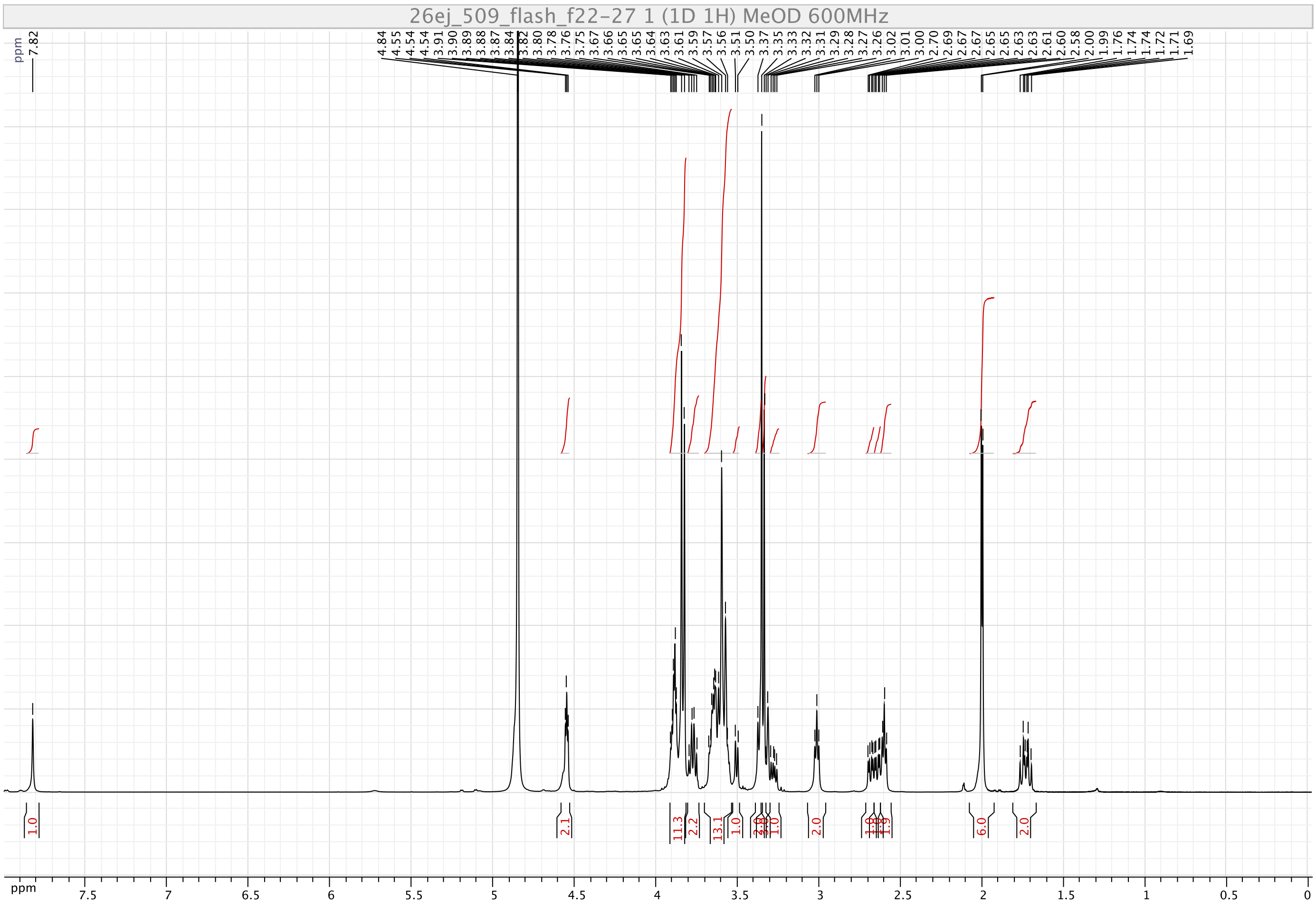

Compound 11

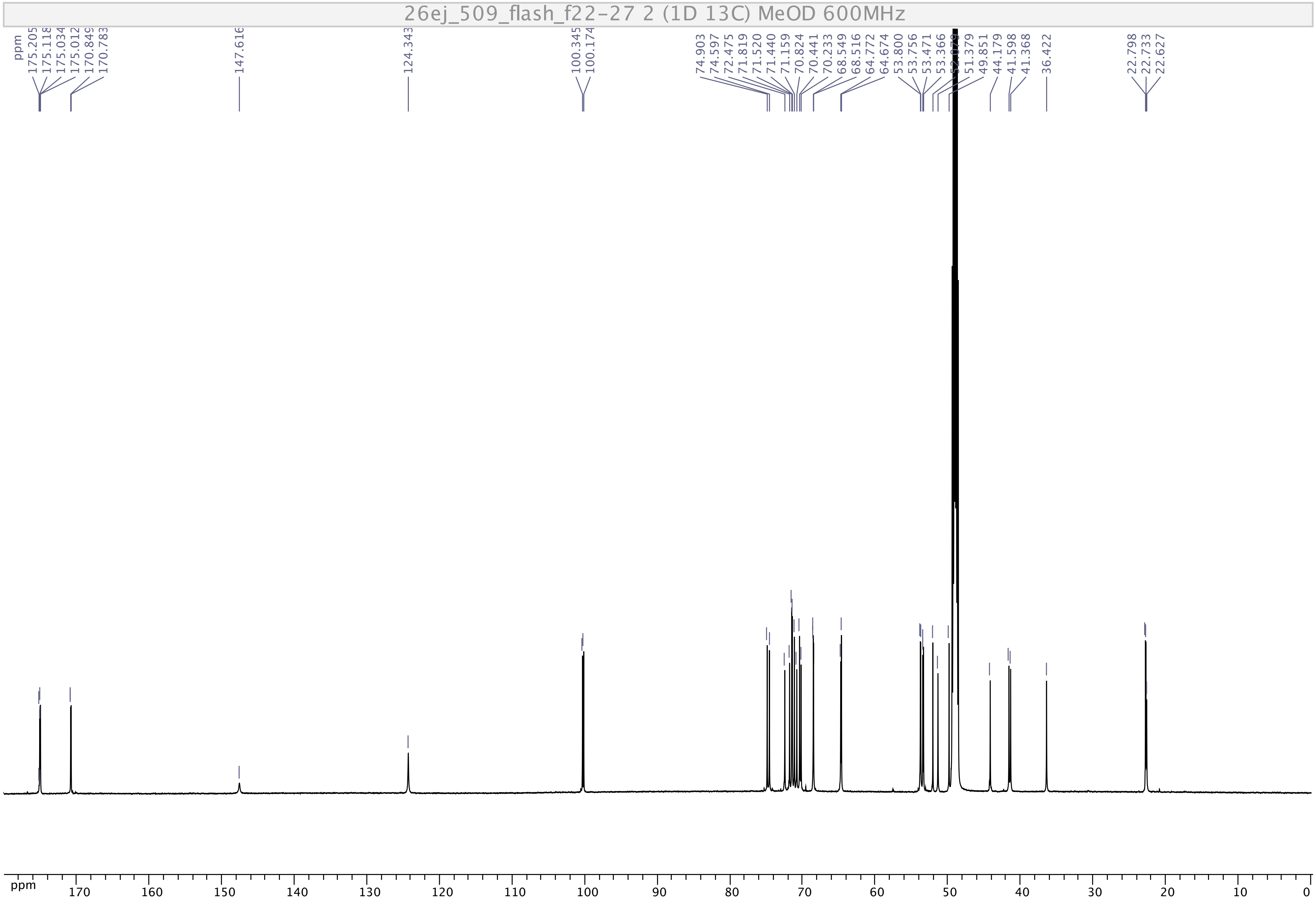
26ej_509_flash_f22-27 2 (1D 13C) MeOD 600MHz
ppm
175.205
175.118
175.034
175.012
170.849
170.783
147.616
124.343
100.345
100.174
74.903
74.597
72.475
71.819
71.520
71.440
71.159
70.824
70.441
70.233
68.549
68.516
64.772
64.674
53.800
53.756
53.471
53.366
51.379
49.851
44.179
41.598
41.368
36.422
22.798
22.733
22.627
ppm
170
160
150
140
130
120
110
100
90
80
70
60
50
40
30
20
10
0

Compound 12

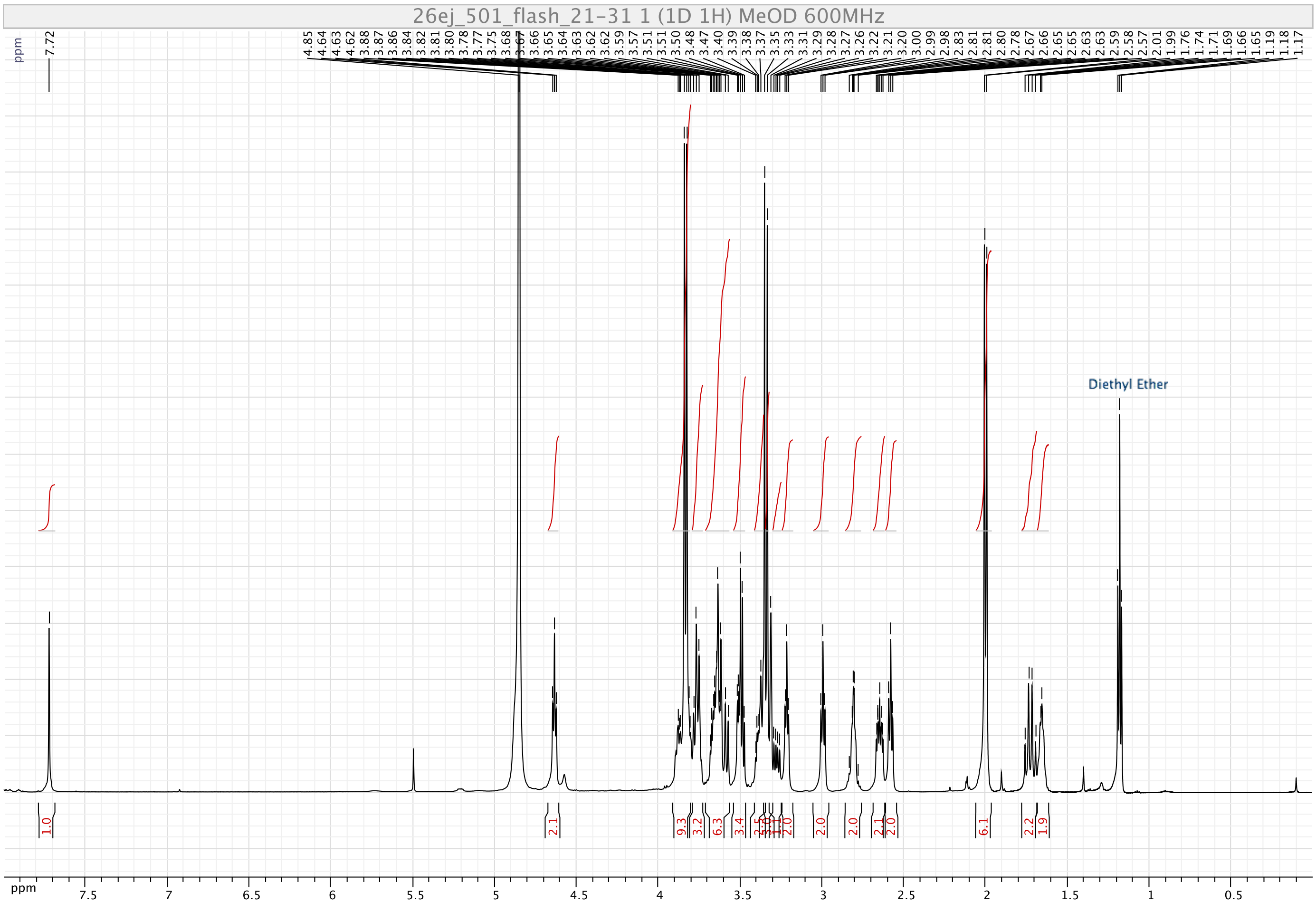
26ej_501_flash_21-31 1 (1D 1H) MeOD 600MHz
ppm
7.72
4.85
4.64
4.63
4.62
3.88
3.87
3.86
3.84
3.82
3.81
3.80
3.78
3.77
3.75
3.68
3.67
3.66
3.65
3.64
3.63
3.62
3.62
3.59
3.57
3.51
3.50
3.48
3.47
3.40
3.39
3.38
3.37
3.35
3.33
3.31
3.29
3.28
3.27
3.26
3.22
3.21
3.20
3.00
2.99
2.98
2.83
2.81
2.81
2.80
2.78
2.67
2.66
2.65
2.65
2.63
2.63
2.59
2.58
2.57
2.01
1.99
1.76
1.74
1.71
1.69
1.66
1.65
1.19
1.18
1.17
Diethyl Ether
1.0
2.1
9.3
3.2
6.3
3.4
2.0
2.0
2.0
2.1
2.0
6.1
2.2
1.9
ppm
7.5
7
6.5
6
5.5
5
4.5
4
3.5
3
2.5
2
1.5
1
0.5

Compound 12

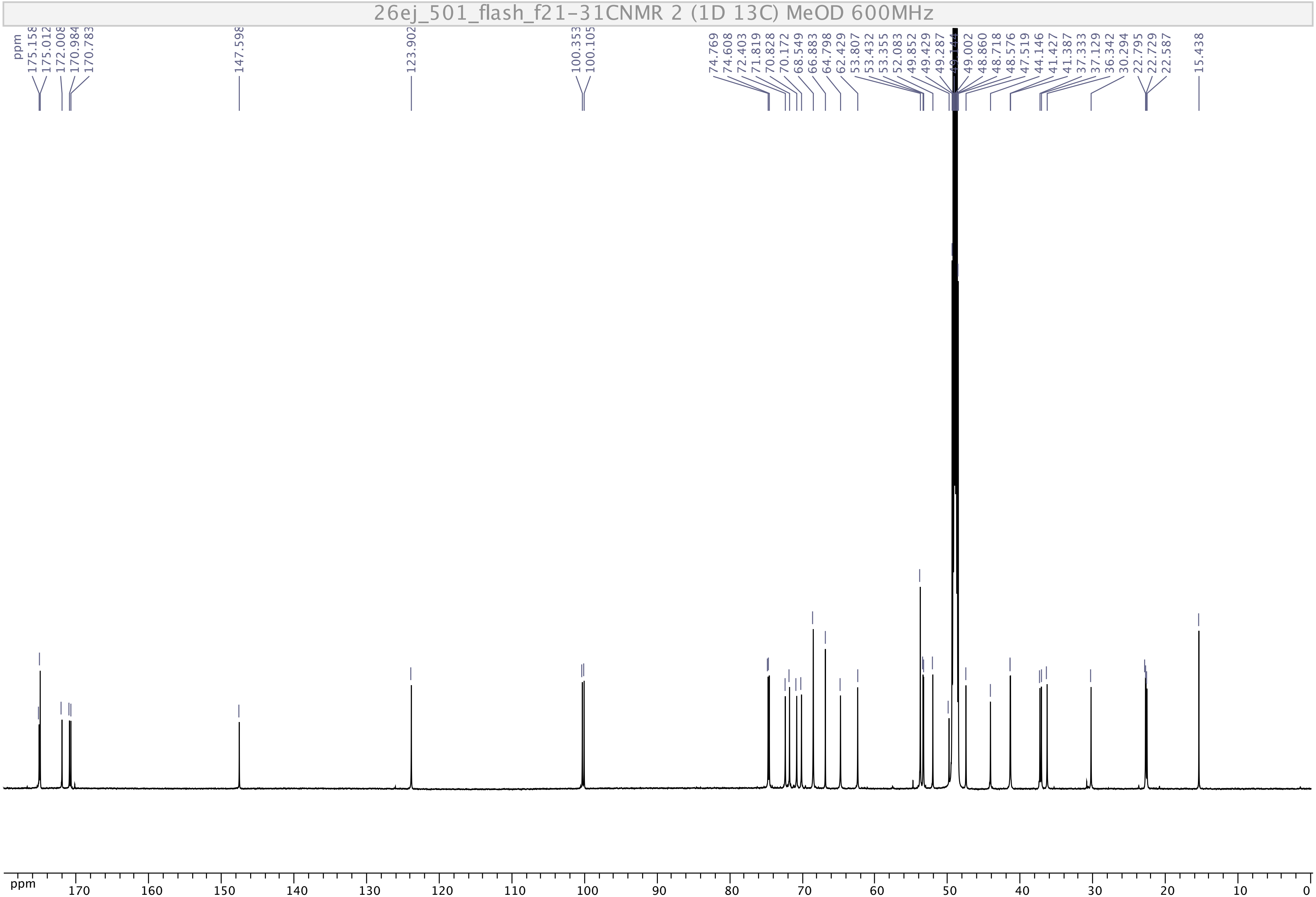
26ej_501_flash_f21-31CNMR 2 (1D 13C) MeOD 600MHz
ppm
175.158
175.012
172.008
170.984
170.783
147.598
123.902
100.353
100.105
74.769
74.608
72.403
71.819
70.828
70.172
68.549
66.883
64.798
62.429
53.807
53.432
53.355
52.083
49.852
49.429
49.287
49.144
49.002
48.860
48.718
48.576
47.519
44.146
41.427
41.387
37.333
37.129
36.342
30.294
22.795
22.729
22.587
15.438
ppm
170
160
150
140
130
120
110
100
90
80
70
60
50
40
30
20
10
0

Compound 13

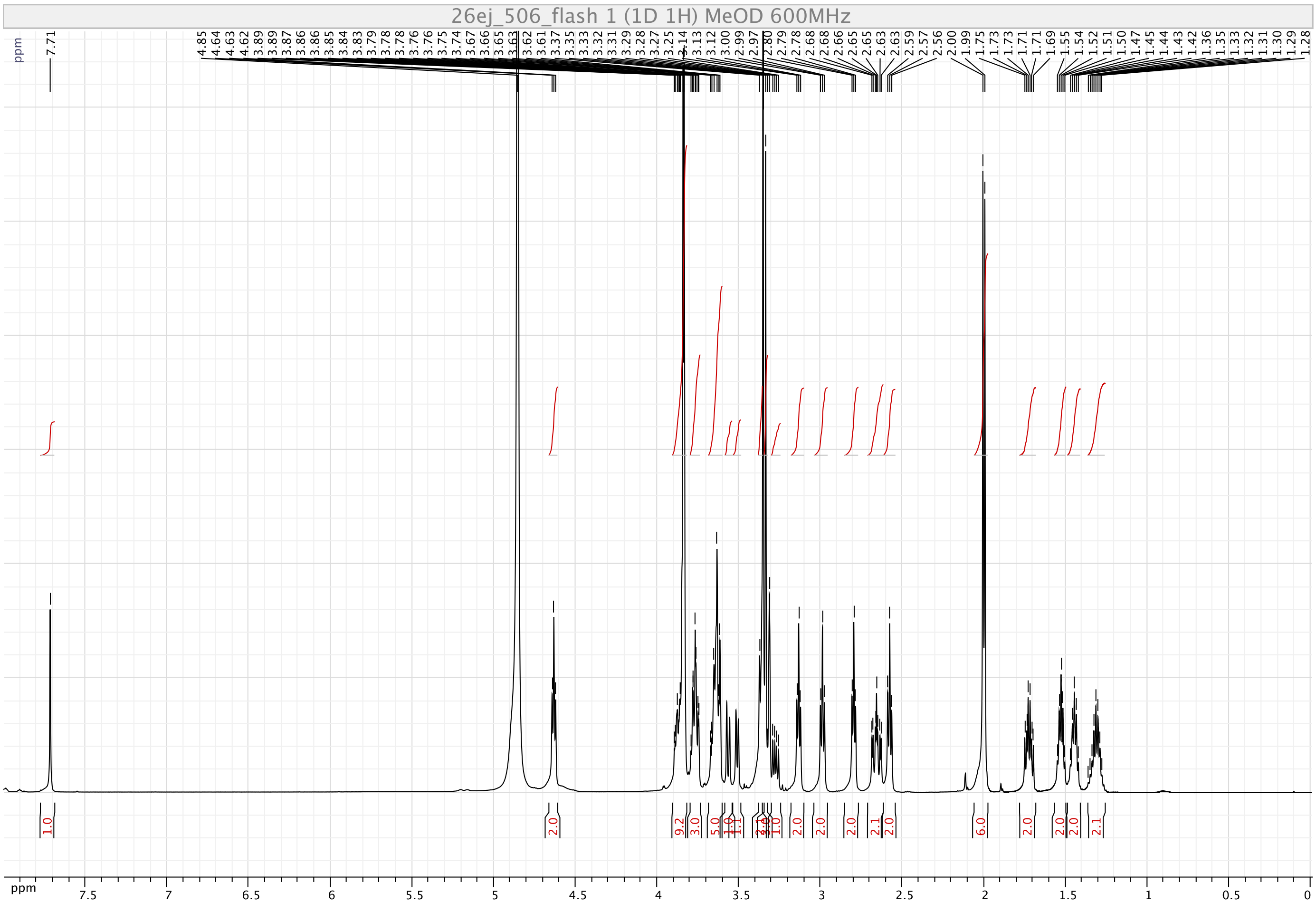

Compound 13

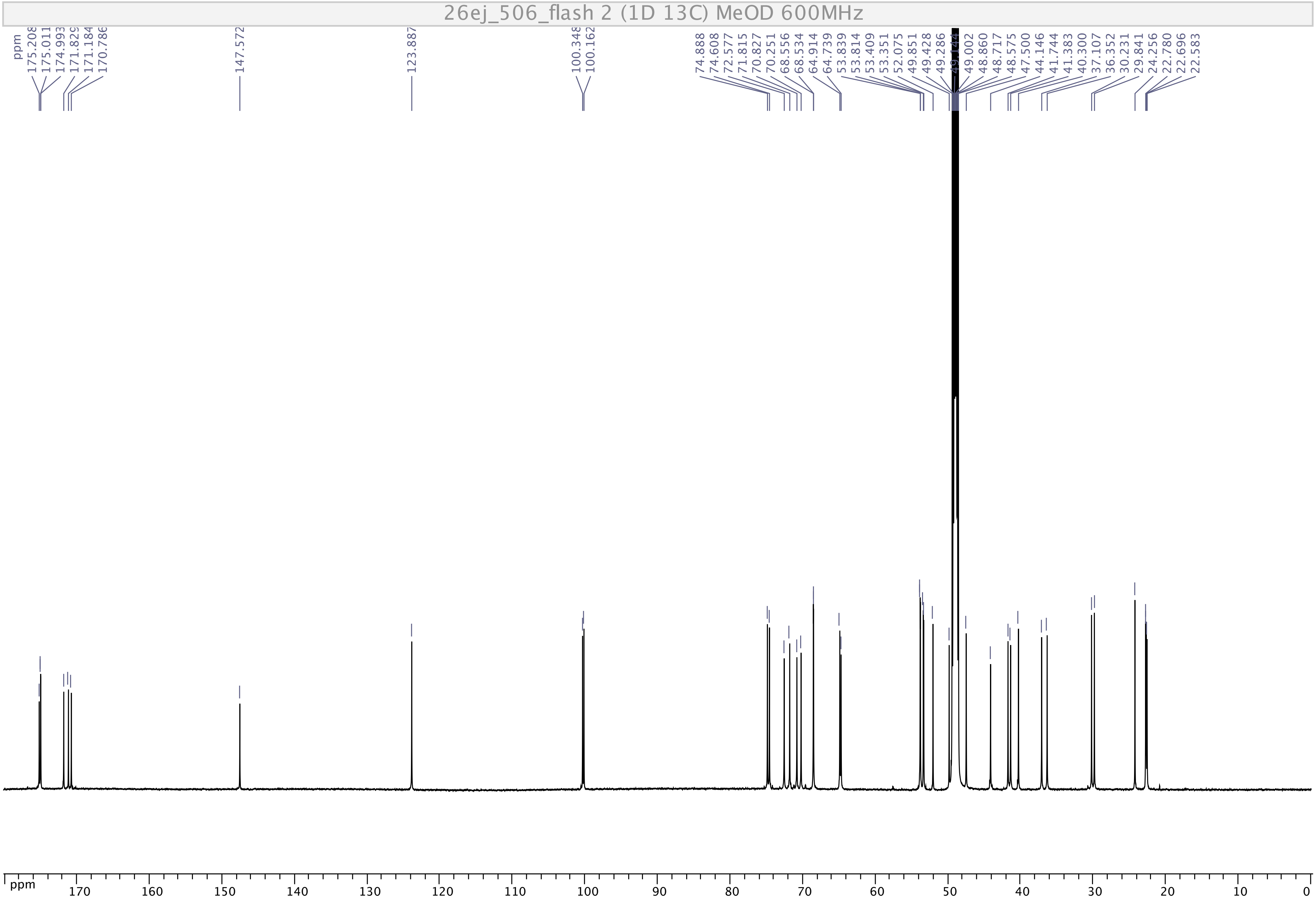
26ej_506_flash 2 (1D 13C) MeOD 600MHz
ppm
175.208
175.011
174.993
171.829
171.184
170.786
147.572
123.887
100.348
100.162
74.888
74.608
72.577
71.815
70.827
70.251
68.556
68.534
64.914
64.739
53.839
53.814
53.409
53.351
52.075
49.851
49.428
49.286
49.144
49.002
48.860
48.717
48.575
47.500
44.146
41.744
41.383
40.300
37.107
36.352
30.231
29.841
24.256
22.780
22.696
22.583
ppm
170
160
150
140
130
120
110
100
90
80
70
60
50
40
30
20
10
0

Compound 14

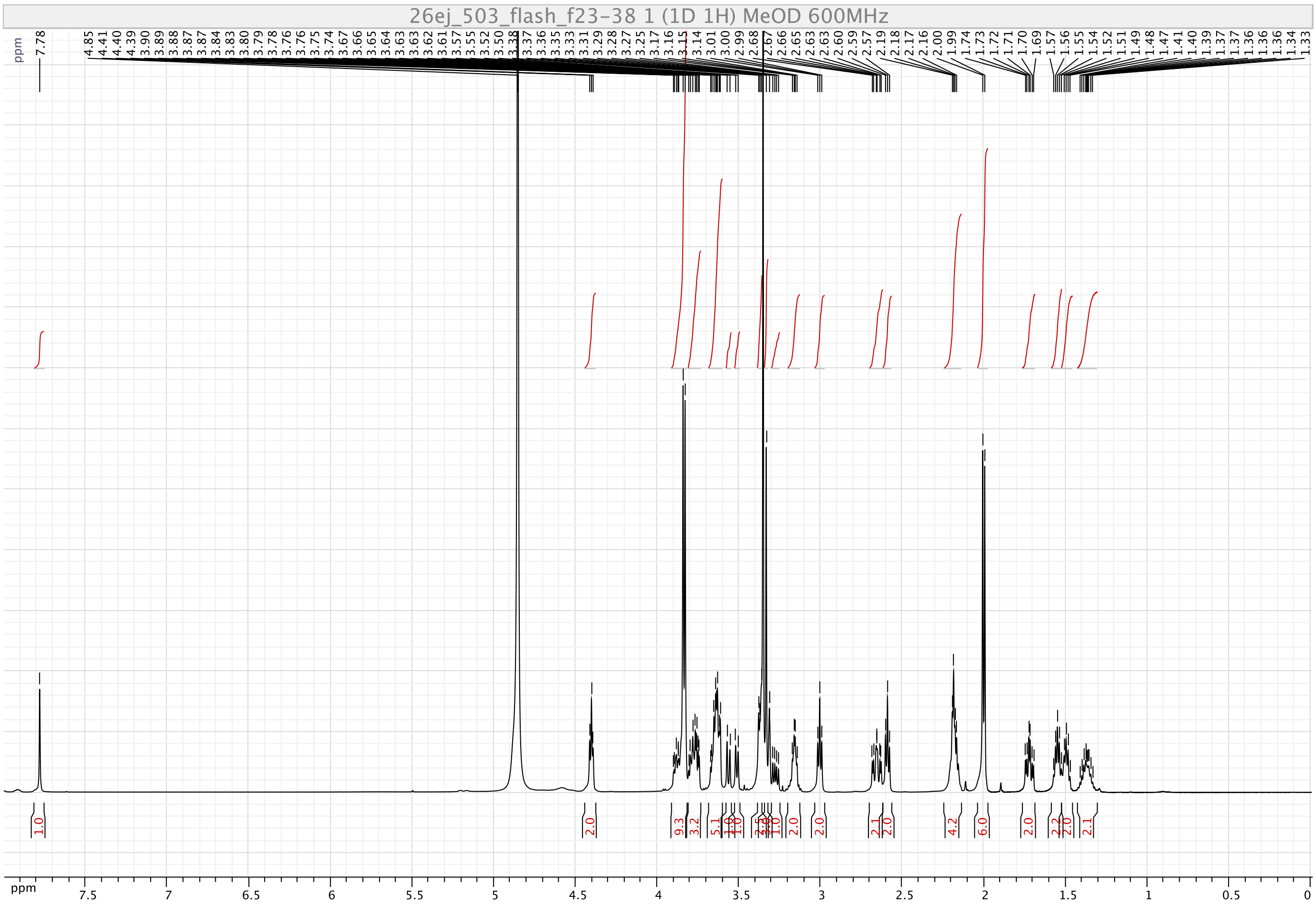

Compound 14

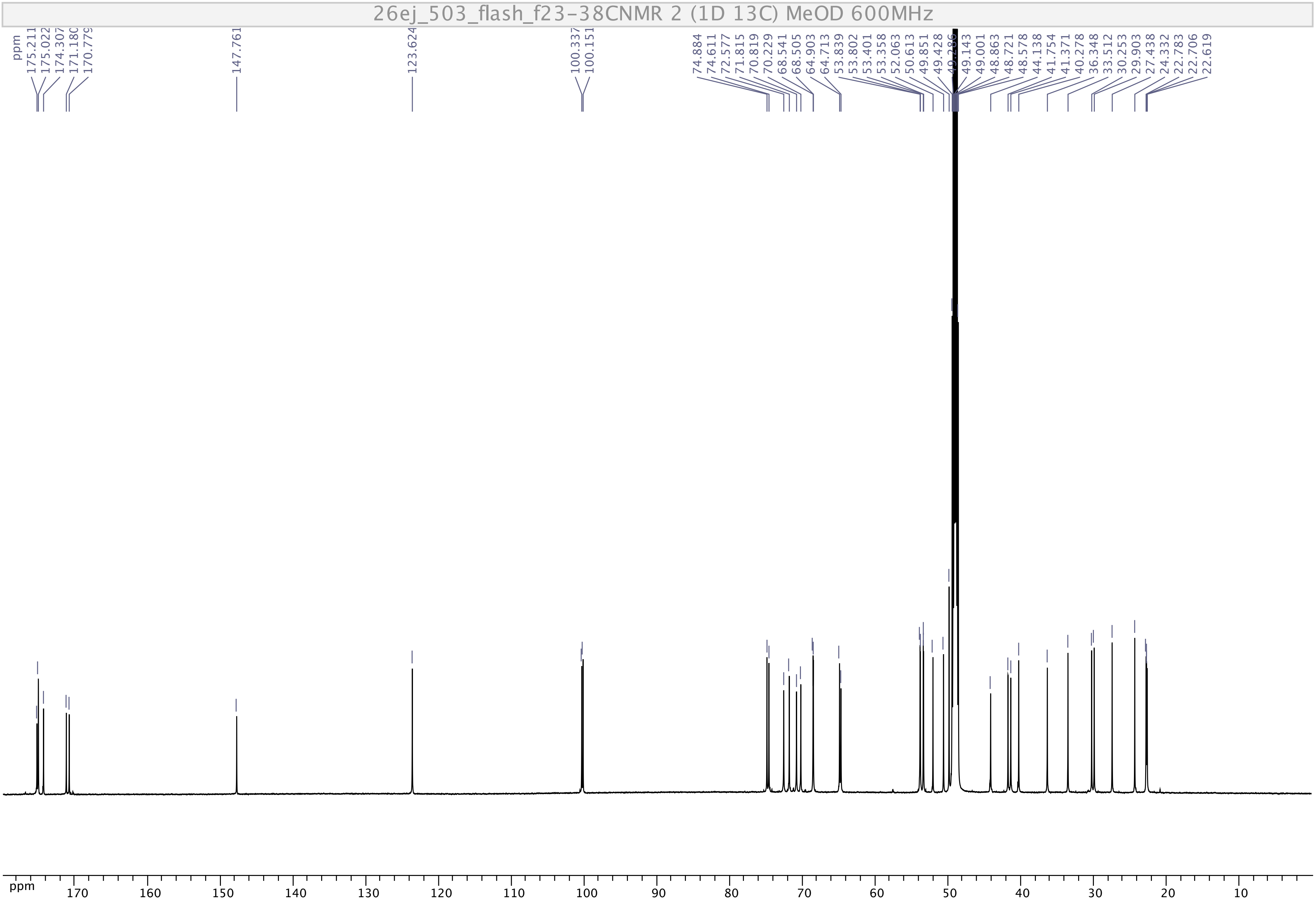
26ej_503_flash_f23-38CNMR 2 (1D 13C) MeOD 600MHz
ppm
175.211
175.022
174.307
171.180
170.779
147.761
123.624
100.337
100.151
74.884
74.611
72.577
71.815
70.819
70.229
68.541
68.505
64.903
64.713
53.839
53.802
53.401
53.358
52.063
50.613
49.851
49.428
49.286
49.143
49.001
48.863
48.721
48.578
44.138
41.754
41.371
40.278
36.348
33.512
30.253
29.903
27.438
24.332
22.783
22.706
22.619
ppm
170
160
150
140
130
120
110
100
90
80
70
60
50
40
30
20
10

Compound 15

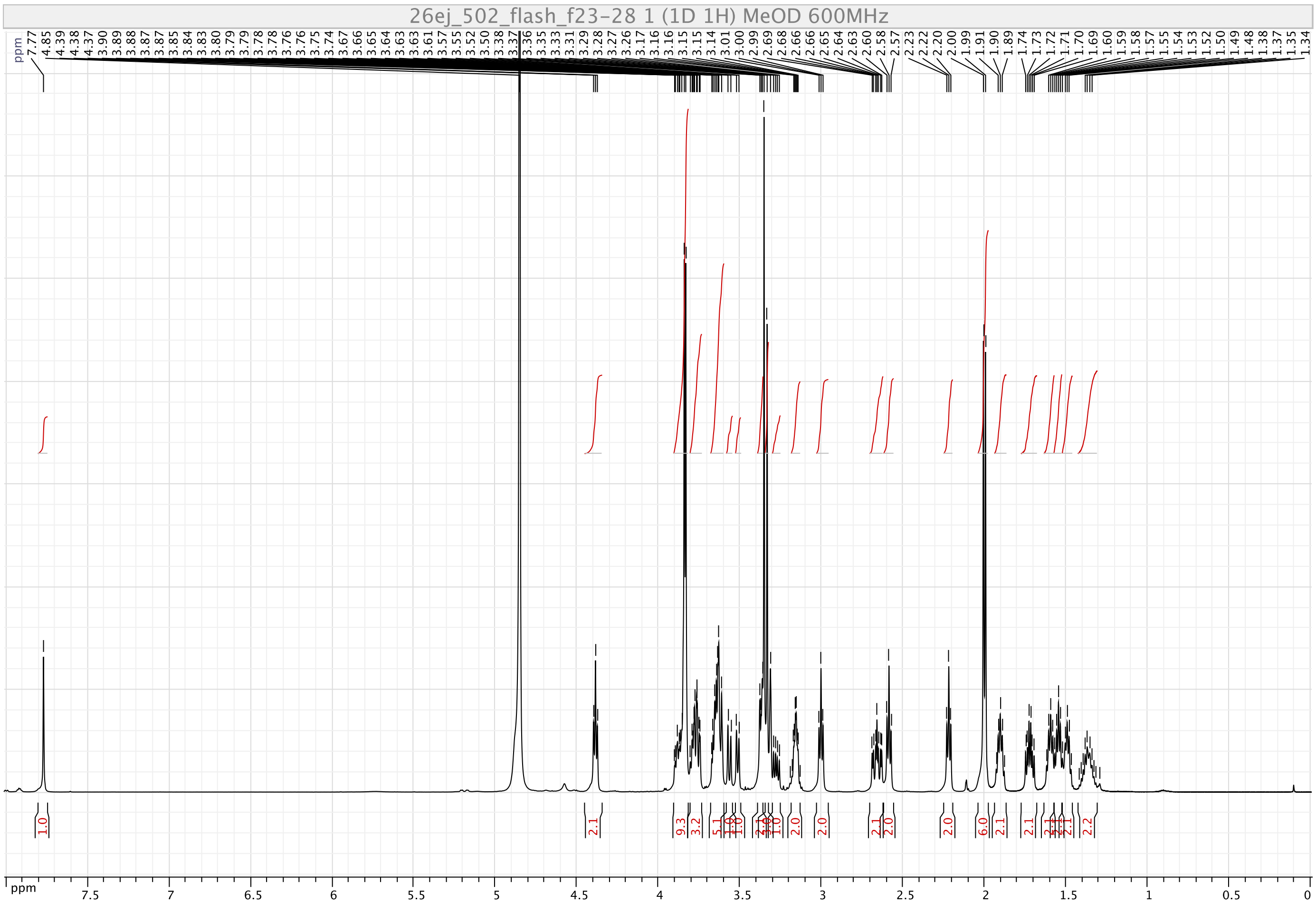

Compound 15

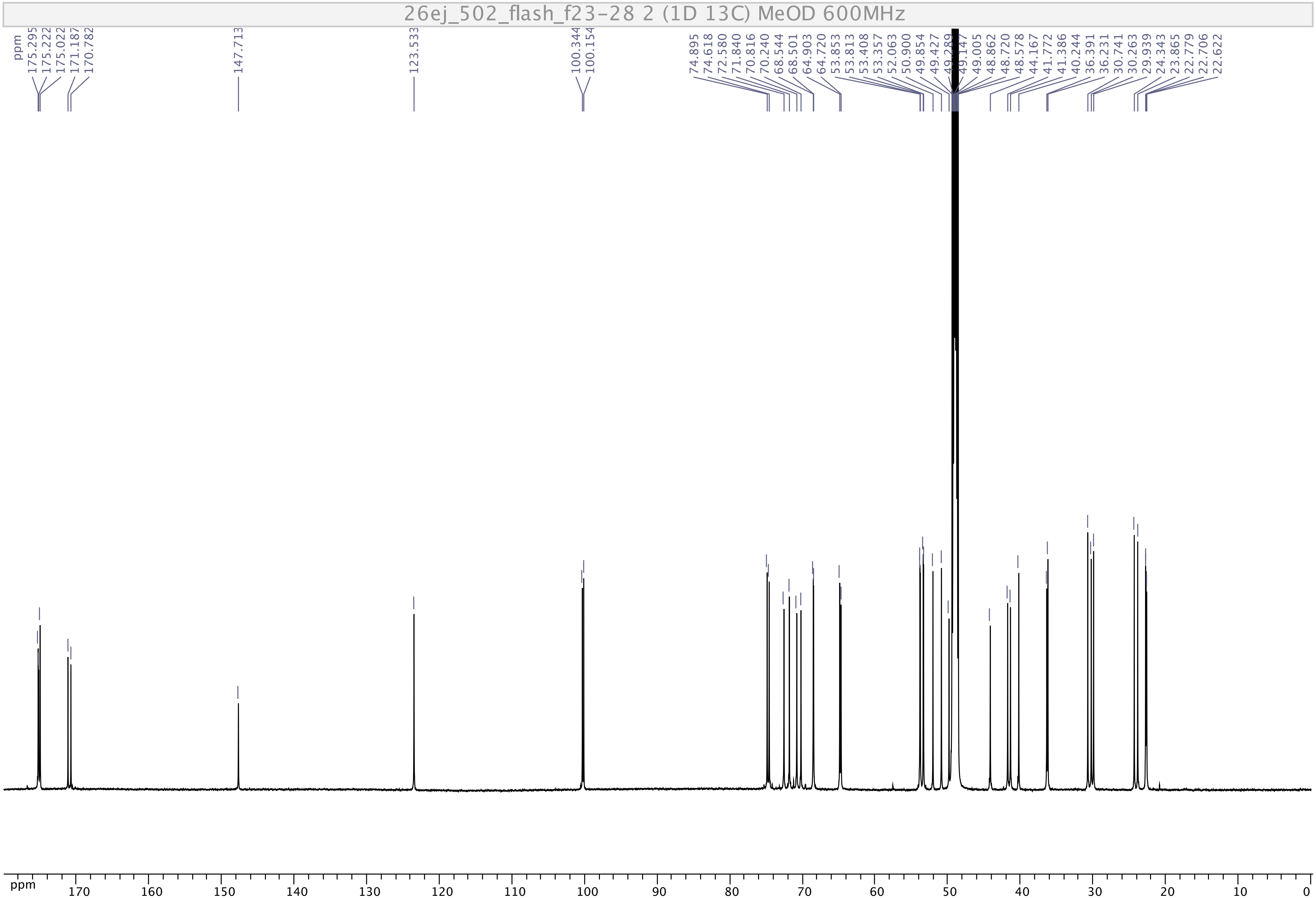
26ej_502_flash_f23-28 2 (1D 13C) MeOD 600MHz
ppm
175.295
175.222
175.022
171.187
170.782
147.713
123.533
100.344
100.154
74.895
74.618
72.580
71.840
70.816
70.240
68.544
68.501
64.903
64.720
53.853
53.813
53.408
53.357
52.063
50.900
49.854
49.427
49.147
49.005
48.862
48.720
48.578
44.167
41.772
41.386
40.244
36.391
36.231
30.741
30.263
29.939
24.343
23.865
22.779
22.706
22.622
ppm
170
160
150
140
130
120
110
100
90
80
70
60
50
40
30
20
10
0

Compound 15 (ME1064)

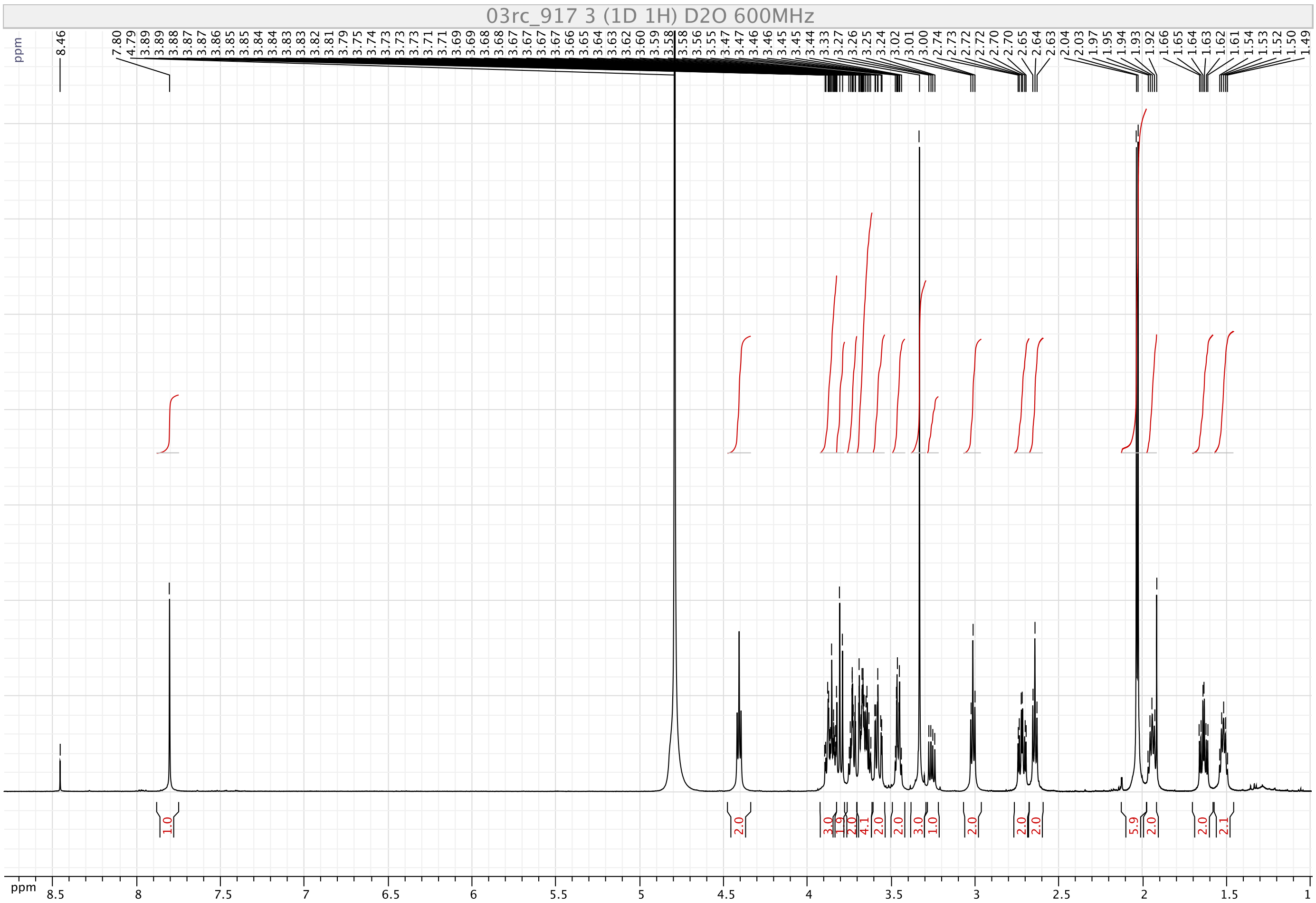

Compound 15 (ME1064)

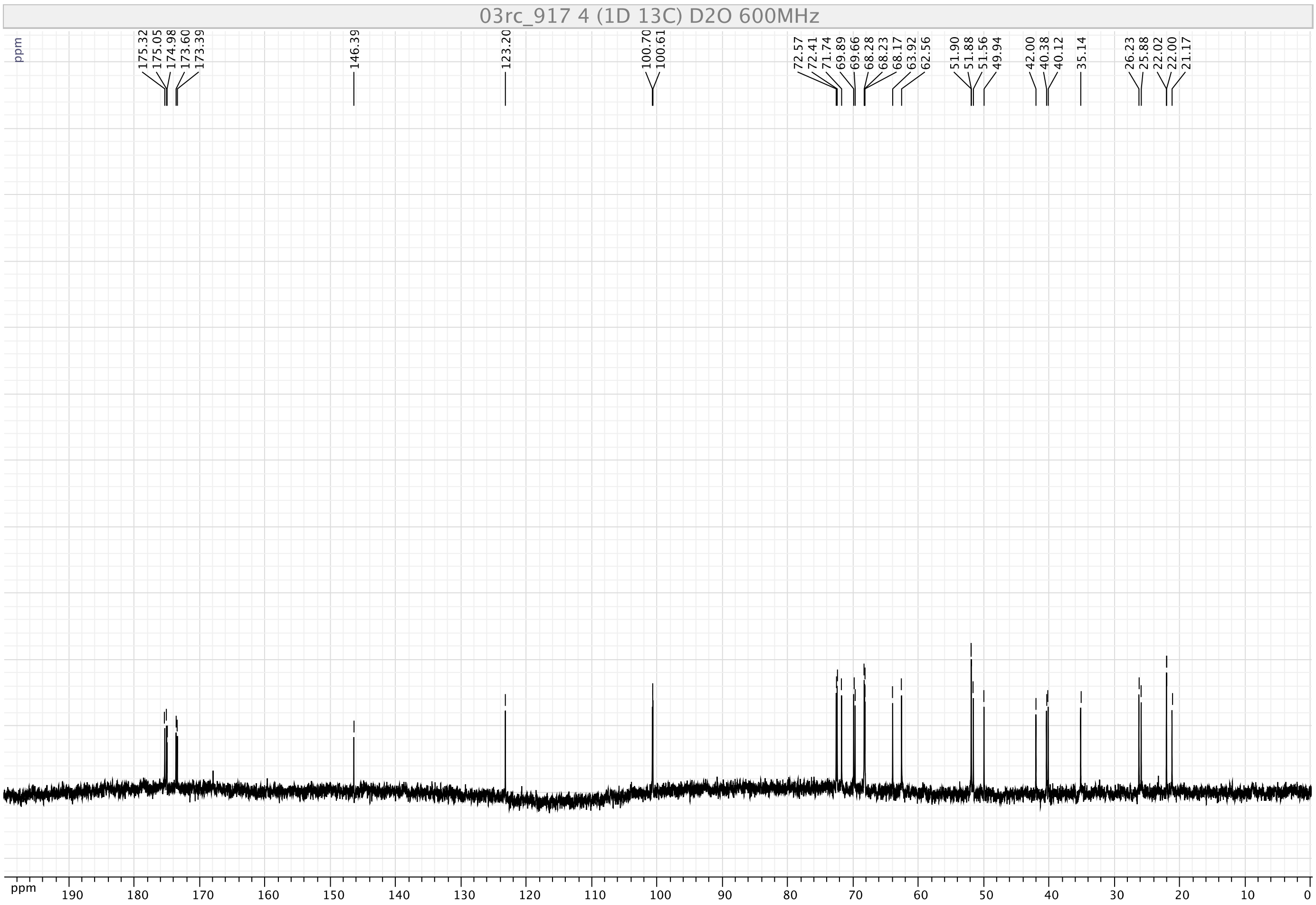

Compound 17 (ME1067)

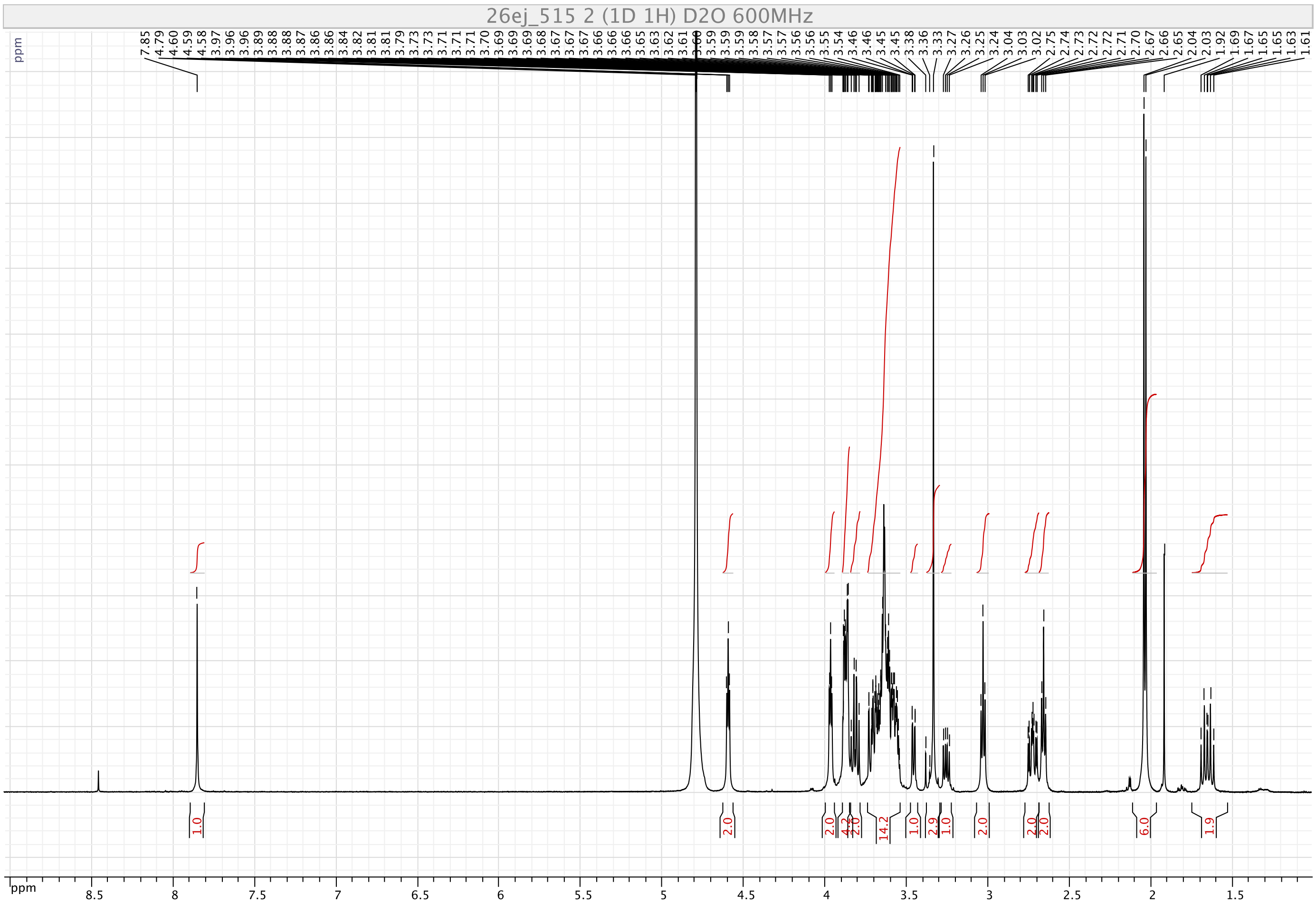

Compound 17 (ME1067)

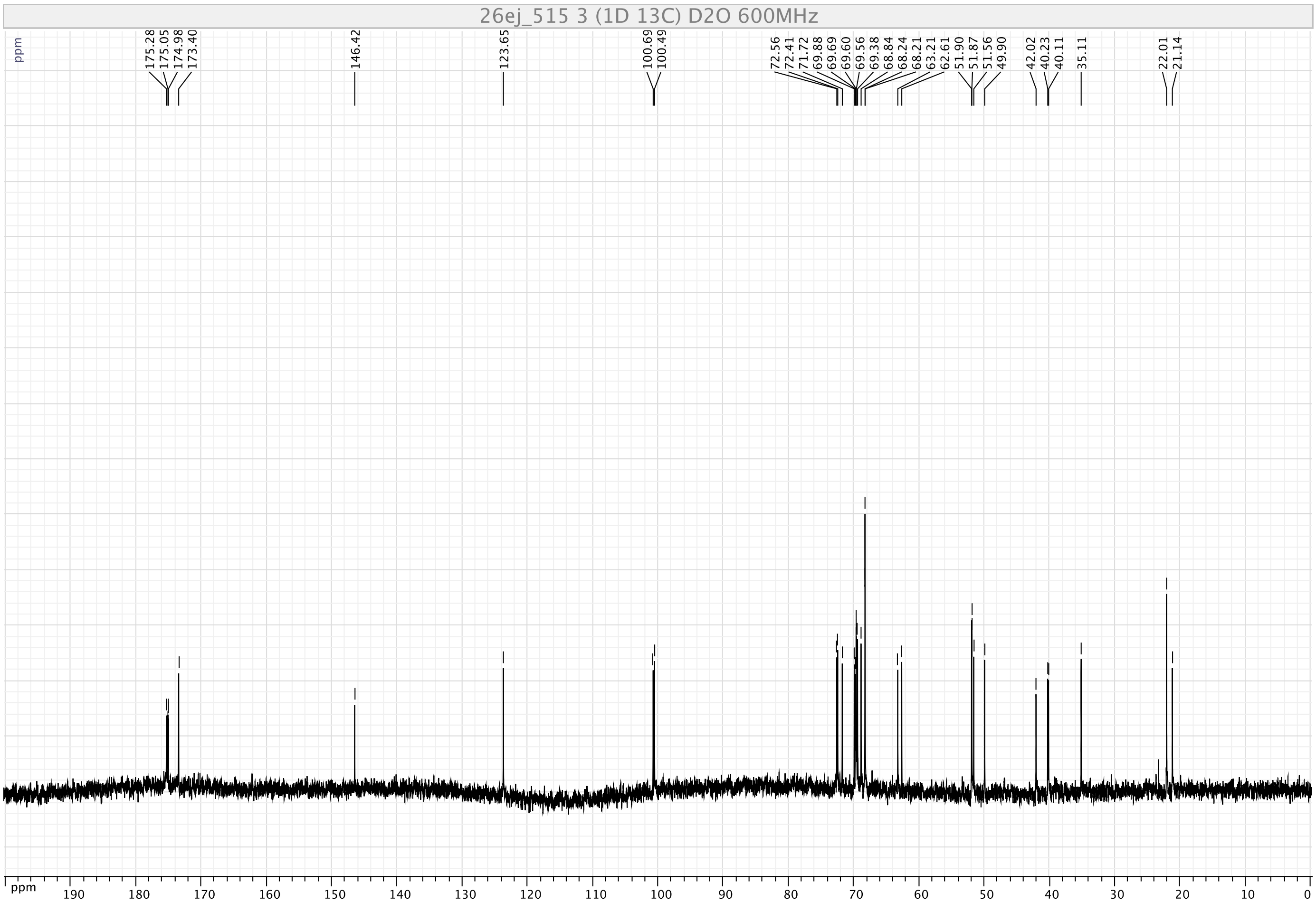

Compound 18 (ME1065)

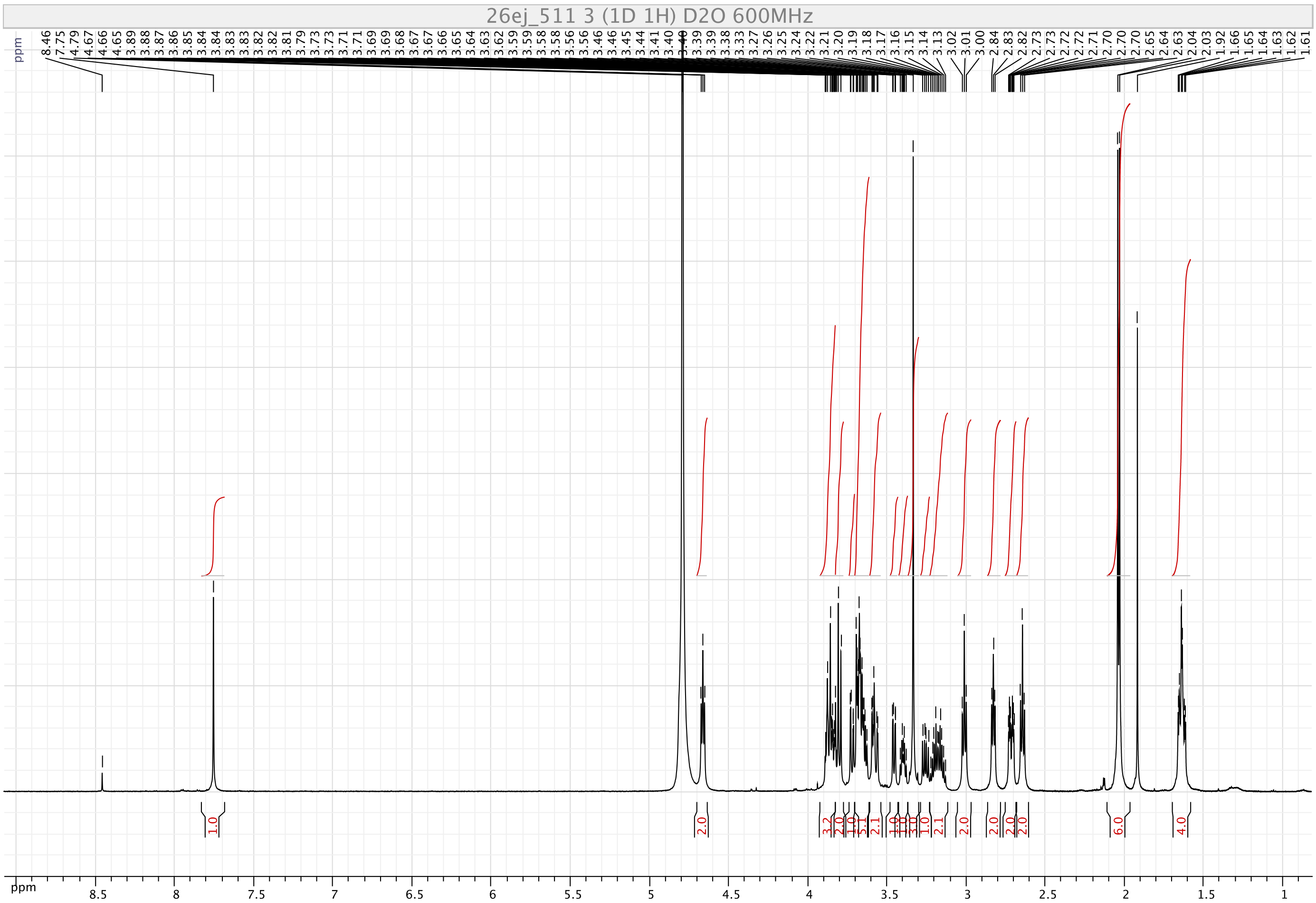

Compound 18 (ME1065)

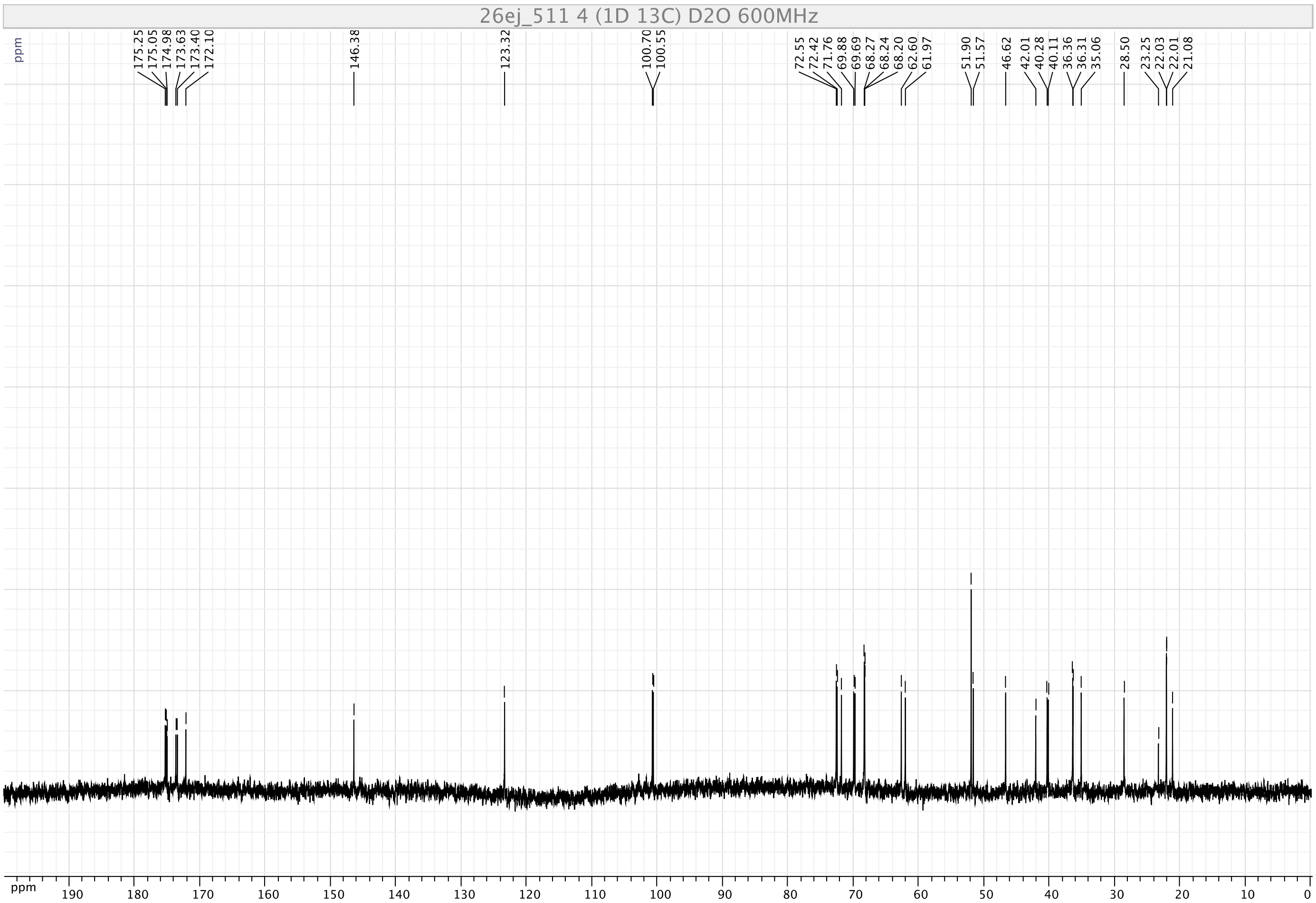

Compound 19 (ME1068)

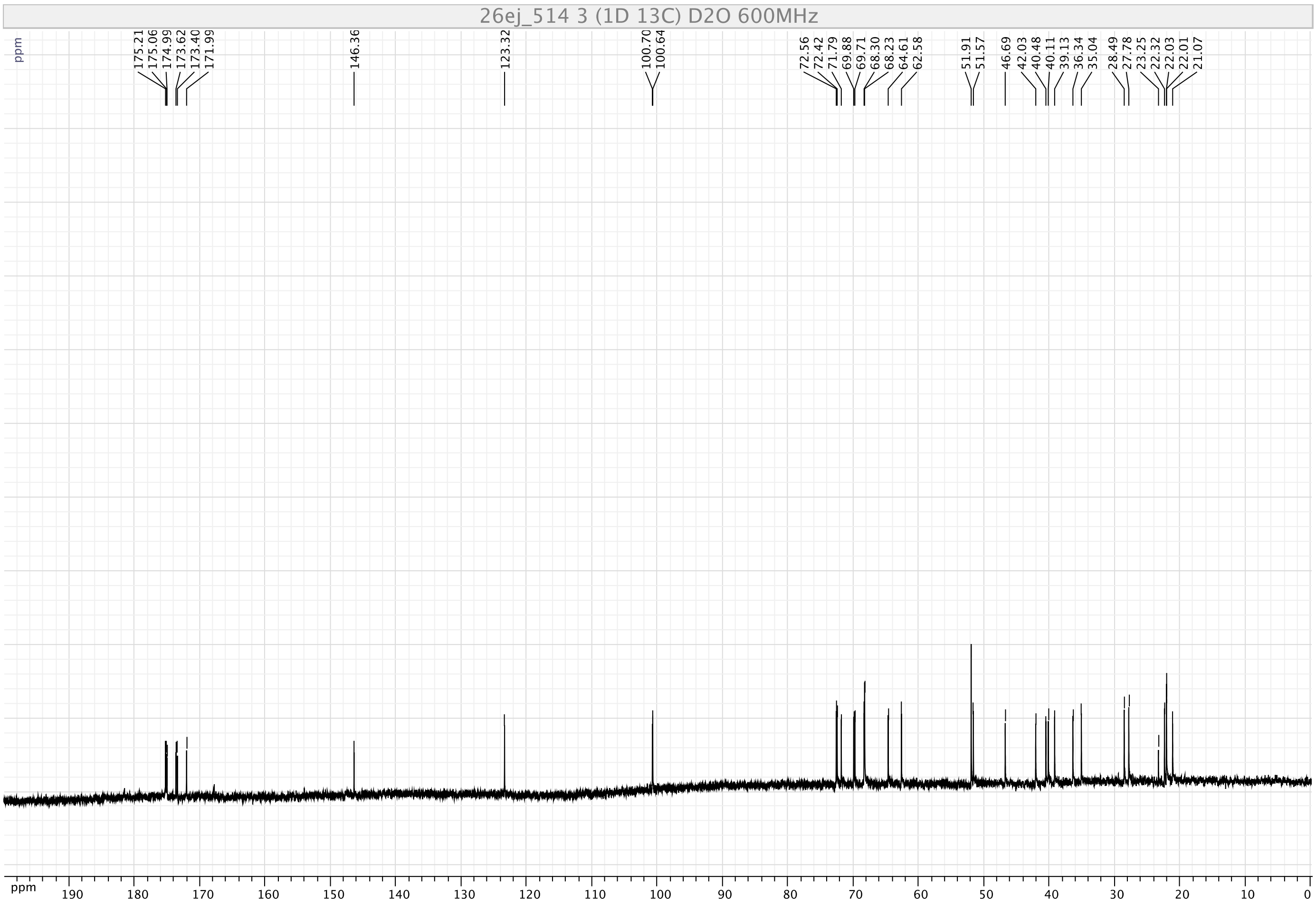

Compound 19 (ME1068)

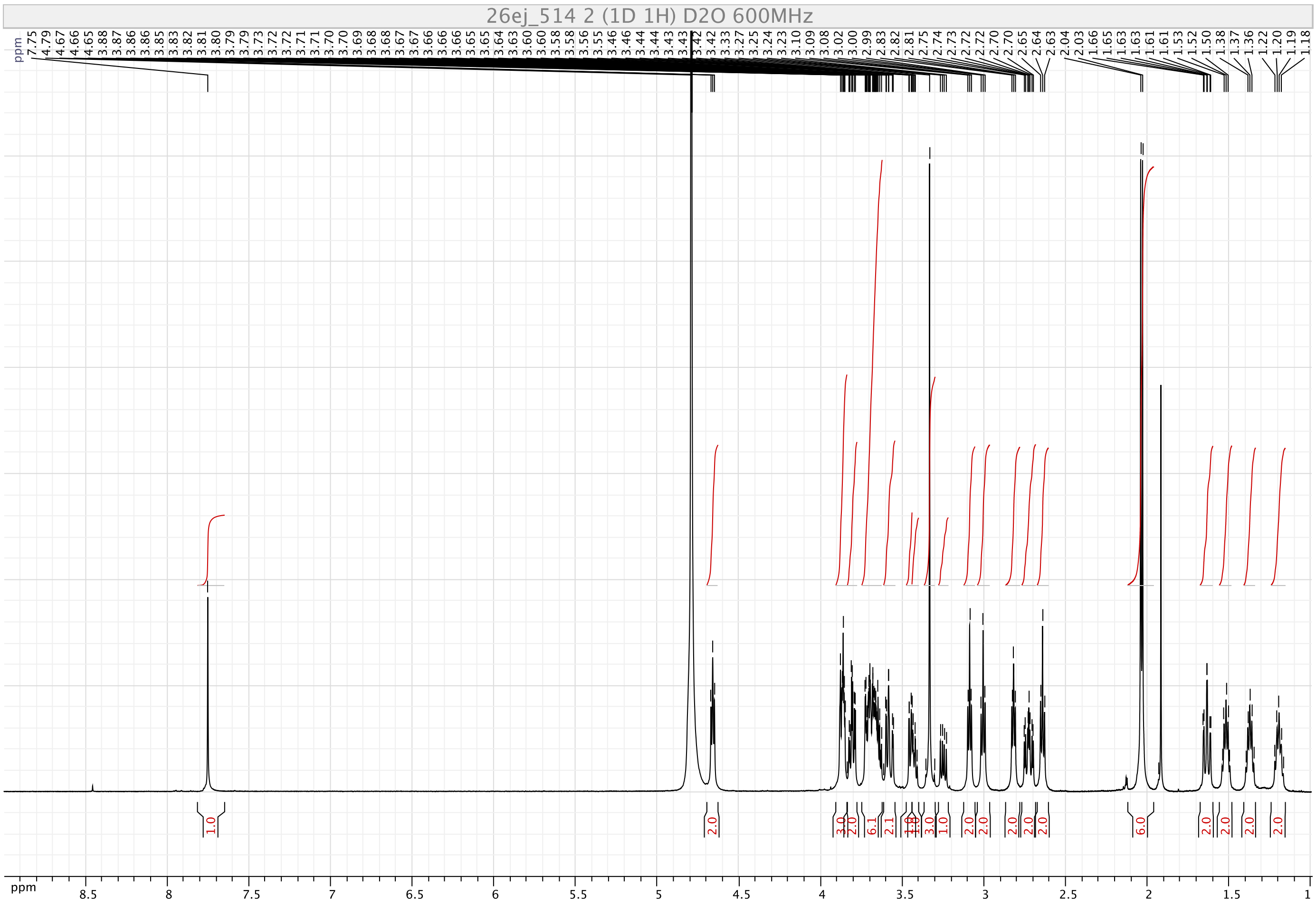

Compound 20 (ME1069)

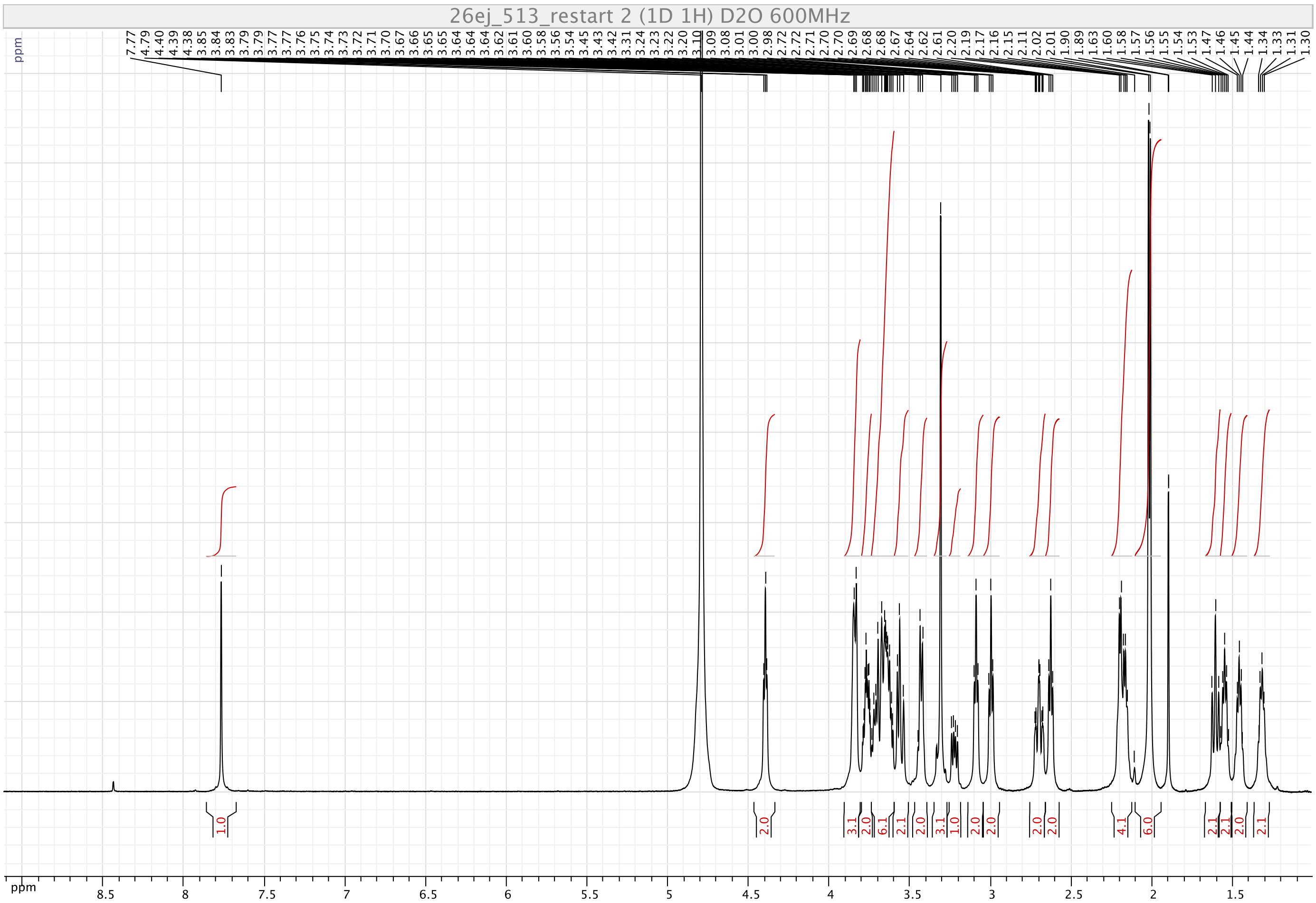

Compound 20 (ME1069)

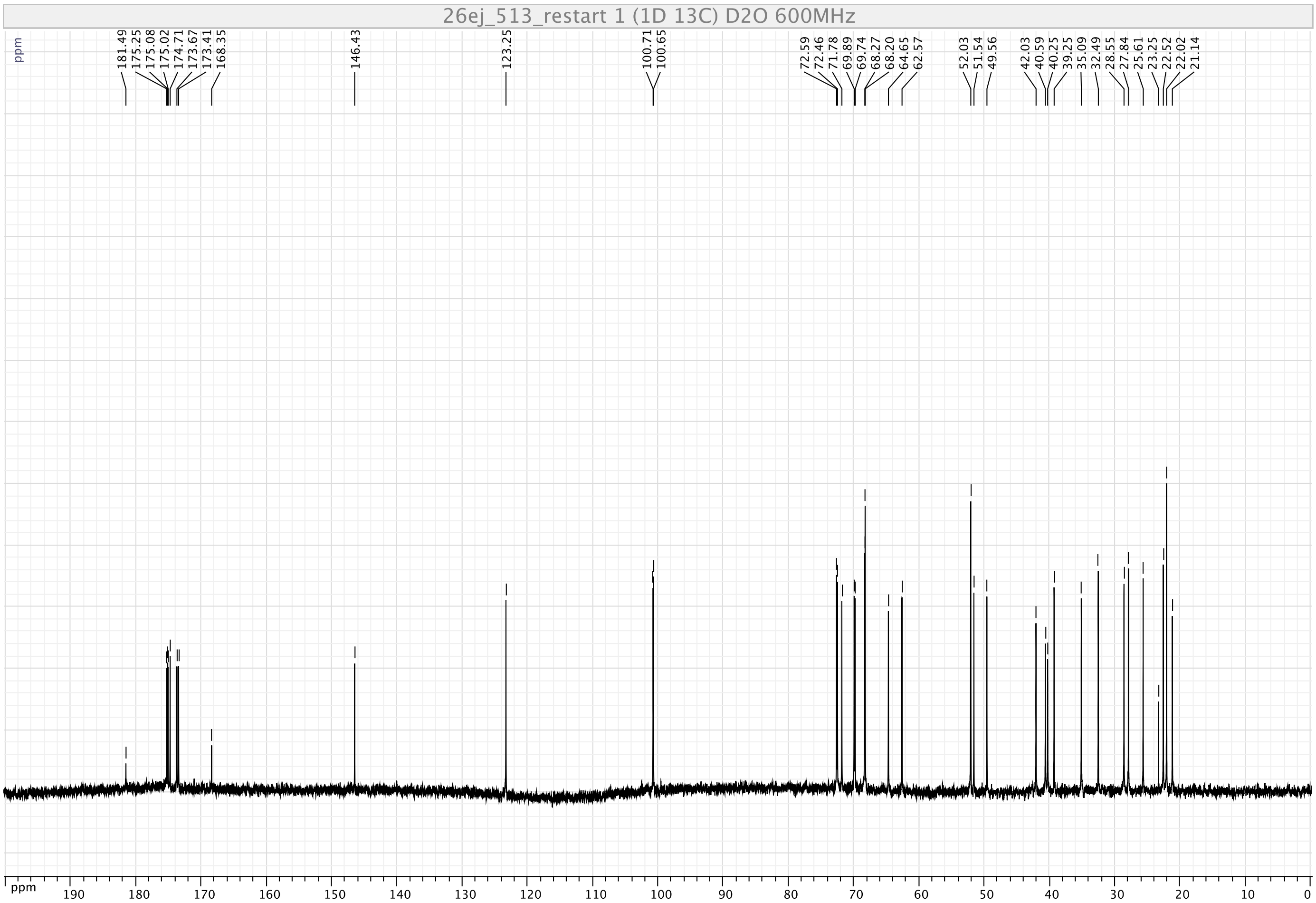

Compound 21 (ME1070)

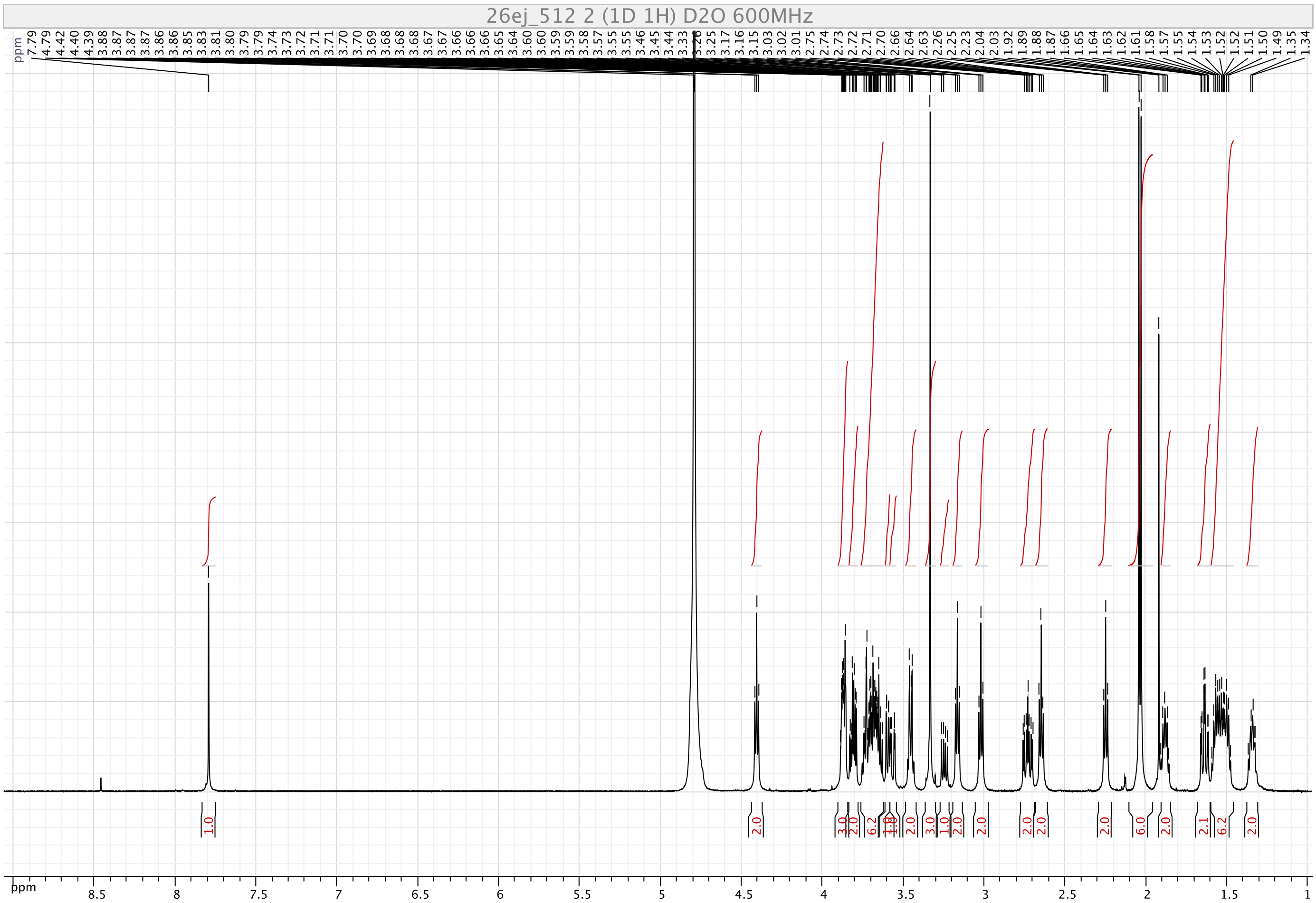

Compound 21 (ME1070)

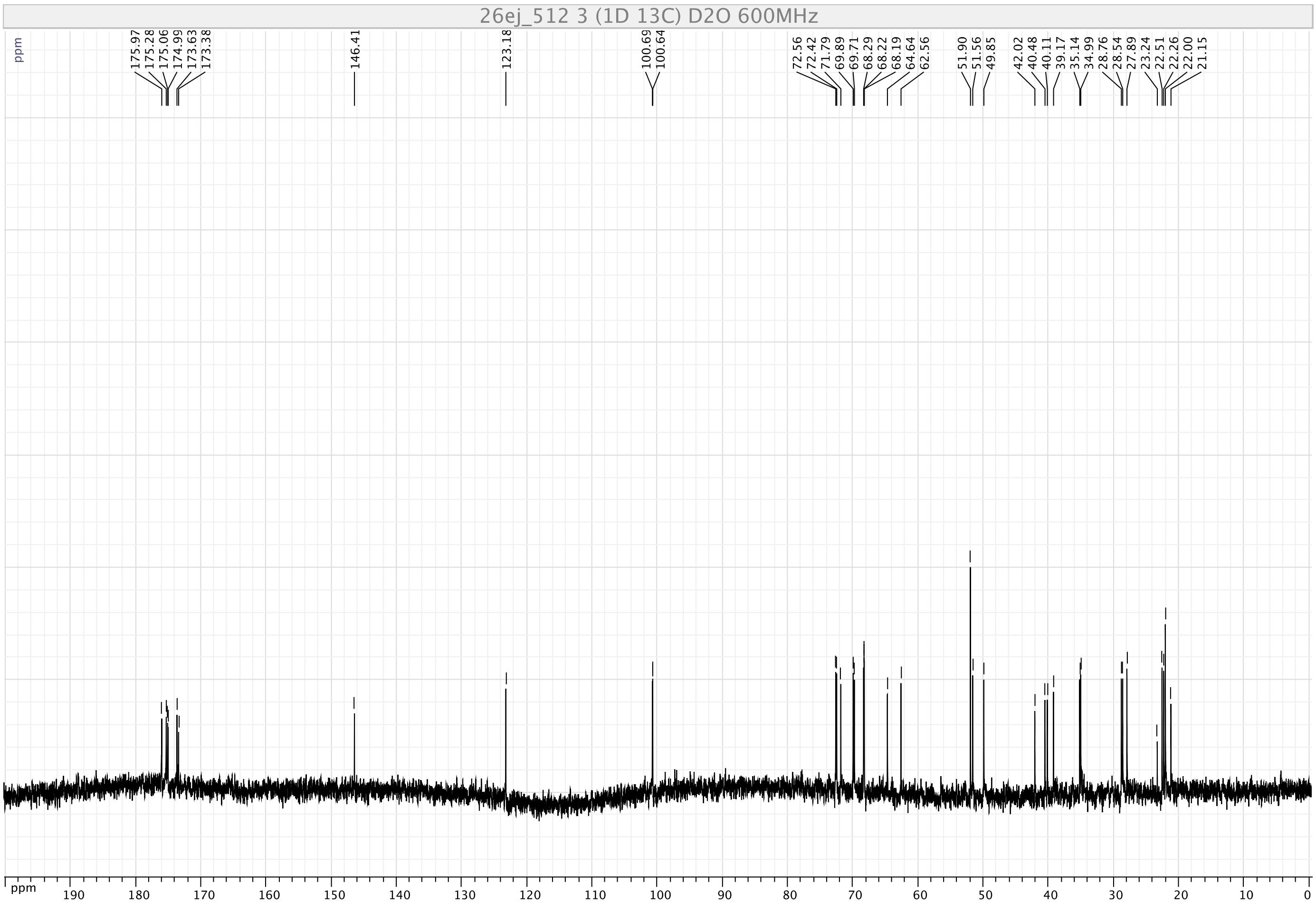

Compound 25

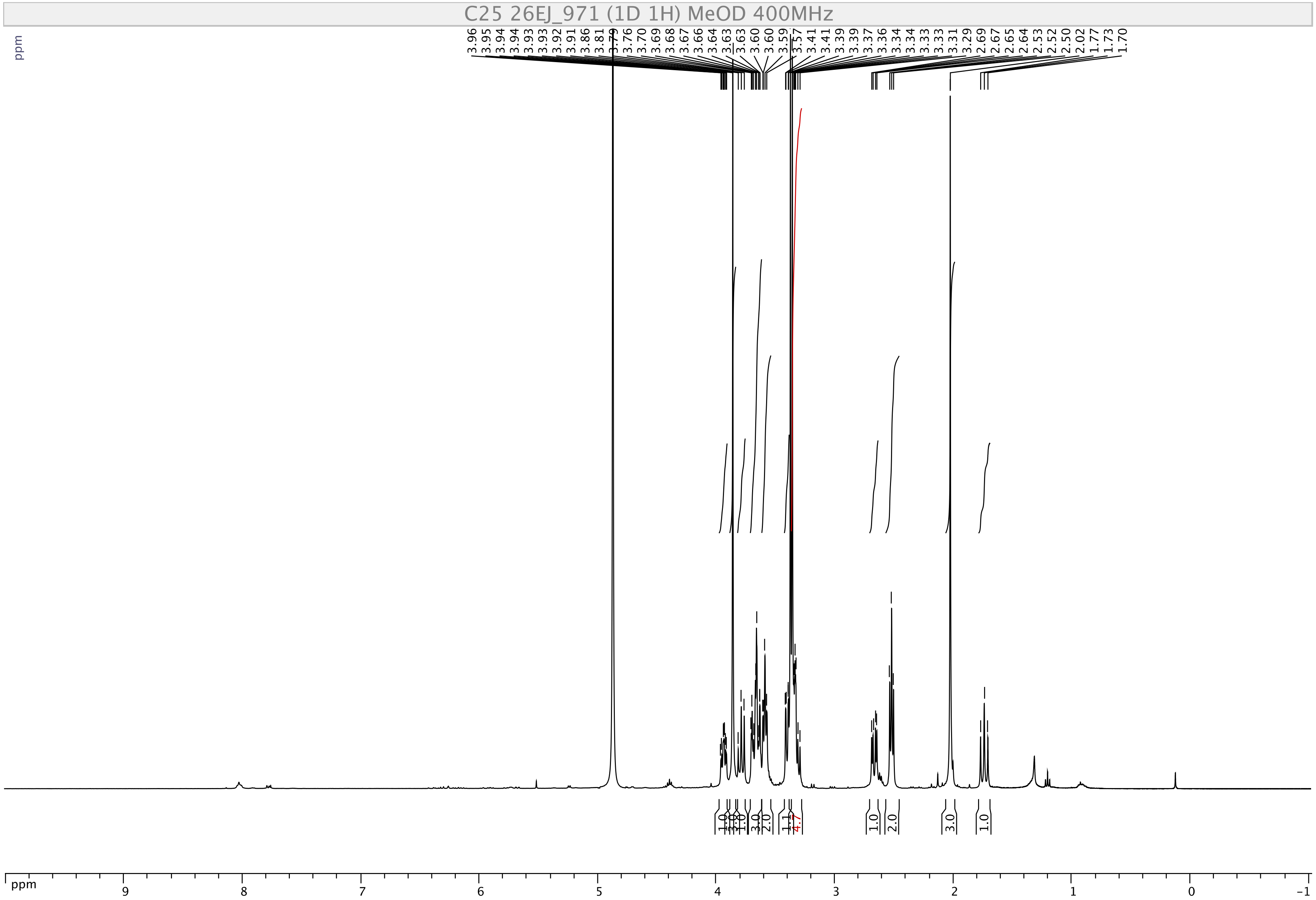

C25 26EJ_971 (1D 1H) MeOD 400MHz
ppm
3.96 3.95 3.94 3.94 3.93 3.93 3.92 3.91 3.86 3.81 3.79 3.76 3.70 3.69 3.68 3.67 3.66 3.64 3.63 3.63 3.60 3.60 3.59 3.57 3.41 3.39 3.39 3.37 3.36 3.34 3.34 3.33 3.33 3.31 3.29 2.69 2.67 2.65 2.64 2.53 2.52 2.50 2.02 1.77 1.73 1.70
1.0 3.0 1.0 3.0 2.0 1.1 4.7 1.0 2.0 3.0 1.0
ppm 9 8 7 6 5 4 3 2 1 0 –1

Compound 25

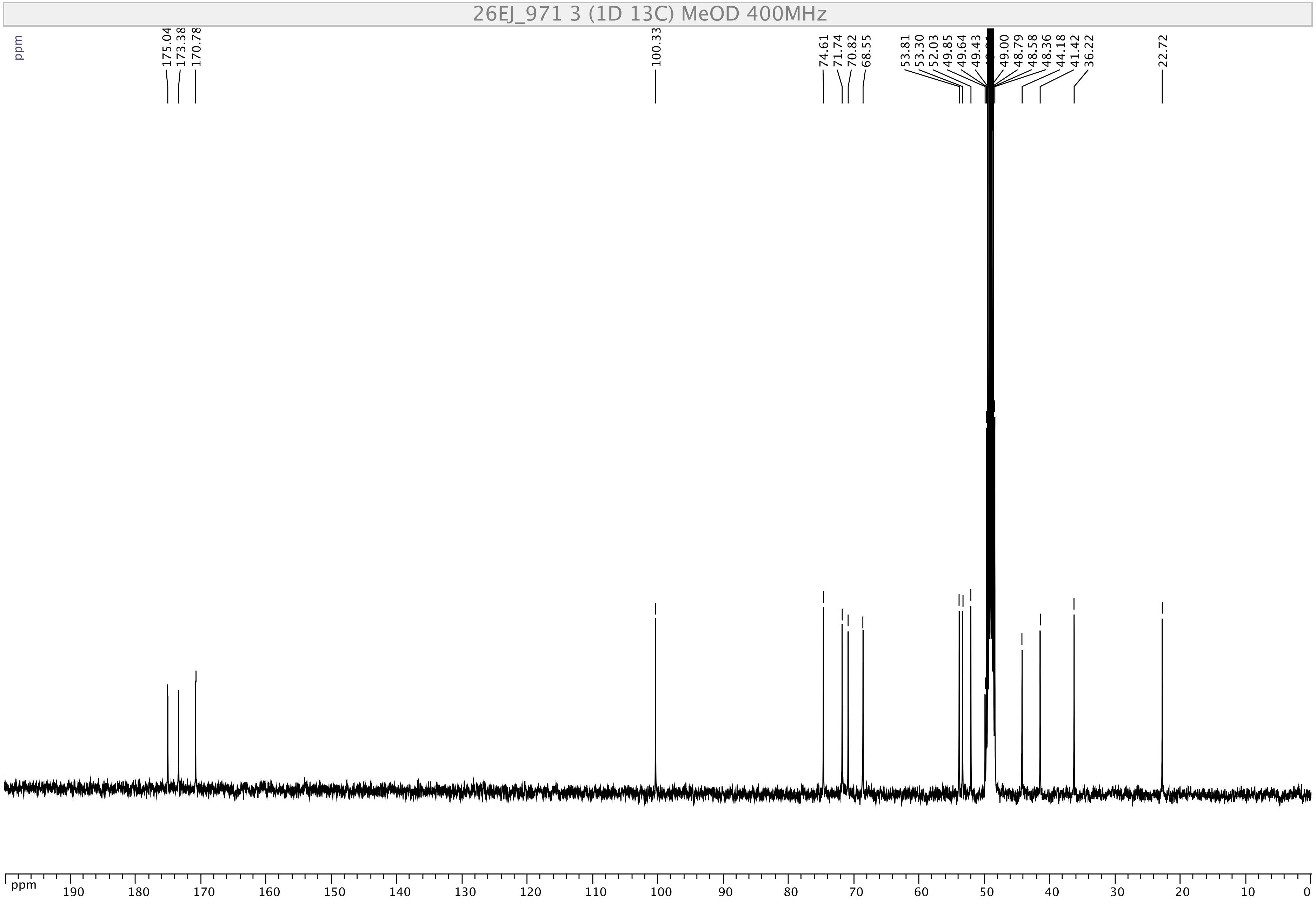
26EJ_971 3 (1D 13C) MeOD 400MHz
ppm
175.04
173.38
170.78
100.33
74.61
71.74
70.82
68.55
53.81
53.30
52.03
49.85
49.64
49.43
49.00
48.79
48.58
48.36
44.18
41.42
36.22
22.72
ppm
190
180
170
160
150
140
130
120
110
100
90
80
70
60
50
40
30
20
10
0

Compound 26

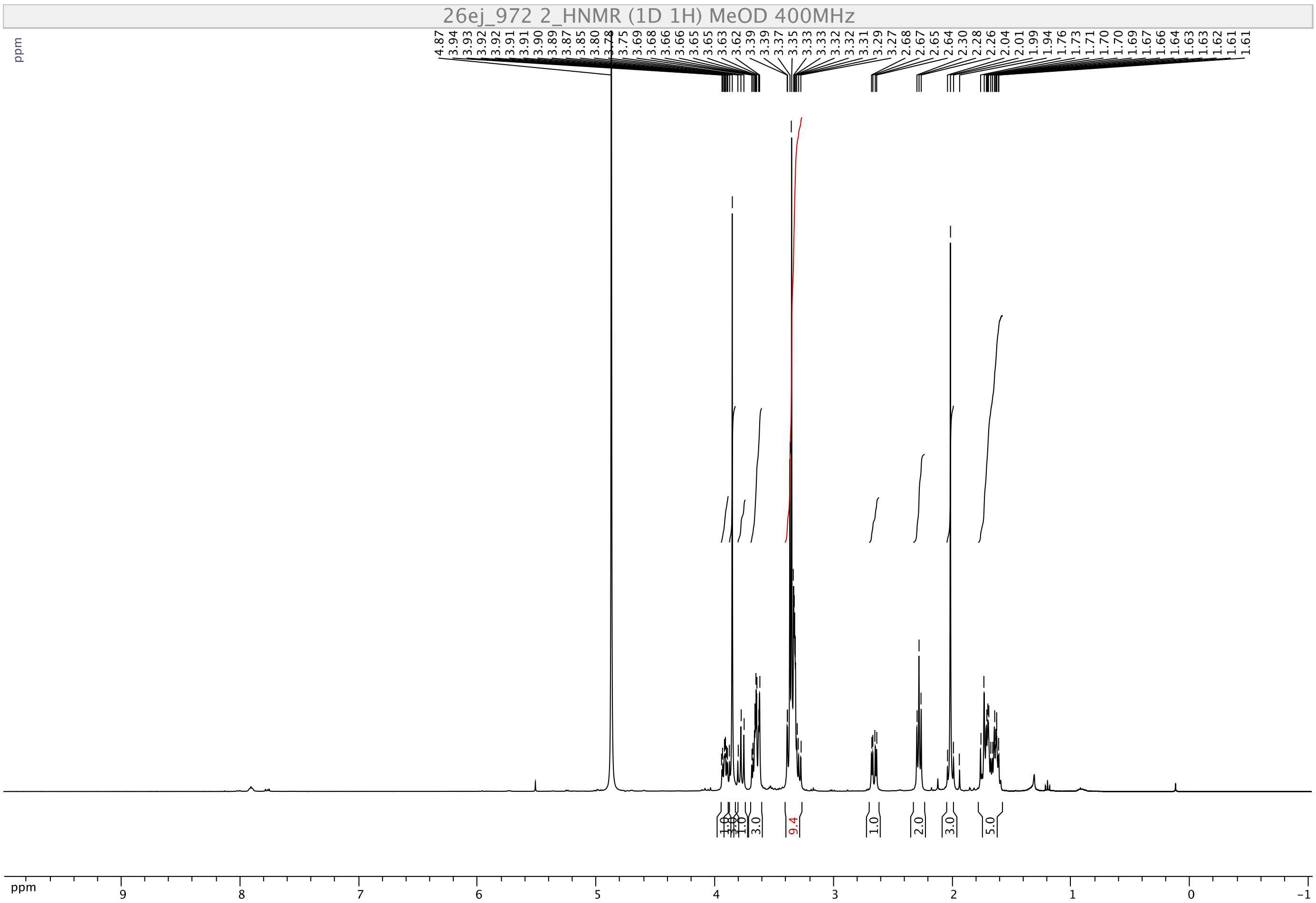
26ej_972 2_HNMR (1D 1H) MeOD 400MHz
4.87
3.94
3.93
3.92
3.92
3.91
3.91
3.90
3.89
3.87
3.85
3.80
3.78
3.75
3.69
3.68
3.66
3.65
3.65
3.63
3.62
3.39
3.39
3.37
3.35
3.33
3.33
3.32
3.32
3.31
3.29
3.27
2.68
2.67
2.65
2.64
2.30
2.28
2.26
2.04
2.01
1.99
1.94
1.76
1.73
1.71
1.70
1.70
1.69
1.67
1.66
1.64
1.63
1.62
1.61
1.61
9.4
1.0
2.0
3.0
5.0
3.0
1.0
ppm
9
8
7
6
5
4
3
2
1
0
–1

Compound 26

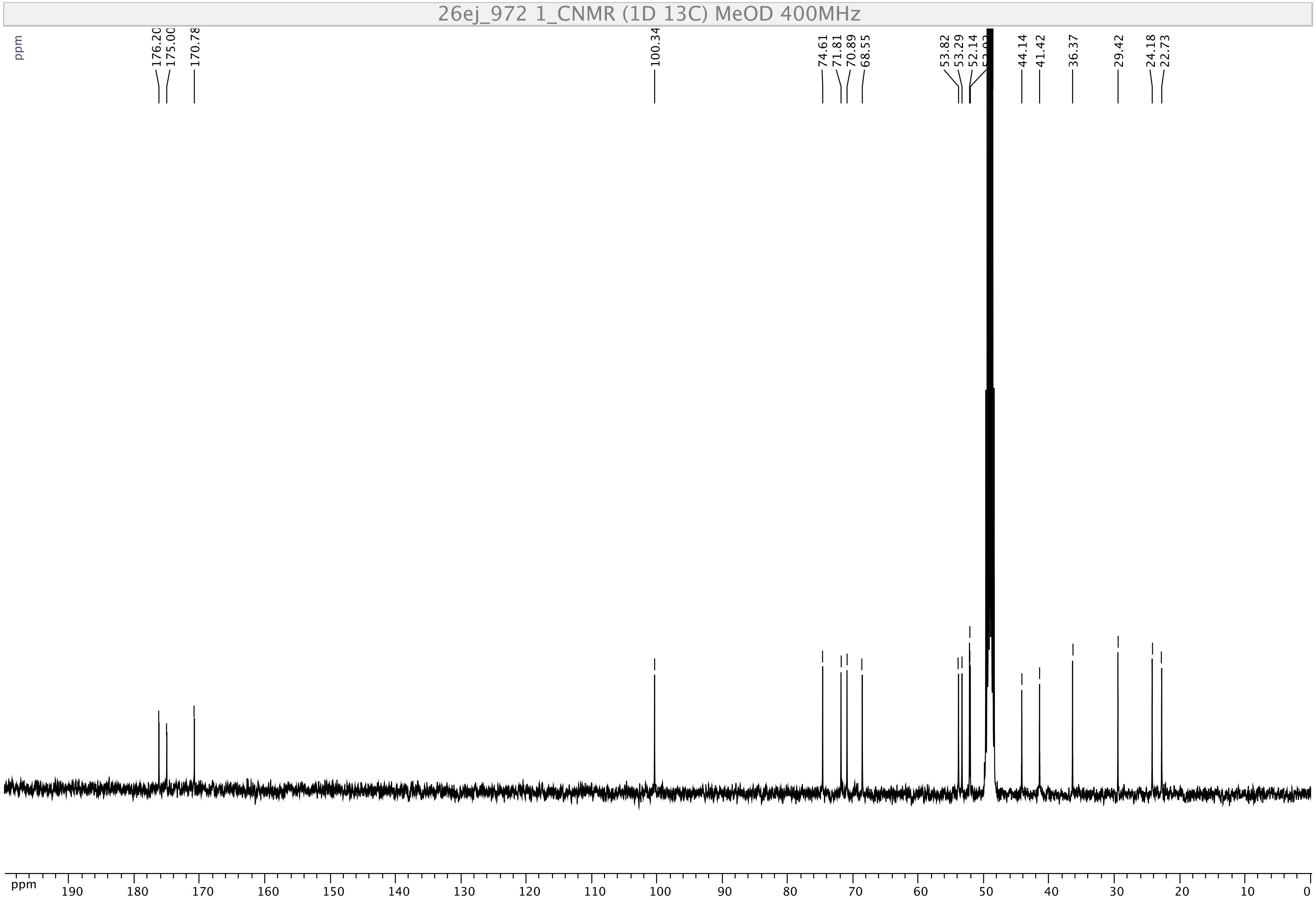
26ej_972 1_CNMR (1D 13C) MeOD 400MHz
ppm
176.20
175.00
170.78
100.34
74.61
71.81
70.89
68.55
53.82
53.29
52.14
44.14
41.42
36.37
29.42
24.18
22.73
ppm
190
180
170
160
150
140
130
120
110
100
90
80
70
60
50
40
30
20
10
0

Compound 27

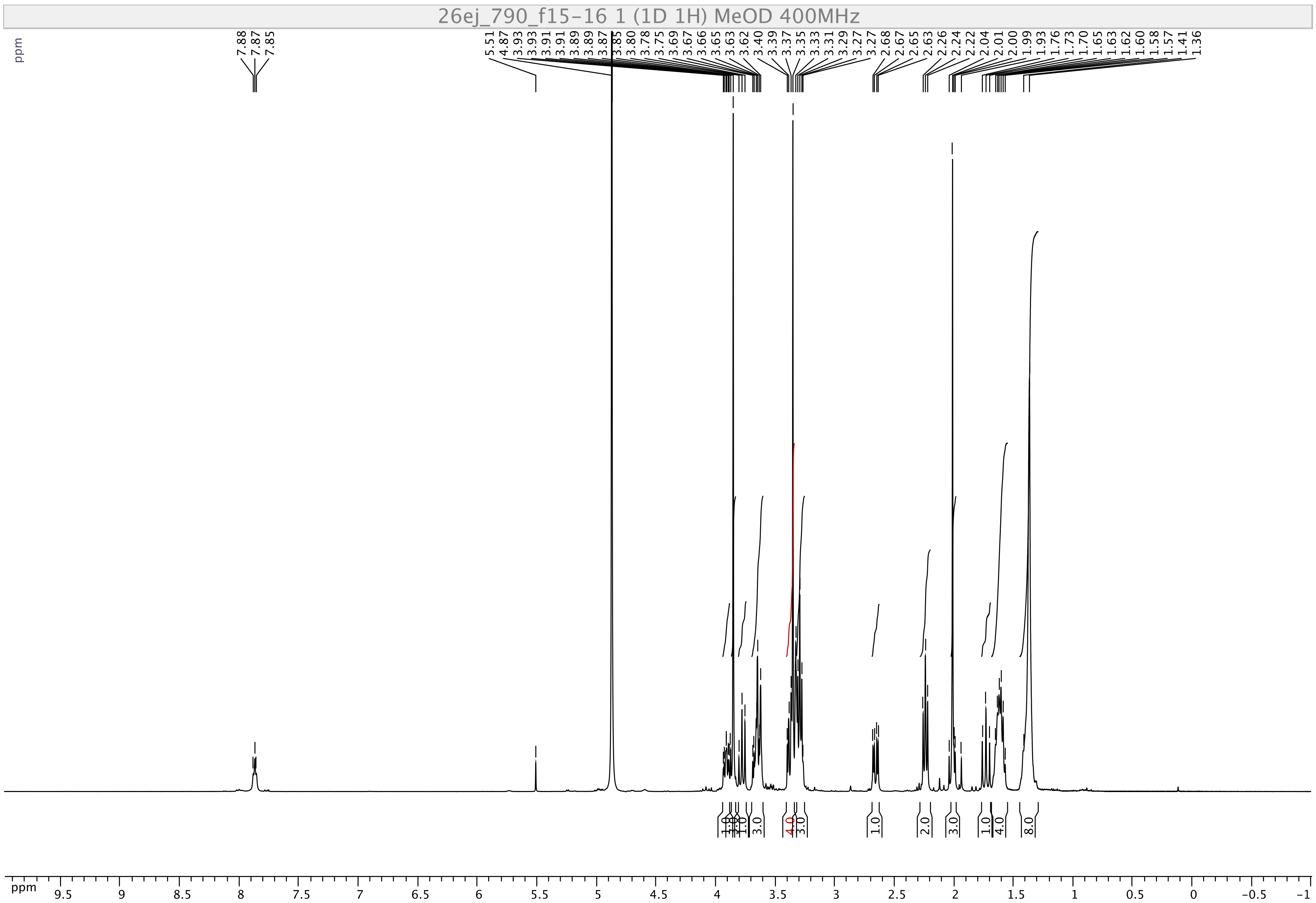
26ej_790_f15-16 1 (1D 1H) MeOD 400MHz
ppm
7.88
7.87
7.85
5.51
4.87
3.93
3.93
3.91
3.91
3.89
3.89
3.87
3.85
3.80
3.78
3.75
3.69
3.67
3.66
3.65
3.63
3.62
3.40
3.39
3.37
3.35
3.33
3.31
3.29
3.27
3.27
2.68
2.67
2.65
2.63
2.26
2.24
2.22
2.04
2.01
2.00
1.99
1.93
1.76
1.73
1.70
1.65
1.63
1.62
1.60
1.58
1.57
1.41
1.36
3.0
1.0
2.0
4.0
3.0
1.0
2.0
3.0
1.0
4.0
8.0
ppm
9.5
9
8.5
8
7.5
7
6.5
6
5.5
5
4.5
4
3.5
3
2.5
2
1.5
1
0.5
0
−0.5
−1

Compound 27

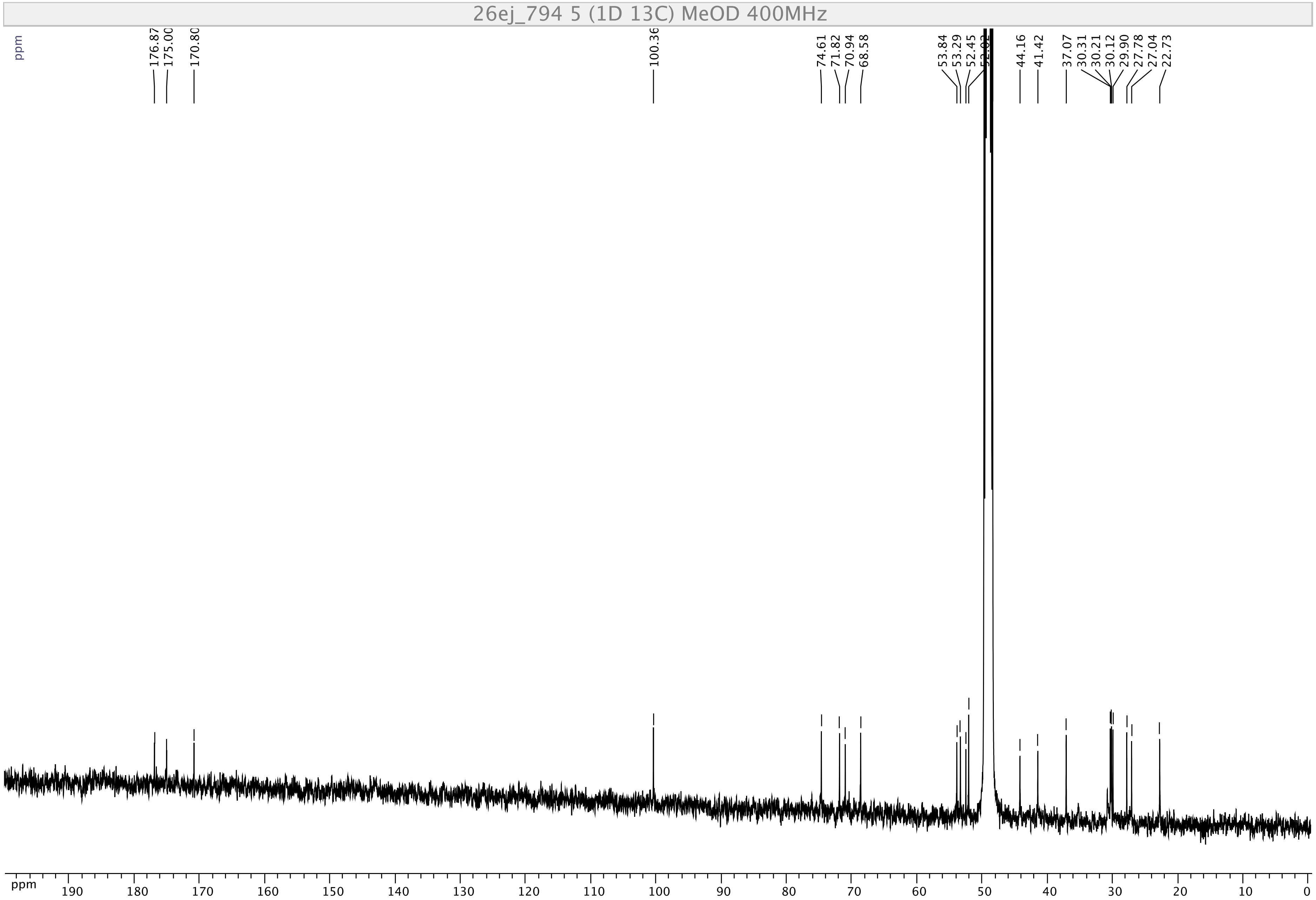

26ej_794 5 (1D 13C) MeOD 400MHz
ppm
176.87
175.00
170.80
100.36
74.61
71.82
70.94
68.58
53.84
53.29
52.45
44.16
41.42
37.07
30.31
30.21
30.12
29.90
27.78
27.04
22.73
ppm
190
180
170
160
150
140
130
120
110
100
90
80
70
60
50
40
30
20
10
0

Compound 29

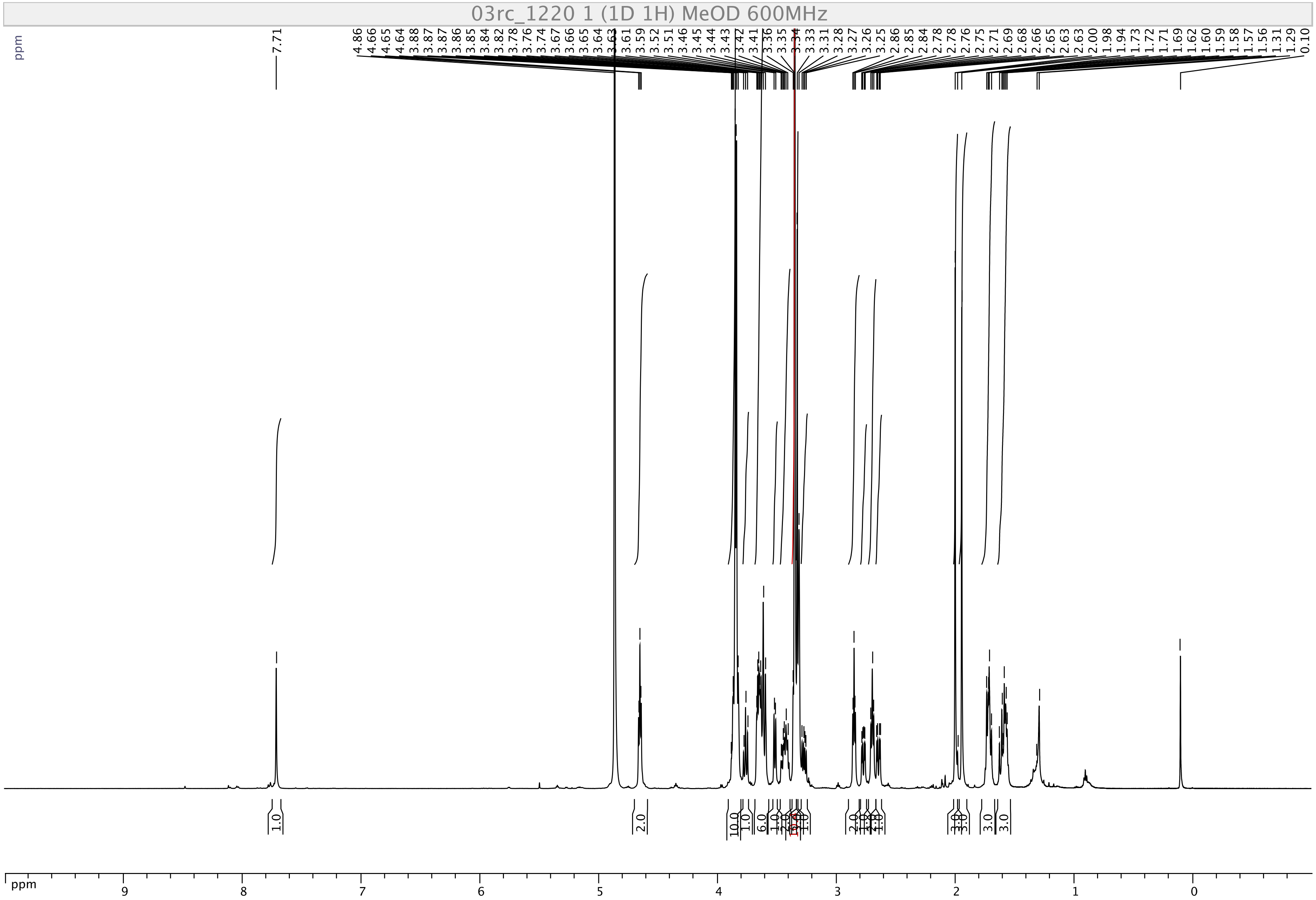

Compound 29

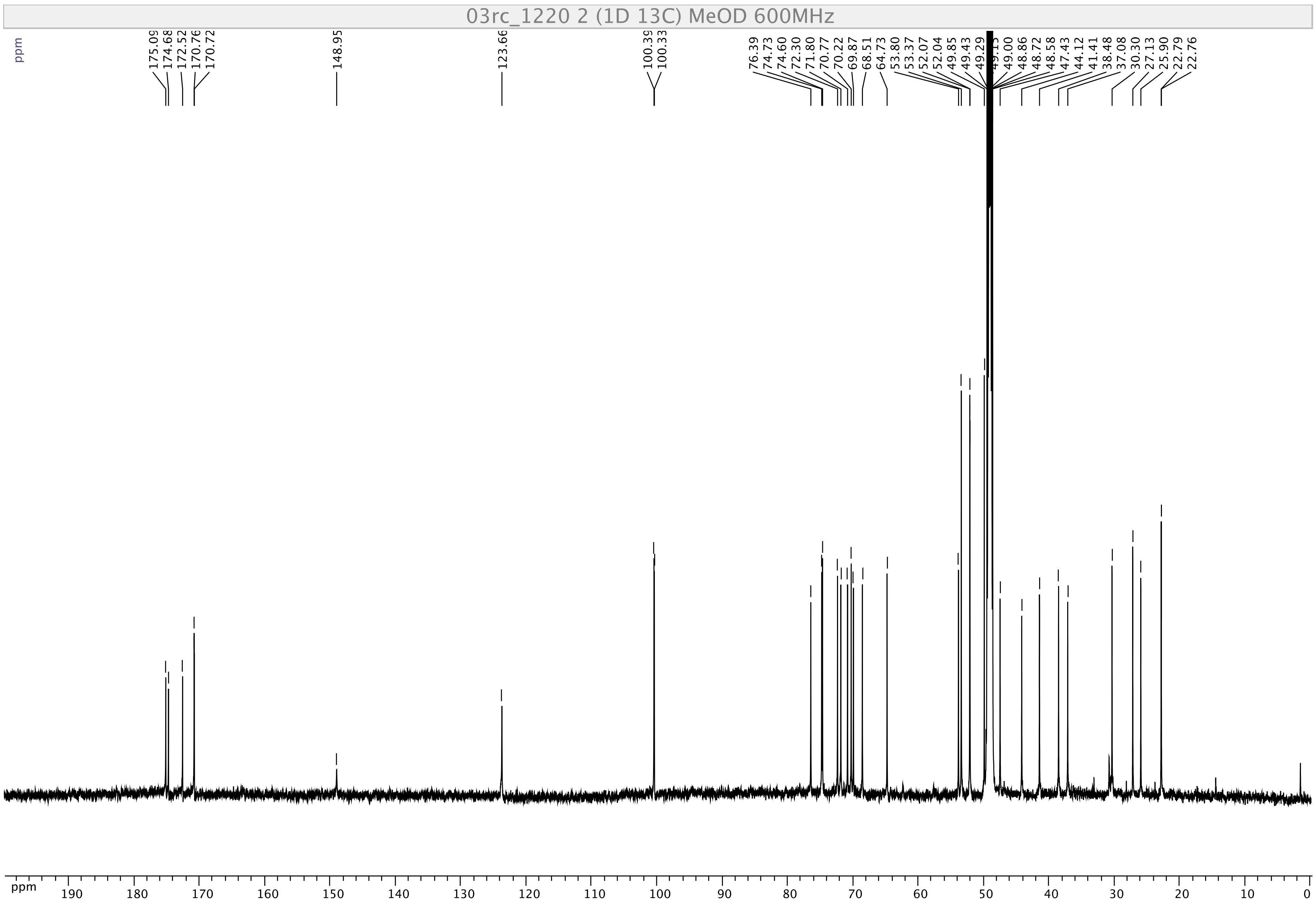
03rc_1220 2 (1D 13C) MeOD 600MHz
ppm
175.09
174.68
172.52
170.76
170.72
148.95
123.66
100.39
100.33
76.39
74.73
74.60
72.30
71.80
70.77
70.22
69.87
68.51
64.73
53.80
53.37
52.07
52.04
49.85
49.43
49.29
49.00
48.86
48.72
48.58
47.43
44.12
41.41
38.48
37.08
30.30
27.13
25.90
22.79
22.76
ppm
190
180
170
160
150
140
130
120
110
100
90
80
70
60
50
40
30
20
10
0

Compound 30

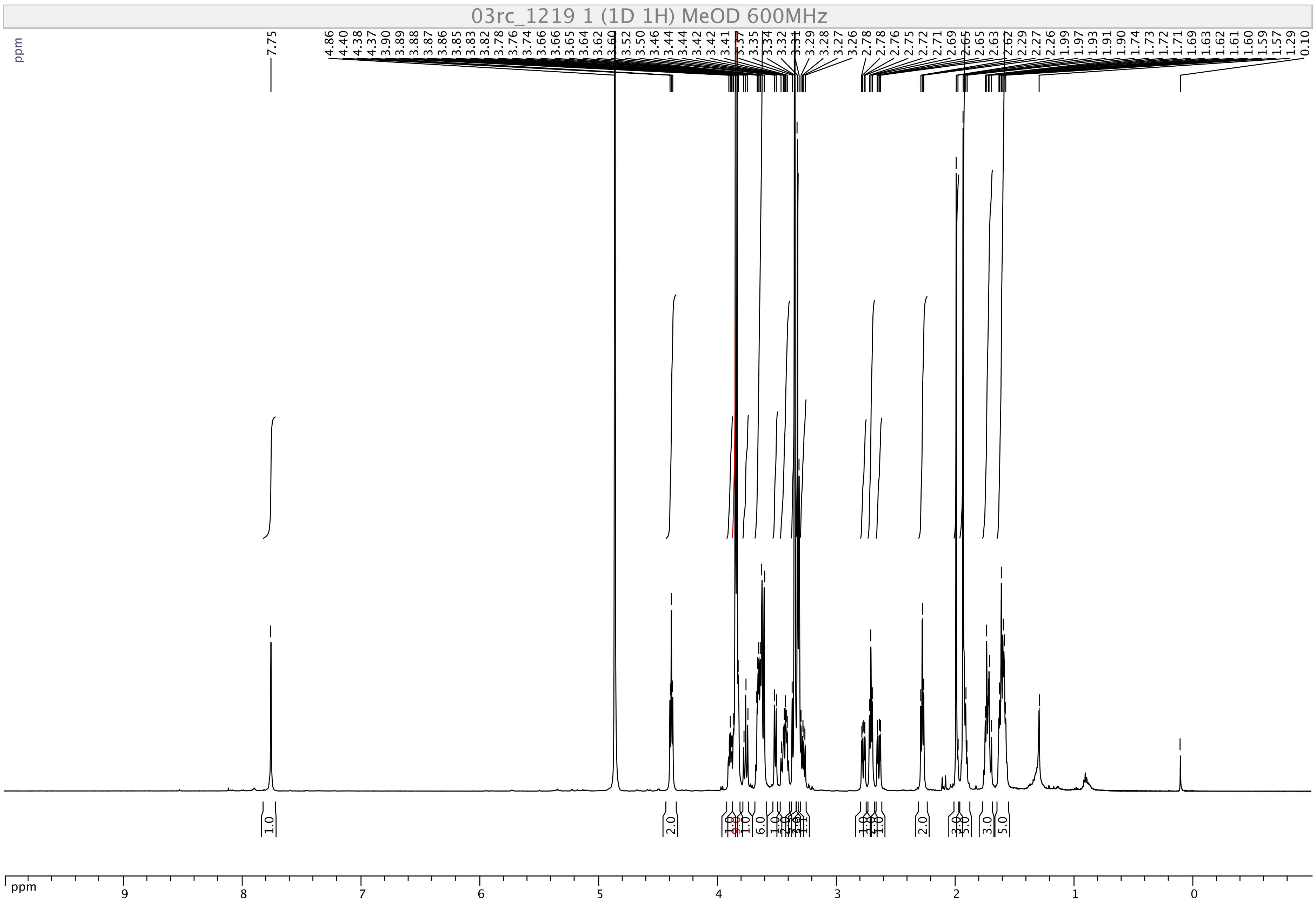

Compound 30

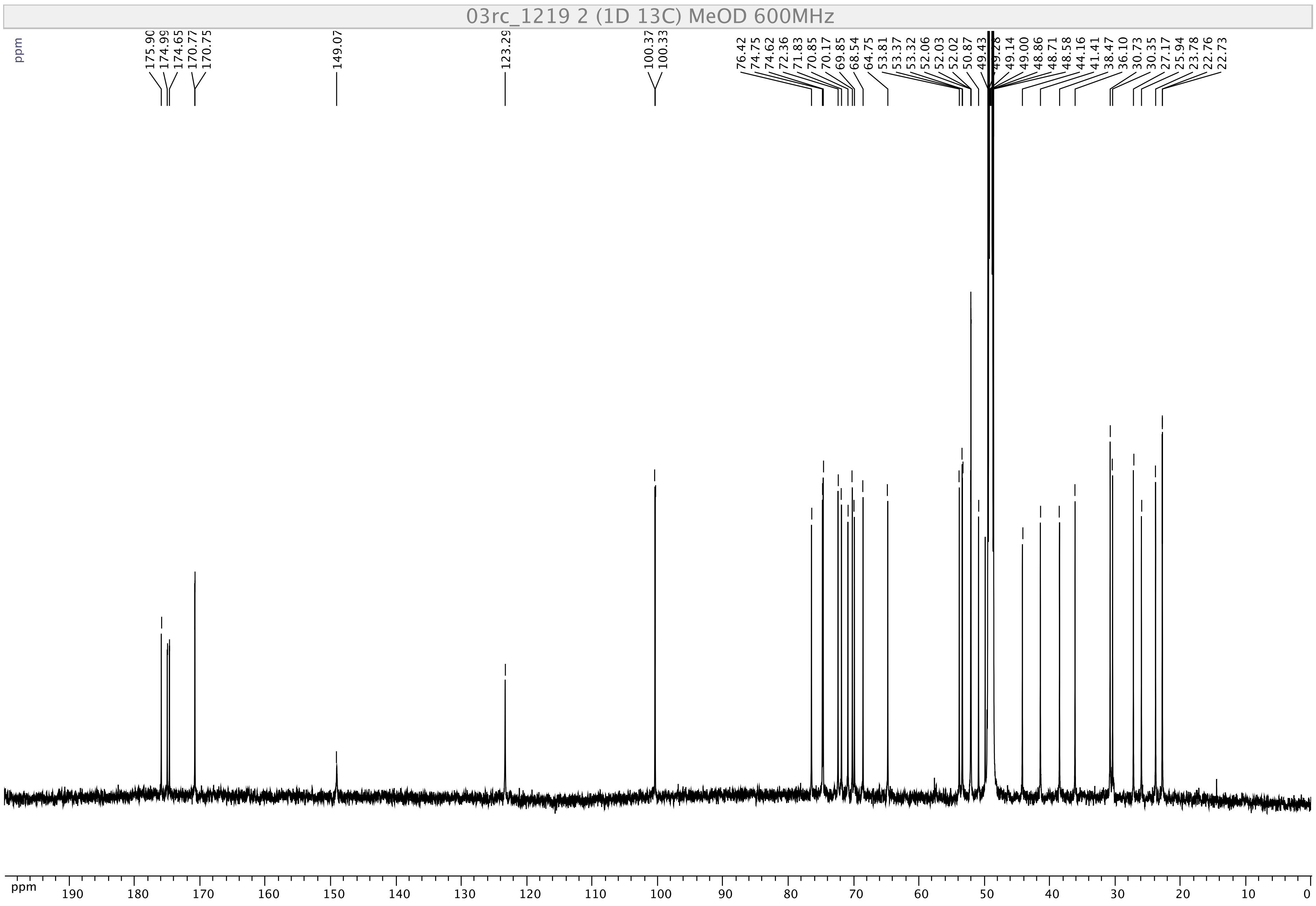
03rc_1219 2 (1D 13C) MeOD 600MHz
ppm
175.90
174.99
174.65
170.77
170.75
149.07
123.29
100.37
100.33
76.42
74.75
74.62
72.36
71.83
70.85
70.17
69.85
68.54
64.75
53.81
53.37
53.32
52.06
52.03
52.02
50.87
49.43
49.28
49.14
49.00
48.86
48.71
48.58
44.16
41.41
38.47
36.10
30.73
30.35
27.17
25.94
23.78
22.76
22.73
ppm
190
180
170
160
150
140
130
120
110
100
90
80
70
60
50
40
30
20
10
0

Compound 31

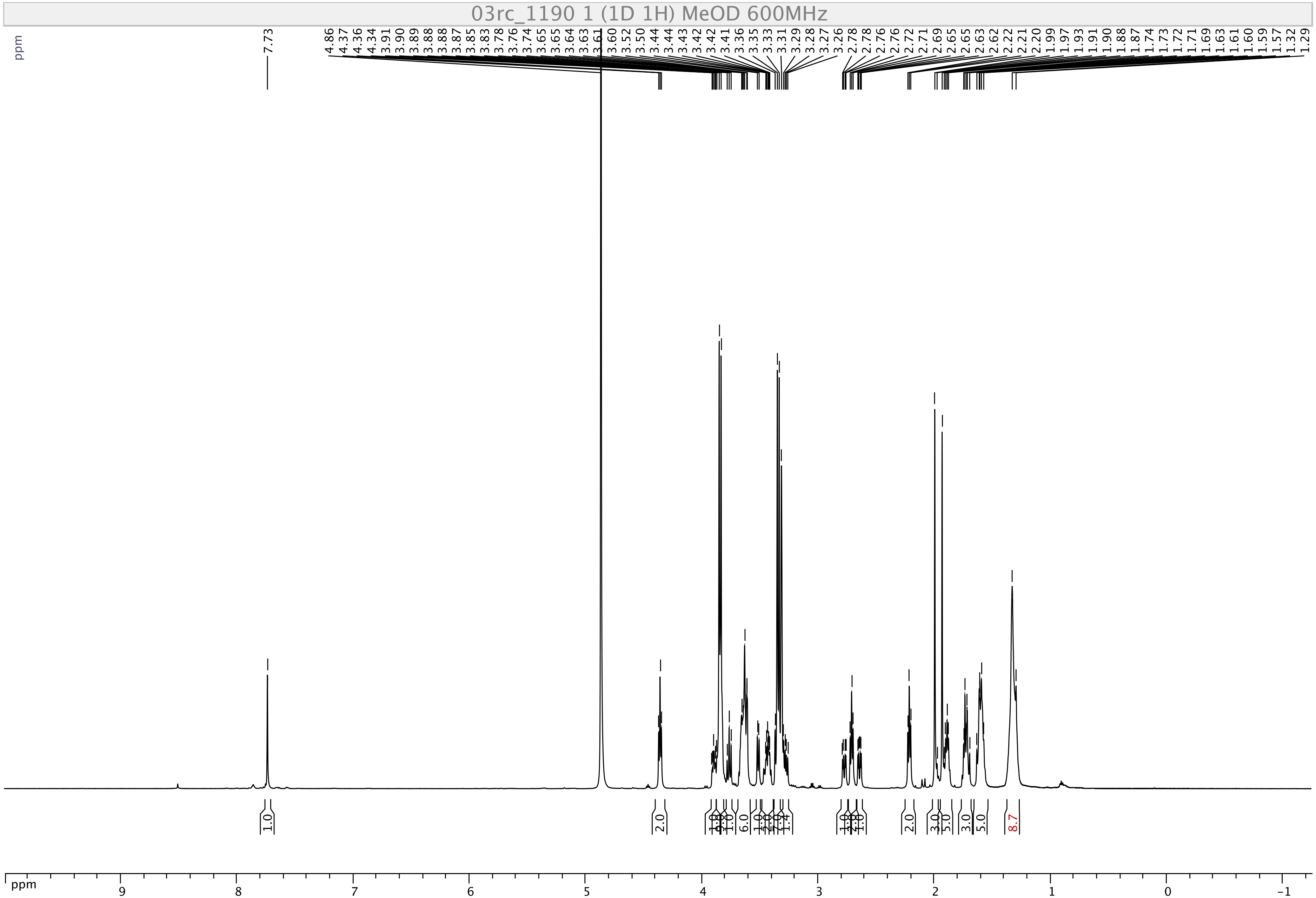

Compound 31

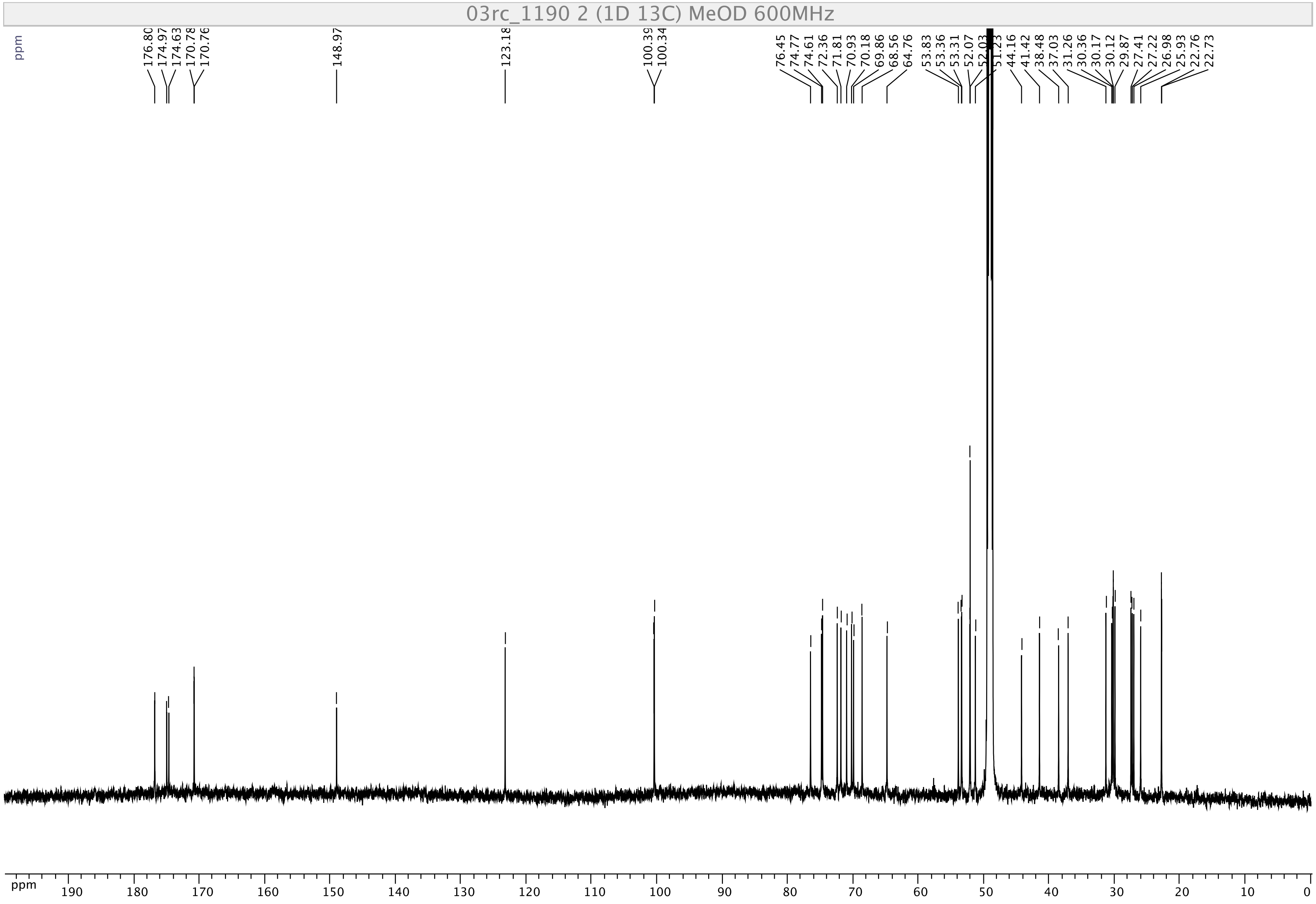
03rc_1190 2 (1D 13C) MeOD 600MHz
ppm
176.80
174.97
174.63
170.78
170.76
148.97
123.18
100.39
100.34
76.45
74.77
74.61
72.36
71.81
70.93
70.18
69.86
68.56
64.76
53.83
53.36
53.31
52.07
52.03
51.23
44.16
41.42
38.48
37.03
31.26
30.36
30.17
30.12
29.87
27.41
27.22
26.98
25.93
22.76
22.73
ppm
190
180
170
160
150
140
130
120
110
100
90
80
70
60
50
40
30
20
10
0

Compound 32 (ME1147)

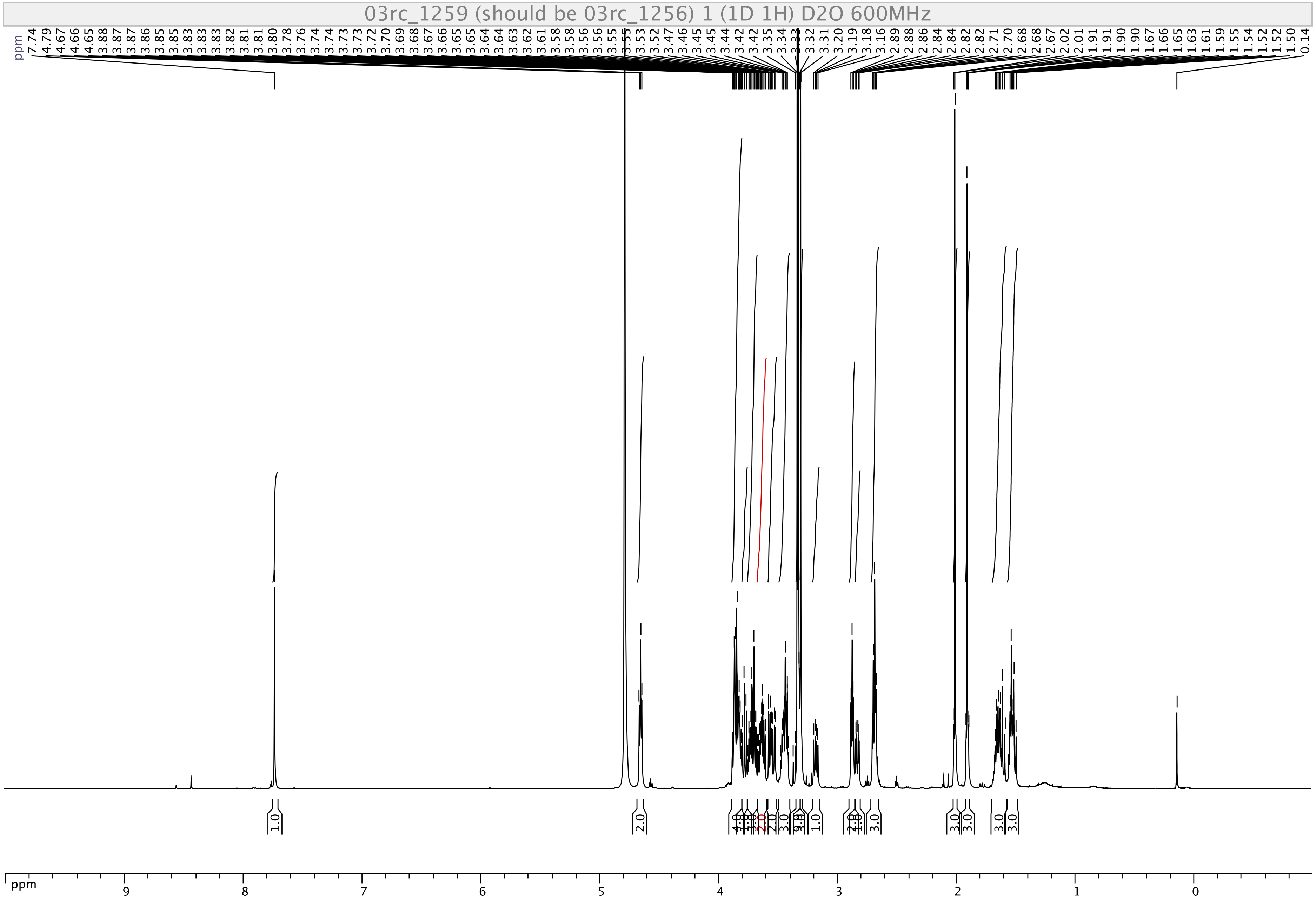

Compound 32 (ME1147)

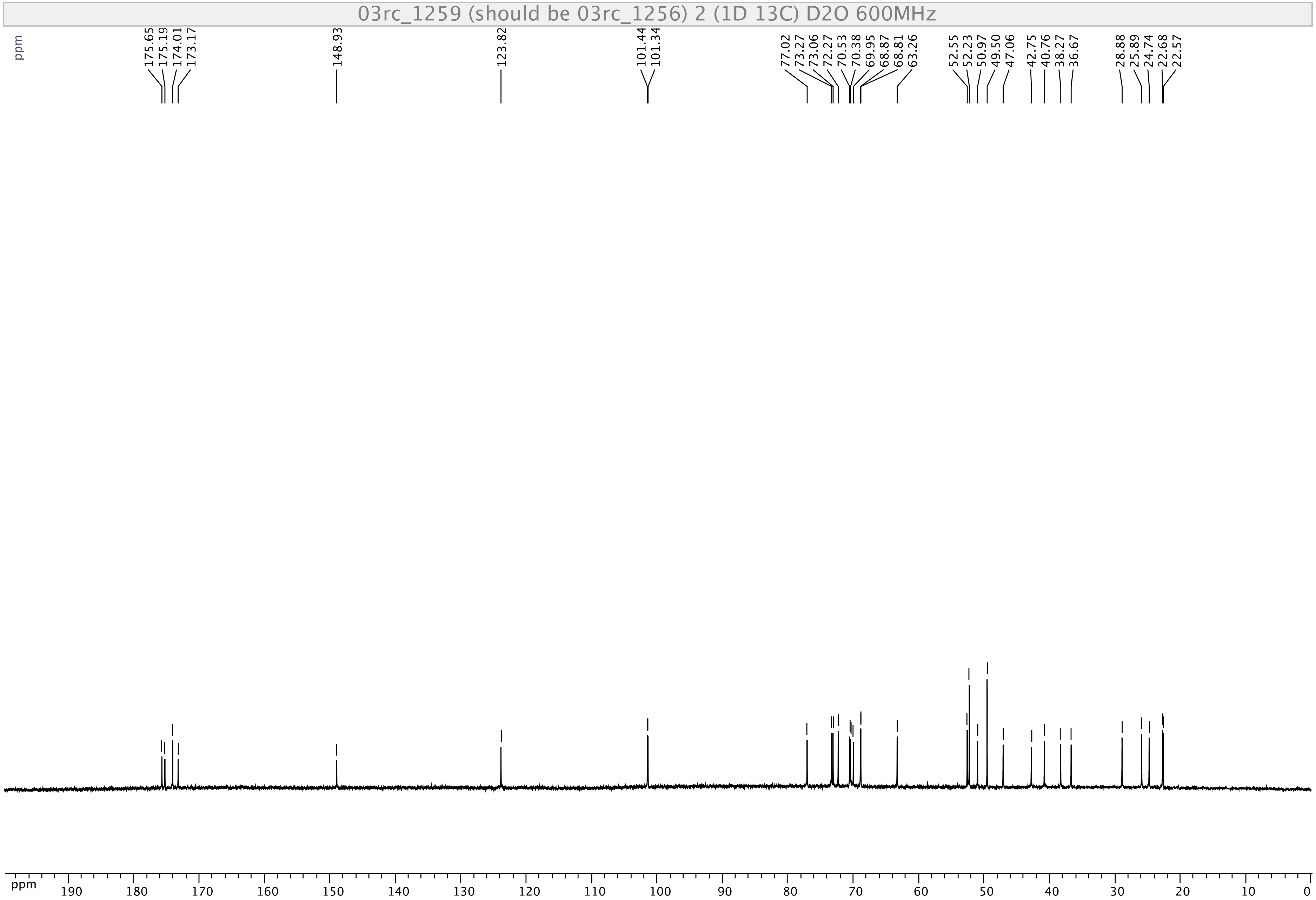

Compound 33 (ME1148)

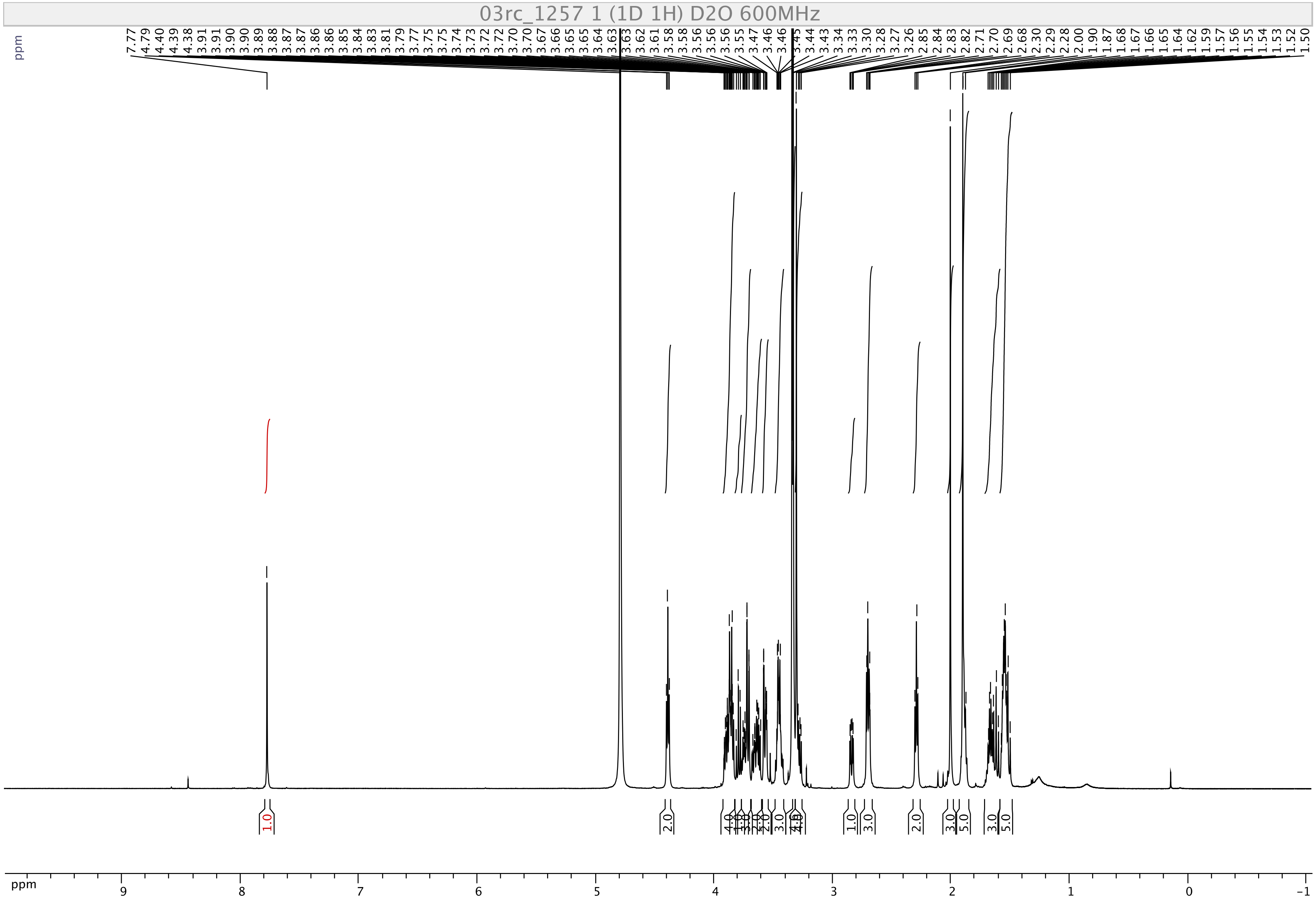

Compound 33 (ME1148)

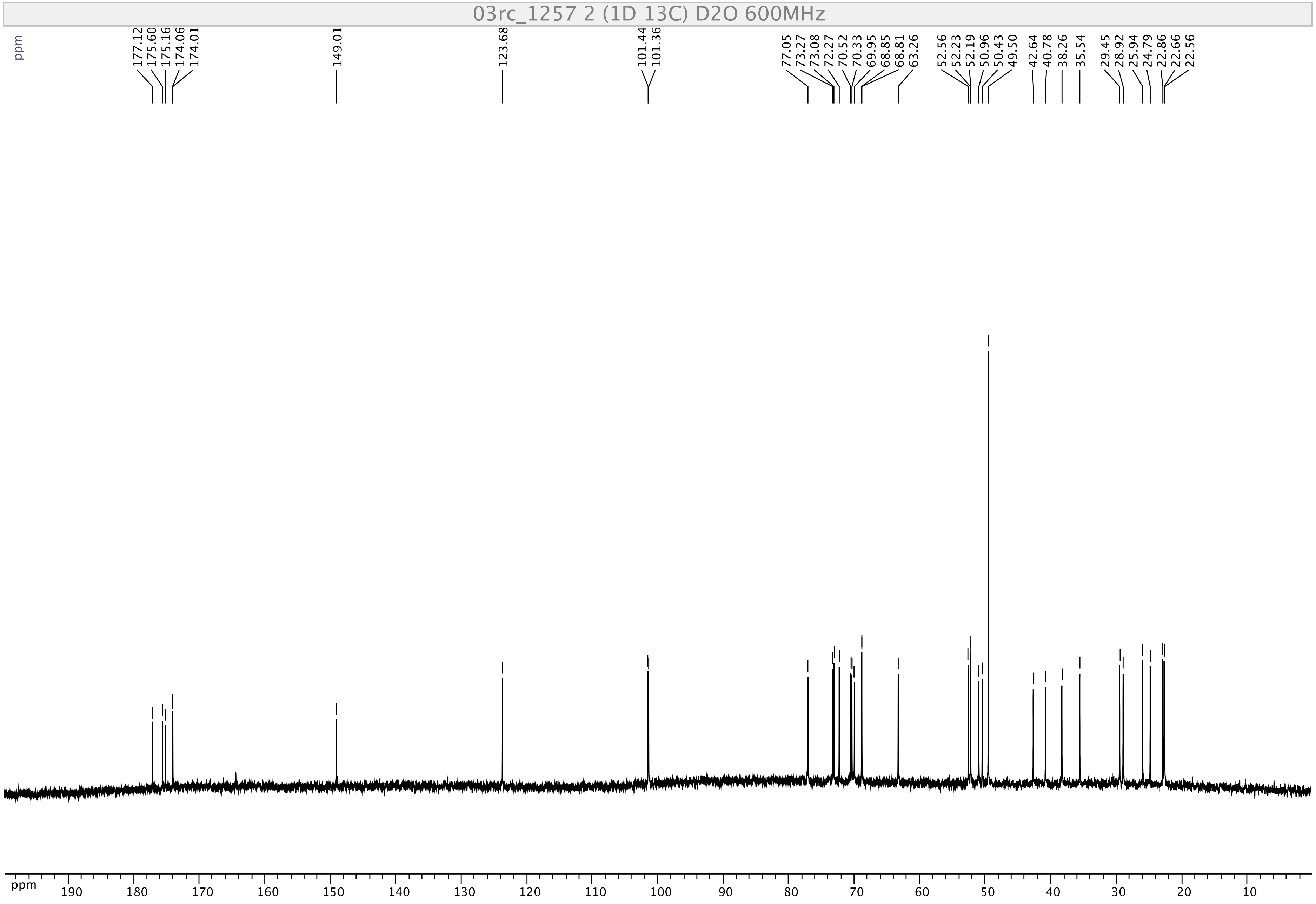

Compound 34 (ME1149)

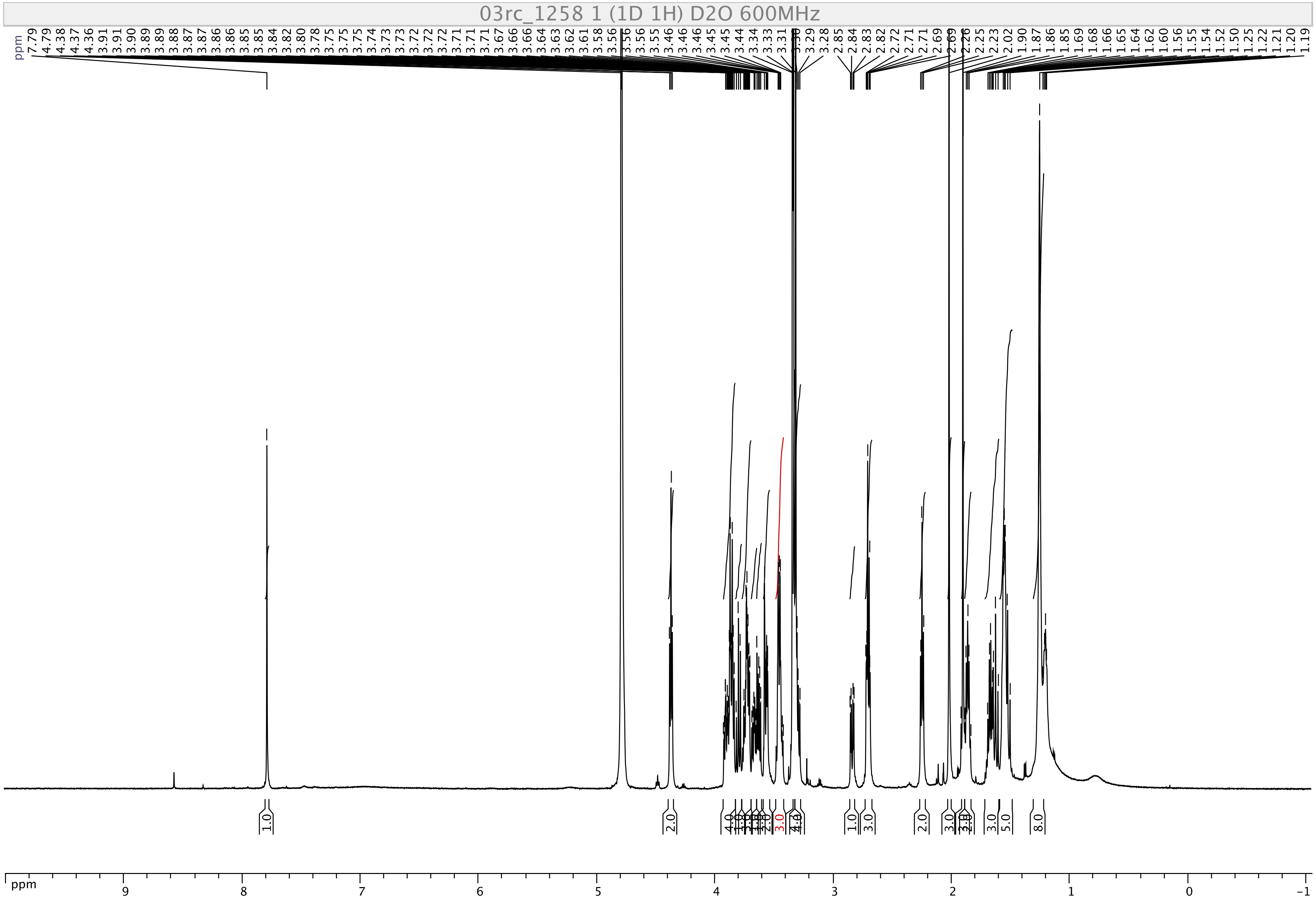

Compound 34 (ME1149)

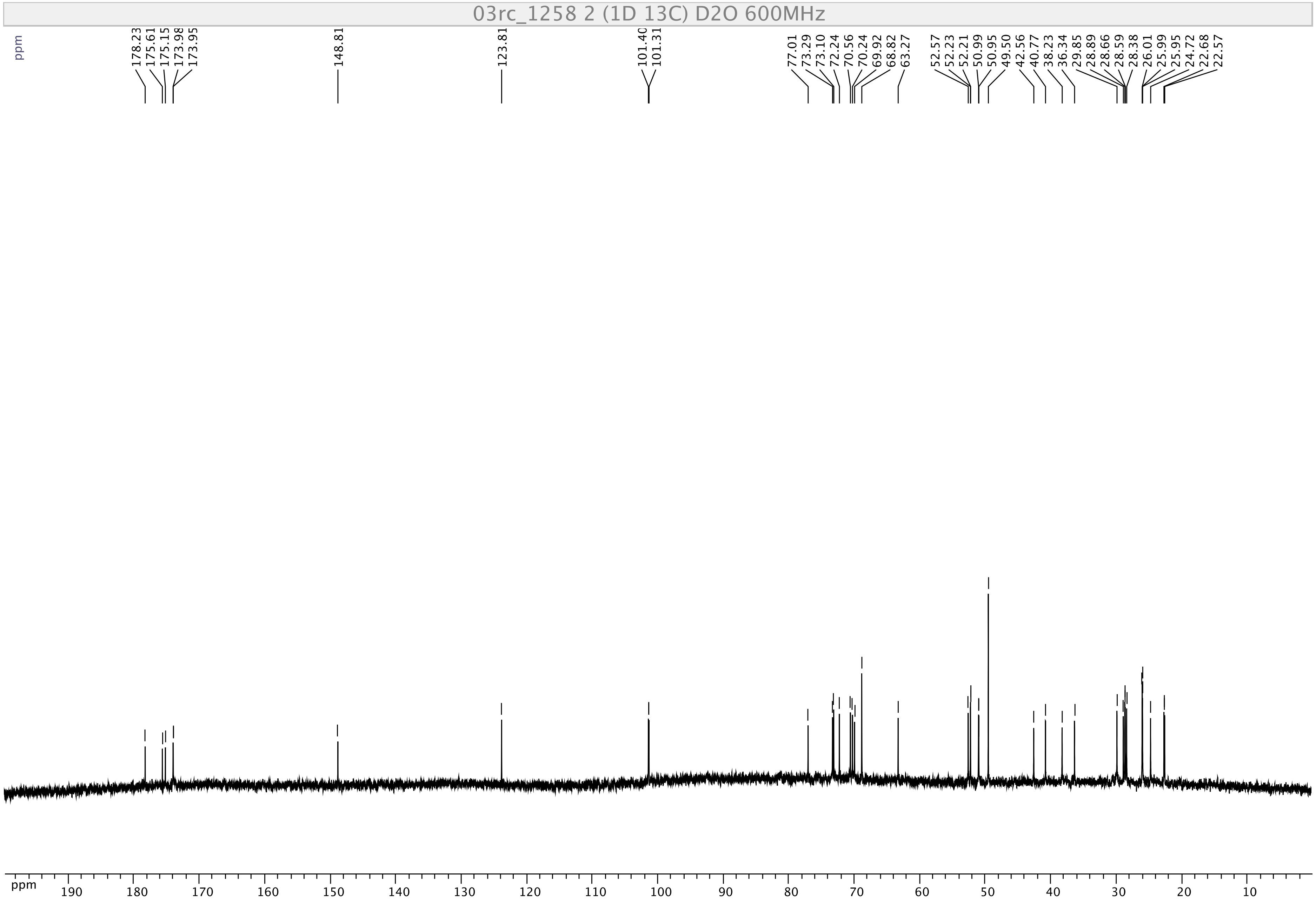

Compound 38

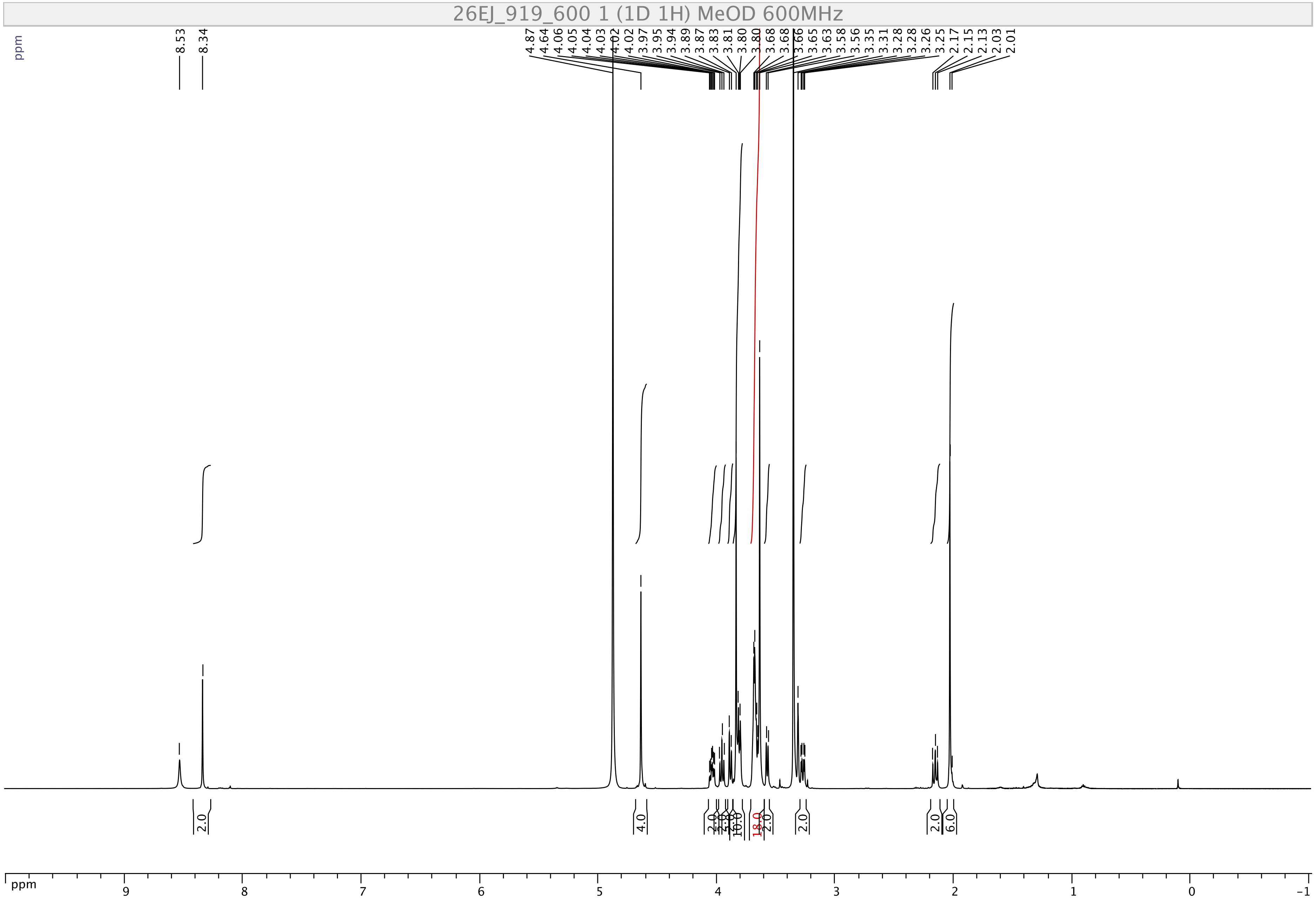
26EJ_919_600 1 (1D 1H) MeOD 600MHz
ppm
8.53
8.34
4.87
4.64
4.06
4.05
4.04
4.03
4.02
4.02
3.97
3.95
3.94
3.89
3.87
3.83
3.81
3.80
3.80
3.68
3.66
3.65
3.63
3.58
3.56
3.35
3.31
3.28
3.28
3.26
3.25
2.17
2.15
2.13
2.03
2.01
2.0
4.0
2.0
2.0
2.0
10.0
18.0
2.0
2.0
2.0
6.0
ppm
9
8
7
6
5
4
3
2
1
0
–1

Compound 38

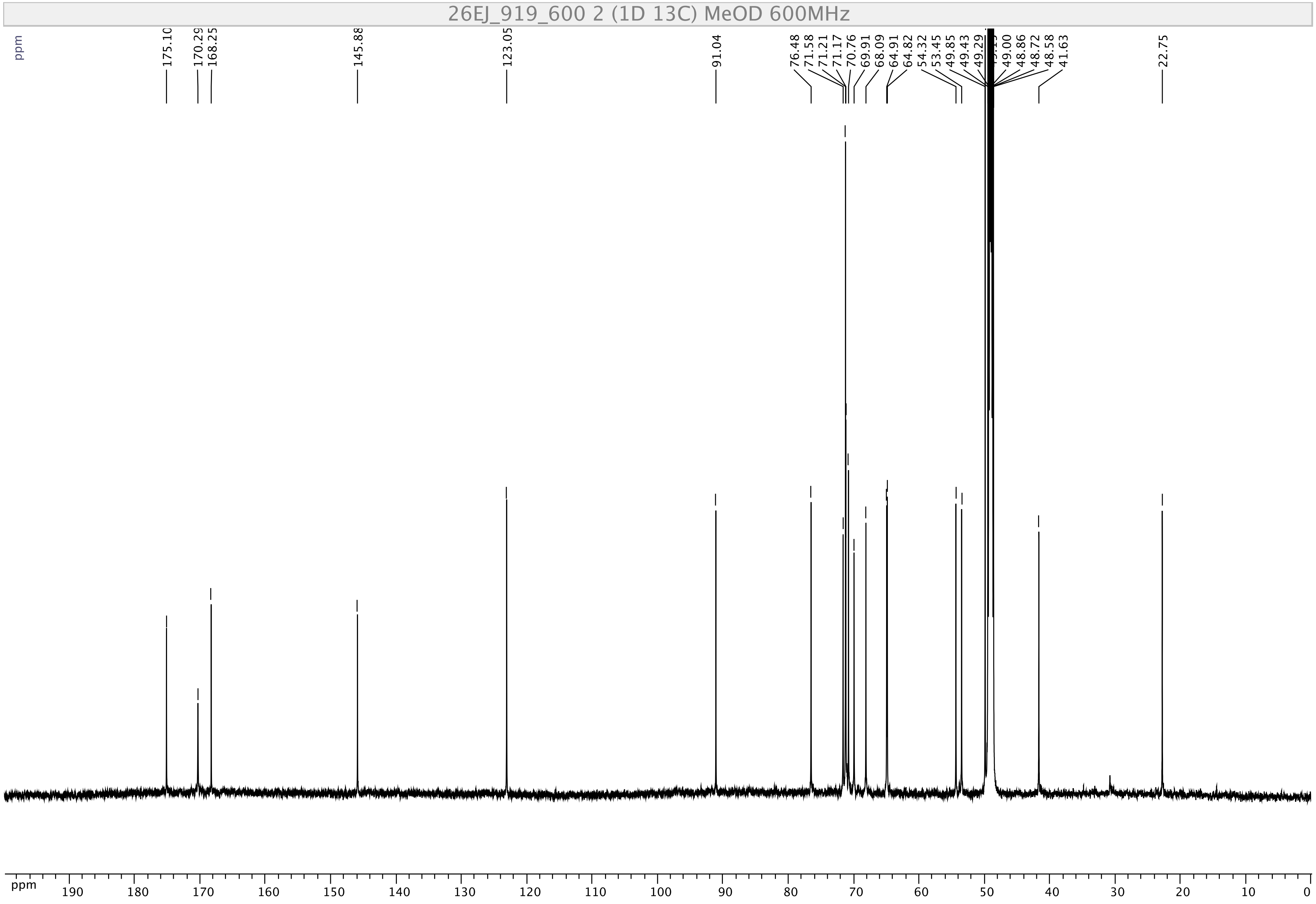
26EJ_919_600 2 (1D 13C) MeOD 600MHz
ppm
175.10
170.29
168.25
145.88
123.05
91.04
76.48
71.58
71.21
71.17
70.76
69.91
68.09
64.91
64.82
54.32
53.45
49.85
49.43
49.29
49.00
48.86
48.72
48.58
41.63
22.75
ppm
190
180
170
160
150
140
130
120
110
100
90
80
70
60
50
40
30
20
10
0

Compound 39

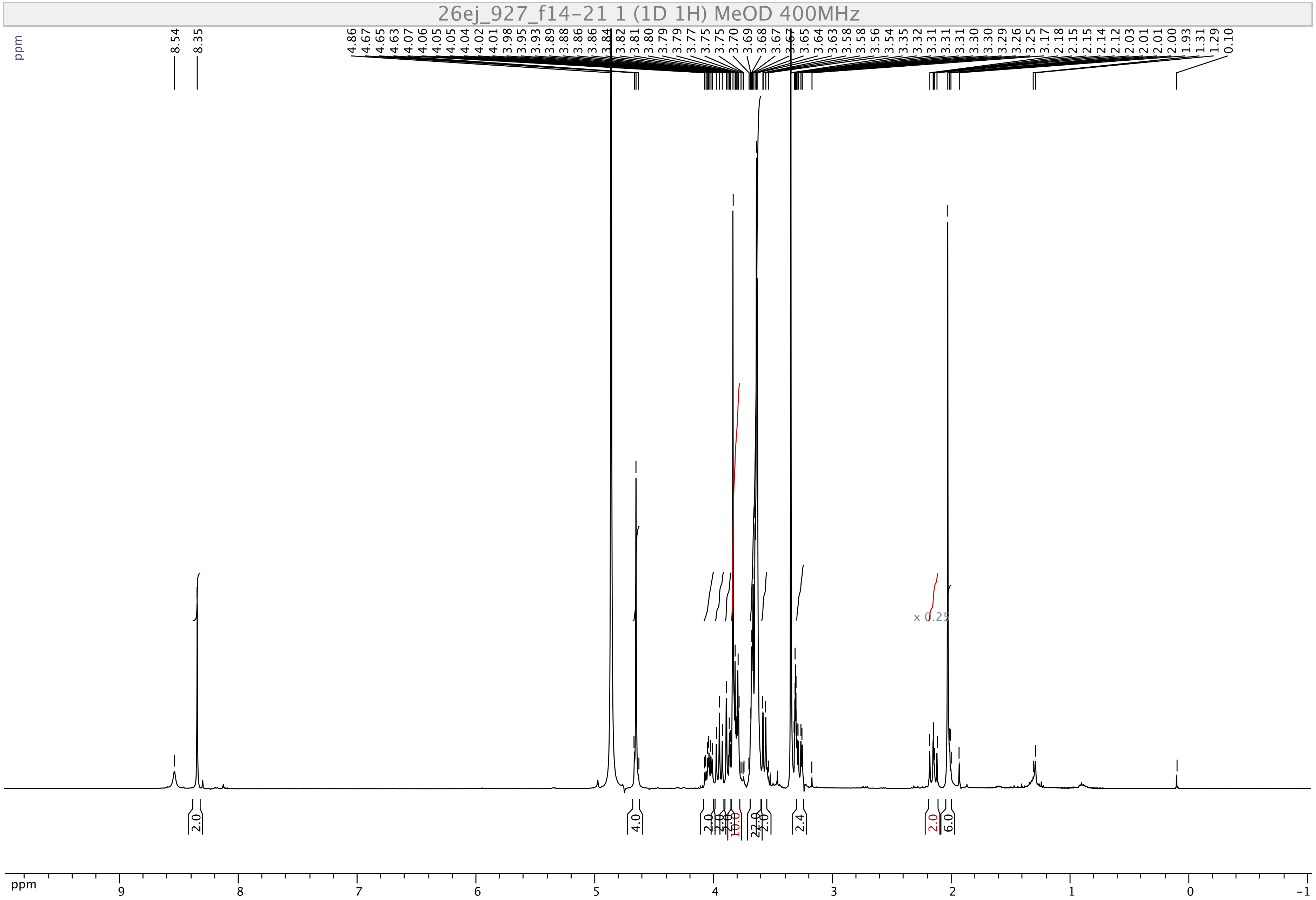
26ej_927_f14-21 1 (1D 1H) MeOD 400MHz
ppm
8.54
8.35
4.86
4.67
4.65
4.63
4.07
4.06
4.05
4.05
4.04
4.02
4.01
3.98
3.95
3.93
3.89
3.88
3.86
3.86
3.84
3.82
3.81
3.80
3.79
3.79
3.77
3.75
3.75
3.70
3.69
3.68
3.67
3.67
3.65
3.64
3.63
3.58
3.58
3.56
3.54
3.35
3.32
3.31
3.31
3.31
3.30
3.30
3.29
3.26
3.25
3.17
2.18
2.15
2.15
2.14
2.12
2.03
2.01
2.00
1.93
1.31
1.29
0.10
x 0.25
2.0
4.0
2.0
2.4
2.0
6.0
ppm
9
8
7
6
5
4
3
2
1
0
–1

Compound 39

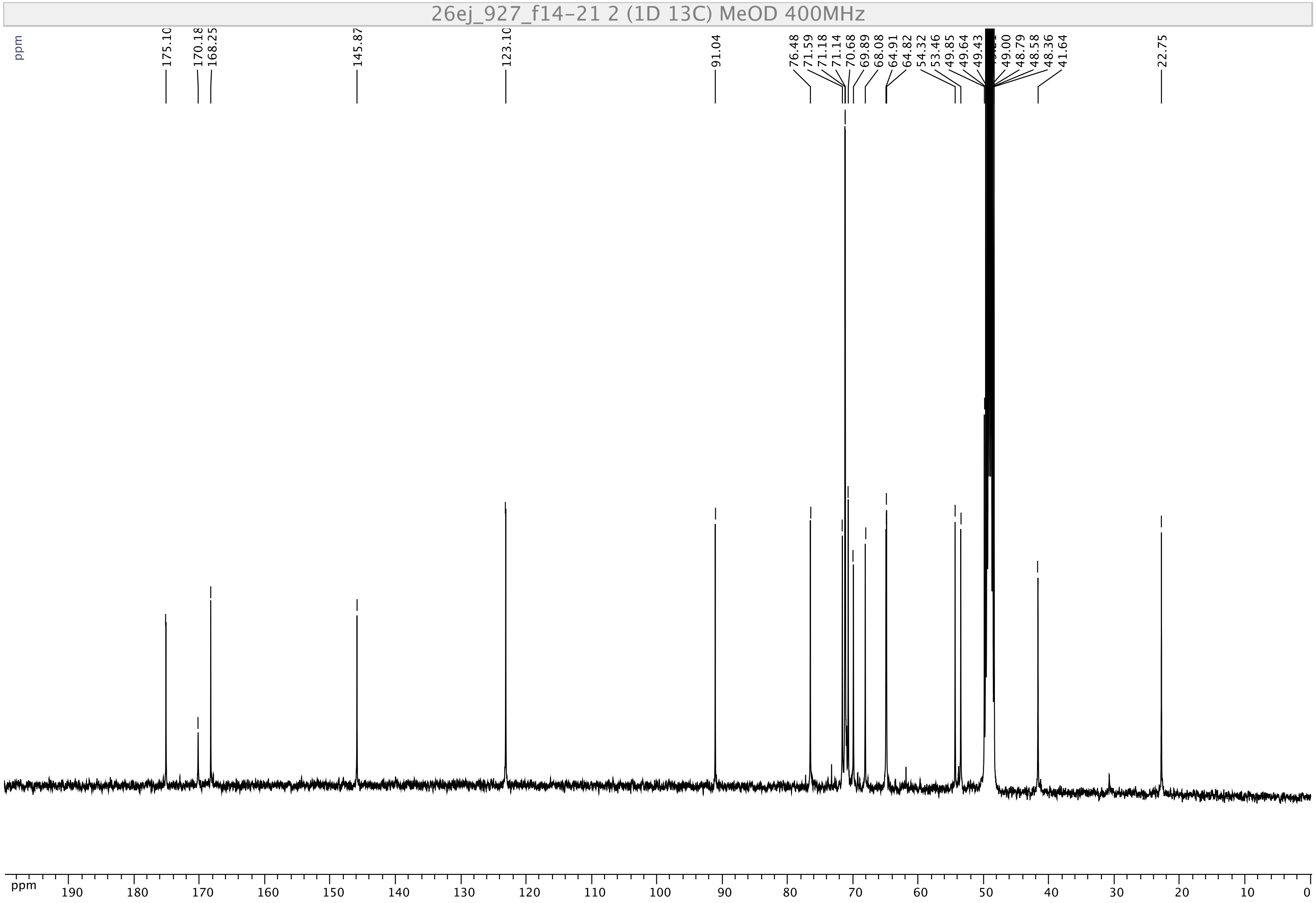

26ej_927_f14-21 2 (1D 13C) MeOD 400MHz
ppm
175.10
170.18
168.25
145.87
123.10
91.04
76.48
71.59
71.18
71.14
70.68
69.89
68.08
64.91
64.82
54.32
53.46
49.85
49.64
49.43
49.00
48.79
48.58
48.36
41.64
22.75
ppm
190
180
170
160
150
140
130
120
110
100
90
80
70
60
50
40
30
20
10
0

Compound 40 (ME1150)

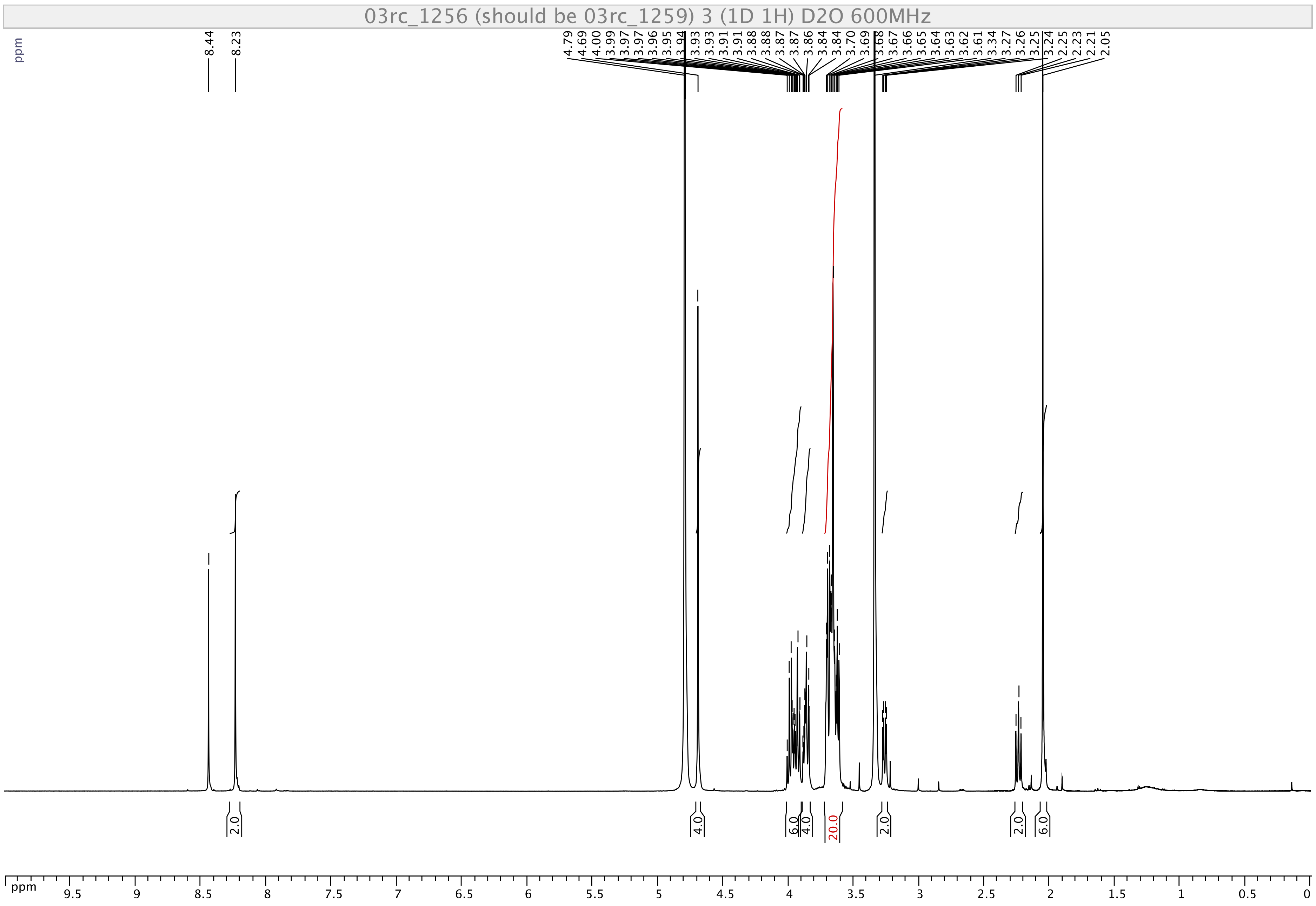

Compound 40 (ME1150)

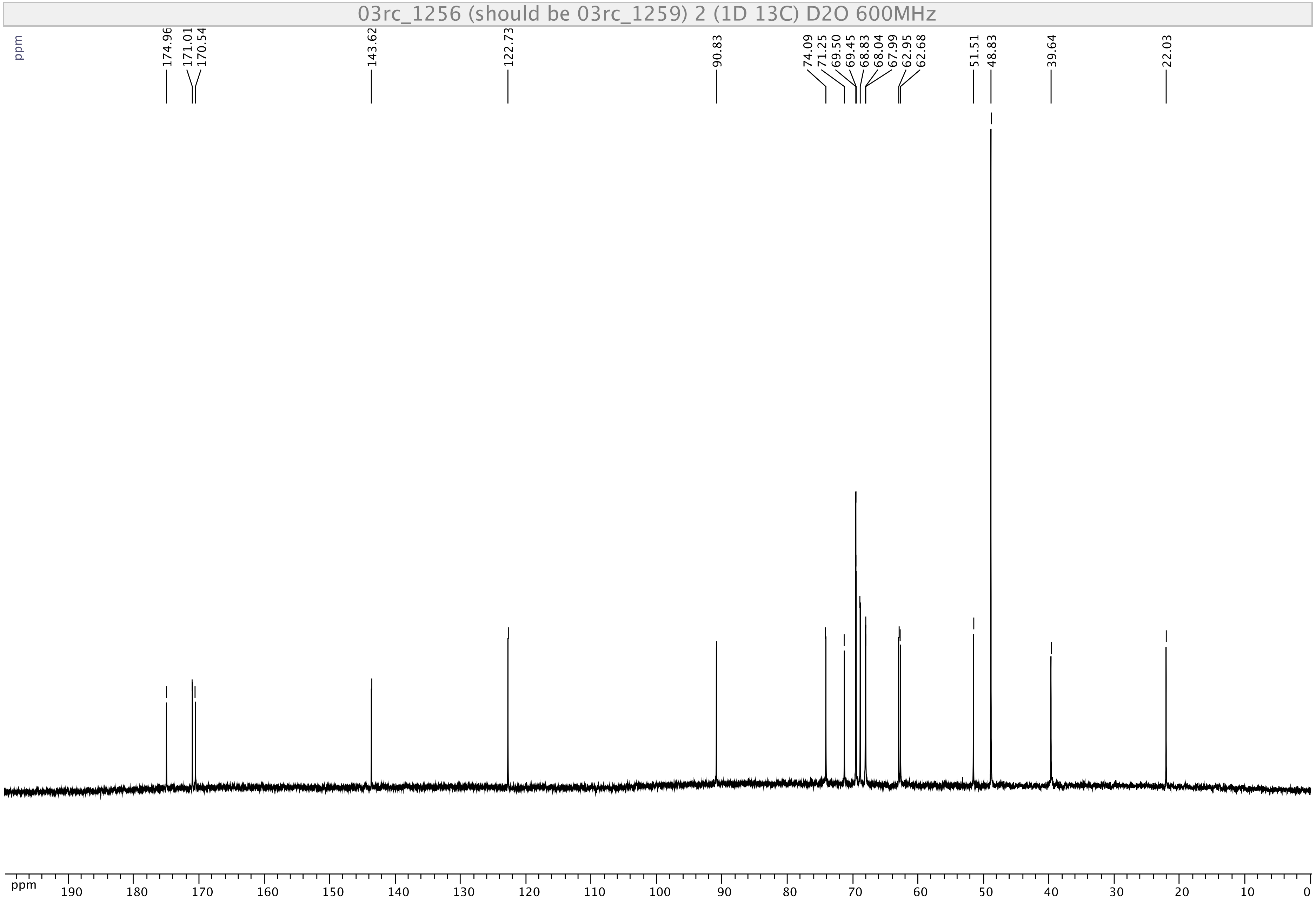

Compound 41 (ME1151)

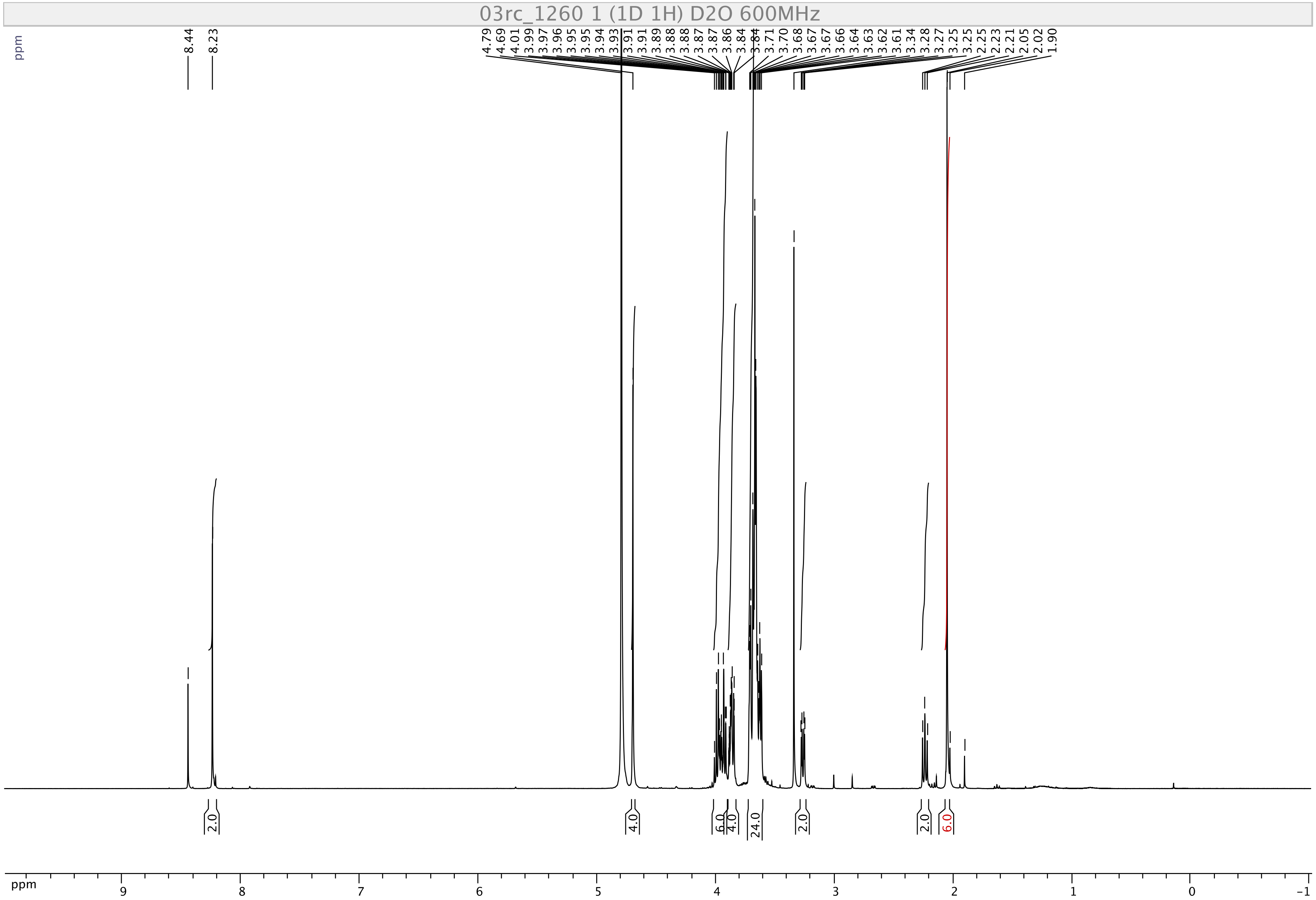

Compound 41 (ME1151)

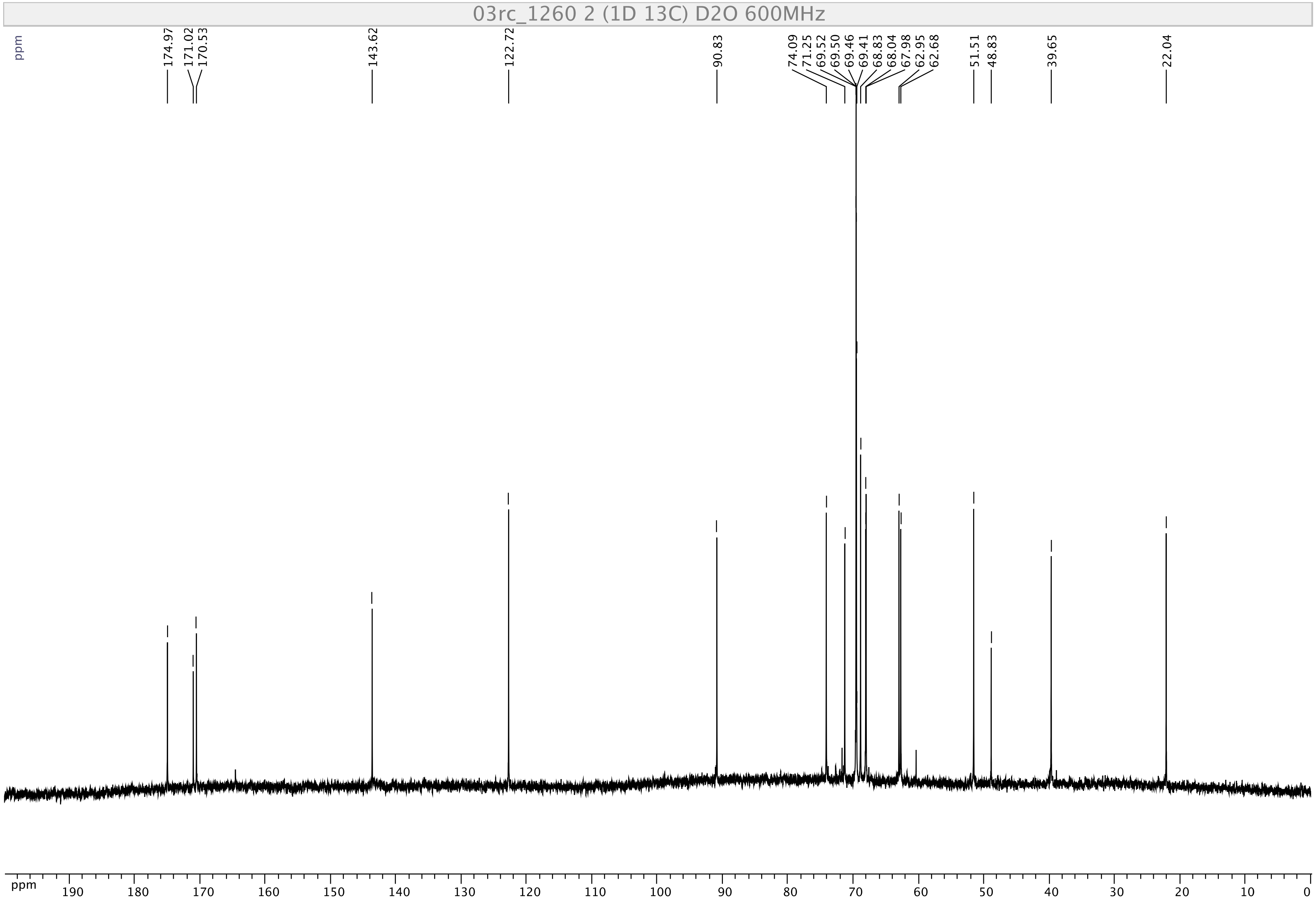

Supplement: RA-012-D1RA08968D-s001 [file RA-012-D1RA08968D-s001.pdf]
